# Risk factors for relapse and recurrence of depression in adults and how they operate: A four-phase systematic review and meta-synthesis

**DOI:** 10.1016/j.cpr.2018.07.005

**Published:** 2018-08

**Authors:** J.E.J. Buckman, A. Underwood, K. Clarke, R. Saunders, S.D. Hollon, P. Fearon, S. Pilling

**Affiliations:** a Research Department of Clinical, Educational and Health Psychology, University College London, London, UK; b Department of Psychology, Vanderbilt University, Nashville, TN, USA

**Keywords:** Depression, Depressive disorder, Major, Recurrence, Review, Risk factors

## Abstract

**Purpose:**

To review and synthesise *prognostic* indices that predict subsequent risk, *prescriptive* indices that moderate treatment response, and *mechanisms* that underlie each with respect to relapse and recurrence of depression in adults.

**Results and conclusions:**

Childhood maltreatment, post-treatment residual symptoms, and a history of recurrence emerged as strong prognostic indicators of risk and each could be used prescriptively to indicate who benefits most from continued or prophylactic treatment. Targeting prognostic indices or their “down-stream” consequences will be particularly beneficial because each is either a cause or a consequence of the causal mechanisms underlying risk of recurrence. The cognitive and neural mechanisms that underlie the prognostic indices are likely addressed by the effects of treatments that are moderated by the prescriptive factors. For example, psychosocial interventions that target the consequences of childhood maltreatment, extending pharmacotherapy or adapting psychological therapies to deal with residual symptoms, or using cognitive or mindfulness-based therapies for those with prior histories of recurrence. Future research that focuses on understanding causal pathways that link childhood maltreatment, or cognitive diatheses, to dysfunction in the neocortical and limbic pathways that process affective information and facilitate cognitive control, might result in more enduring effects of treatments for depression.

## Highlights

• Prognostic risk factors for recurrence were in order of strength of evidence:
  • 1st: childhood maltreatment, residual symptoms and history of prior episodes
  • 2nd: comorbid anxiety, rumination, neuroticism and age of onset
  • Some also may be prescriptive but evidence for such effects was limited
• Neurocognitive and neuroendocrine factors were identified as potential risk factors
• A conceptual framework considering the risk factors and mechanisms was developed.

## 1 Introduction

Depression has the highest disease burden worldwide in terms of life-years lost to disability (Prince et al., 2007). It is highly prevalent, results in significant functional impairment, and increases the risk of suicide and comorbid physical health problems (Judd, 1997; Kessler & Wang, 2009). Recurrence is common in major depression; in non-clinical cohorts a third of all persons who have at least one episode will have another (Eaton et al., 2008) and the same is true for over three-quarters of patients in clinical samples (Mueller et al., 1999). The mean number of episodes per sufferer is approximately four, with a mean duration of approximately 14–17 weeks per episode if mild in severity or 23 weeks if severe (Kessler et al., 2003). While depression traditionally has been seen as an episodic disorder with good inter-morbid functioning (Angst, Kupfer, & Rosenbaum, 1996), it is now thought by many to follow a “relapsing-remitting” course with debilitating sub-syndromal symptoms occurring between discrete episodes (e.g. Burcusa & Iacono, 2007).

### 1.1 Differentiating remission from recovery and relapse from recurrence

Current convention in the literature is to distinguish between response (better but not fully well) and remission (fully asymptomatic but still in episode) and each from recovery (the resolution of the underlying episode) (Frank et al., 1991). A further distinction is made between relapse (the return of symptoms associated with the remitted episode) and recurrence (the onset of a new episode following recovery) (Rush et al., 2006). Whether these distinctions hold in fact is still not clear but they do guide medication practice as patients are routinely kept on antidepressants (ADM) for up-to a year after reaching remission in order to forestall relapse (Reimherr et al., 1998). What will become clear in the review to follow is that they rarely guide the empirical literature.

Cognitive behaviour therapy (CBT) appears to have an enduring effect that reduces risk for relapse to the same extent as continuation ADM (Cuijpers et al., 2013) and that enduring effect may extend to the prevention of recurrence among recovered patients (Dobson et al., 2008; Hollon et al., 2005). Adding CBT as ADMs are tapered off has also been shown to reduce the risk of subsequent relapse or recurrence (Guidi, Tomba, & Fava, 2015). Even so, current practice is evolving in the direction of keeping patients with a history of recurrent or chronic depression on maintenance medication indefinitely in an attempt to delay or prevent subsequent recurrence (e.g. Thase, 2006). This is despite the suggestion that use of ADM may itself be a factor contributing to the risk of relapse or recurrence (Andrews, Kornstein, Halberstadt, Gardner, & Neale, 2011; Andrews, Thomson, Amstadter, & Neale, 2012; Fava, 2003). It is unclear whether factors other than the duration of remission differentiates those at risk for relapse from those at risk for recurrence (Farb, Irving, Anderson, & Segal, 2015). For that reason we attempt to differentiate between the two in the empirical literature.

### 1.2 Prognostic versus prescriptive designs and questions

Given that there are different treatment strategies that can be applied and different durations of treatment, the question becomes whether we can identify i) prognostic factors that indicate which patients are at greater risk of relapse or recurrence, and ii) prescriptive factors (moderators) that predict differential response to different treatments thought to help forestall or prevent relapse or recurrence (Fournier et al., 2009). Prognostic indices are best detected when treatment is held constant, ignored, or better still (from the perspective of science) not provided at all and individual differences are allowed to vary. Cohort designs are best suited to answering this question since treatment is not controlled (with those samples that receive the least treatment closest to the “state of nature”, and most informative with respect to what factors best predict relapse or recurrence.

Prescriptive designs involve the superimposition of some type of controlled treatment on top of the natural course and their proper interpretation involves testing for patient-by-treatment interactions, but even in controlled trials prognostic indices can be identified too. Within-condition comparisons among patients tells you who is most at risk (prognostic) whereas comparisons between conditions within the same kind of patients tells you what treatment works best for a given kind of patient (prescriptive). The differences in these study designs allow each to answer different albeit overlapping questions. As we shall see, reviews of the empirical literature are not always clear about which type of question is being addressed.

For a fuller explanation of prognostic compared to prescriptive indices see Supplementary Fig. 1.

### 1.3 Consensus on risk factors for relapse and recurrence

The “consensus view” as defined by Campbell's Dictionary of Psychiatry (2009) and confirmed in individual studies (e.g. Lin et al., 1998) is that two factors influence risk for both relapse and recurrence: 1) residual depressive symptoms at the end of acute treatment, and 2) a prior history of recurrence. It has also been suggested that subsequent episodes become increasingly autonomous from stressful life events (Campbell, 2009), that a lack of social support and social health problems may contribute to risk of relapse (Paykel & Priest, 1992), and that neuroticism and age of first onset are risk factors for recurrence (Gelder, Lopez-Ibor, & Andreasen, 2000). Nonetheless, despite more than half a century of active research into the nature and treatment of depression, we are still unable to predict with confidence who will relapse or recur following treatment termination (Beckerman & Corbett, 2010; Hughes & Cohen, 2009).

One difficulty in identifying more effective approaches to preventing relapse or recurrence is a lack of clarity about what it is that confers risk for each (e.g. Burcusa & Iacono, 2007). A number of studies have been hampered by methodological problems or inconsistencies (Monroe & Harkness, 2011). Early studies did not define relapse and recurrence consistently or failed to distinguish between them altogether (Beshai, Dobson, Bockting, & Quigley, 2011). The majority of more recent studies now follow the conventions set by Frank et al. (1991) and elaborated by Rush et al. (2006), but it is likely that the 8 weeks of continuous remission required by Frank was far too short and that even the 4 month criteria set by Rush may also be too short (Kessler et al., 2003). To the extent that this is true, many apparent “recurrences” would actually be “relapses”, making it harder to detect indices that predict differential risk between the two phenomena. In addition, many studies fail to discriminate patients in their first episode from those with a history of multiple previous depressive episodes. Epidemiological studies suggest that as many as half of all people who have an episode of depression will never have another (Eaton et al., 2008) and differences in the case mix across studies can lead to spurious conclusions (Monroe & Harkness, 2011). Studies in clinical samples that suggest that up to 80% of patients will have a recurrence (e.g. Mueller et al., 1999) likely oversample exactly the kinds of chronicity and recurrence that lead people to seek treatment in the first place. Further, the diagnosis of major depressive disorder (MDD) is likely causally heterogeneous. For example, it is given to those who experience prolonged periods of sadness as a reaction to a life event in one-off episodes that frequently remit spontaneously, and to those with chronic, sometimes decades-long episodes that are unresponsive to multiple treatments (Baldessarini et al., 2017; Lorenzo-Luaces, 2015). Such heterogeneity necessarily affects the ability to identify prognostic or prescriptive indices.

Given the methodological difficulties identified, it is not surprising that the field has struggled to determine what predicts risk for relapse and recurrence, whether the risk factors for each are the same or different, whether they are universal to all depression or only particular sub-types, which factors might be prognostic and which prescriptive, and what the mechanisms are by which the risk factors operate (Kazdin, 2007). This review therefore aimed to summarise and synthesise findings from studies that have reported on prognostic and prescriptive risk factors for relapse or recurrence, or that explored the mechanisms underlying the action of each, and how that evidence can guide both clinical practice and future research.

Scoping searches conducted to consider the feasibility of a meta-review of systematic reviews revealed that there were only a handful of systematic reviews of the risk factors for depressive relapse or recurrence, that each was based on only a small number of primary studies, and that very few were reviews of cohort studies. Therefore, it seemed likely that such a meta-review would only elucidate prescriptive effects on risk of relapse or recurrence and not allow us to investigate prognostic effects. Since our aim was to investigate both types of effect a novel approach was indicated. We adopted a phased approach, starting with a meta-review in order to qualitatively synthesise information across a broad literature, looking at all major types of psychiatric and psychological treatment for depression, and including cohorts of depressed participants in all community and health settings. Such meta-reviews are beholden not only to the quality of the primary studies included but also to the quality of the reviews in which they are included. However, they are considered to be of particular value when they describe the completeness (or incompleteness) of a literature, and by working at the level of reviews rather than individual studies can often generate syntheses in greater depth and with greater richness than reviews of primary studies (Francke, Smit, de Veer, & Mistiaen, 2008). This was the starting point for the present article and it led to reviews of studies that had not been included in earlier systematic reviews but themselves might contribute to our understanding of the factors that predict and the mechanisms that determine risk for relapse or recurrence. After a description of the general methods used in each of the phases of this overall review, the four phases are presented as four individual studies (Study 1–4), followed by a general discussion to summarise findings across the phases. We hoped that bringing together results like this would inform a new conceptual framework that could highlight novel ways of tackling this problem.

## 2 General methods

Four systematic reviews were conducted and are reported in accordance with the preferred reporting method for systematic reviews (PRISMA) (Moher, Liberati, Tetzlaff, & Altman, 2009).

### 2.1 Search strategy

For Studies 1–3 reviews/studies were identified by using a combination of keyword and subject heading searches on the following bibliographic databases then applying different inclusion and exclusion criteria: Cochrane Database of Reviews (searched on 8th May 2017), Embase 1947 to 2017 Week 19, Ovid MEDLINE 1946 to May Week 1 2017, Prospero (searched on 8th May 2017), PsycEXTRA 1908 to May 8, 2017, and PsycINFO 1806 to May Week 1 2017.

For Study 4 the primary studies were again identified using a combination of keyword and subject heading searches on the following databases: CAB Global Health Archive 1910 to 1972, Cochrane CENTRAL Trial Register (searched on 8th May 2017), Embase 1947 to 2017 Week 19, International Pharmaceutical Abstracts 1970 to May 2017, Ovid MEDLINE 1946 to May Week 1 2017, PsycEXTRA 1908 to May 8th 2017, and PsycINFO 1806 to May Week 1 2017.

For each study databases were searched individually and results were combined before removing duplicates. Search terms included variations of phrases such as “depression” or “major depression” or “major depressive episode” or “MDD” or “unipolar depression” or “depressive episode”, and “relapse” or “recurrence”, and searches were run in the relevant databases to include terms relevant to the type of article to be reviewed. Appendix A lists the searches conducted and results.

### 2.2 Study selection

All search results were reviewed by the lead author (JB) who read study titles to remove any clearly non-relevant articles based on the inclusion and exclusion criteria of each of the four phases listed below. The remaining abstracts were read and judged as relevant, possibly relevant, or definitely not relevant to each of the four phases based on their specific inclusion and exclusion criteria. All reviews/studies deemed to be possibly relevant were read in full and independently judged against inclusion and exclusion criteria of the individual phase by two reviewers (JB and AU). Disputes were resolved by consensus and consultation when necessary with a third reviewer (SP). Hand searches of the references from all the included reviews/studies were conducted to identify studies missed in the bibliographic searches. Relevant reviews/studies were processed as detailed above.

### 2.3 General inclusion and exclusion criteria

Reviews/studies were included if: 1) they provided data on at least one explanatory factor for relapse or recurrence of depression; 2) studies (standalone or considered in a review) had some longitudinal measurement of depressive symptoms and either a clinical interview to determine status of onset, remission, recovery, relapse, or recurrence, or used well validated interviewer-rated or self-report measure(s) of depressive symptoms; 3) they reviewed studies of adults, or included participants aged 18 years or older; and 5) were peer-reviewed journal articles published in English.

Reviews/studies were excluded if: 1) they provided no data on explanatory factors associated with relapse or recurrence of depression; 2) they reviewed or studied populations with bipolar disorder, psychotic depression, seasonal affective disorder, depression secondary to organic brain disorders, or if they focussed on relapse or recurrence of other conditions (such as drug or alcohol misuse disorders) comorbid with depression; 3) they reviewed or studied children or adolescents younger than 18 only, including studies of the impact of parental depression on children, or studies focused solely on older adults aged 65 and above or factors relevant only to geriatric populations; 5) they were not peer-reviewed journal articles; and 6) if they were not published in the English language.

Table 1 describes the research questions addressed, type of studies reviewed, specific inclusion and exclusion criteria and quality rating systems used for each review in the four studies in this article.

**Table 1:** Description of the methods of each of the four studies of the present review.

	Study 1	Study 2	Study 3	Study 4
Aims and Research Questions	Consider completeness of the literature on prognostic and prescriptive effects on relapse or recurrence, and highlight how any gaps might be addressed in future reviews and primary studies	Review prospective cohort studies of people with depression and quantitatively synthesise findings to investigate prognostic effects of risk for relapse or recurrence	To widen the consideration of risk factors and potential mechanisms not addressed in Phases 1–2 which might highlight further gaps to be filled with subsequent reviews	To investigate factors proposed to predict relapse or recurrence to depression in adults and the mechanisms of action for those factors.
Type of studies reviewed	Systematic reviews	Prospective cohort studies	Non-systematic expert, critical, or narrative reviews	Experimental or neuroimaging studies
Specific inclusion criteria	Primary studies had either a clinical interview to determine status of onset, remission, recovery, relapse, or recurrence, or used well validated interviewer-rated or self-report measure(s) of depressive symptoms	Followed remitted or recovered participants for a minimum of 12 months; continuous measurement of symptoms; used definitions or gave details of relapse and recurrence so adequate comparisons could be made across studies; at least 100 participants that relapsed or had recurrence during follow-up.^a^	Did not fit the definition for a systematic review (Higgins & Green, 2008). Studies were excluded if: they only reviewed articles covered in the systematic reviews included in Study 1.	Measured depressive symptoms over a minimum of eight weeks or they made a cross-sectional comparison between groups of remitted or recovered and relapsed or recurrent depressed participants diagnosed by a psychiatrist or with a clinical interview
Quality rating system	Primarily used AMSTAR	GRACE	N/A	GRADE

a A minimum of 100 relapses or recurrences was chosen as based on the findings from Study 1 above, if there is a prognostic effect of multiple previous episodes on the risk of relapse or recurrence it is possible that such an effect may be small in size. Based on a proposed effect size of 0.15 using GPower3 (Faul, Erdfelder, Lang, & Buchner, 2007) with an alpha level of 0.05, a sample of 100 patients who relapse is predicted to give 80% power to detect such an effect.

### 2.4 Data extraction

For Studies 1–4 data on the main results of relevance to relapse or recurrence were extracted from each review/study. In Studies 1 and 3 data on the search strategy, databases searched, terms used for searching, inclusion and exclusion criteria, as well as methods of quantitative synthesis (where applicable) were extracted from each included review, and data on the primary studies included in the review. For Study 2 data were extracted on the setting and sample included; the methods used to diagnose depression at baseline, on any relevant treatment given or measured, to capture relapse and recurrence, and how these were defined; the baseline (and if relevant, end of treatment) characteristics of the sample, and the number of participants reaching remission and recovery. For Study 4 information relevant to the methods of participant recruitment; inclusion and exclusion criteria; how depressive status or symptoms were determined at baseline; the participants; the experimental interventions or means of neuroimaging; the comparisons drawn between or within groups of participants; and the outcomes reported and the means by which these were recorded. For each Study of this review extracted data were used to build a matrix such that every proposed factor could be considered from the perspective of each included review/study, in order to consider the consistency of findings across the reviewed literature. In addition, in Study 2 data were synthesised in meta-analyses (described in detail in the method section for Study 2). In the matrix for each Study the strength of association between given risk factors and relapse or recurrence as reported in each reviewed article was entered into the relevant matrix cell in a “box score” approach. The overall strength of associations were based on our judgement after taking into consideration: the size of the effect reported in the review/study; the number of reviews/studies and sample sizes of the studies; the degree of agreement between the reviewed reviews/studies; how well the article was set up to investigate the effect; how well the article dealt with problems of bias; and the overall quality of the article. The strength of evidence was then given a rating from inconclusive (~) to very strong (+++) if positively associated with relapse or recurrence (if negatively associated factors were reversed so that they could be considered as risk factors rather than protective factors), or === if no association was found. These ratings approximately match the four categories in the column labelled “meaning” in the “Rating Study Quality” displayed in Table 2. The matrix tables display the consistency of the findings in the literature reviewed and the strength of evidence for each, but are not informed by or informative for quantitative analyses, so one cannot sum down the columns in the matrices to get an overall effect.

**Table 2:** Explanation of quality ratings.

Quality Rating	Meaning	How Determined
High quality	Further research is very unlikely to change the level of confidence in the estimate of the effect	Many more yes than no answers from AMSTAR including yes to questions of study selection and quality rating, gives reasons why studies were excluded, and if RCTs investigators were blind to allocation, and dealt appropriately with biases in methods and interpretation of results
Moderate quality	Further research is likely to have an important impact on the level of confidence in the estimate of the effect	More AMSTAR items were answered yes than no or if many more were answered yes than no but not meeting additional criteria of High Quality outlined above.
Low quality	Further research is very likely to impact upon confidence in the estimate of effect	More of the AMSTAR items were answered no than yes
Very low quality	Any estimate of effect is particularly uncertain	Very few or none of the AMSTAR items were answered yes

### 2.5 Rating study quality

Two reviewers (JB and AU) provided independent quality ratings for all included reviews/studies (Cohen's Kappa = 0.76 across all four studies) with disputes resolved by consensus or by consultation with a third reviewer (SP). Different quality rating systems were used in each of the four Studies in accordance with the type of primary studies reviewed in each. Details of the methods are given below.

## 3 Study 1 – meta-review of systematic reviews

### 3.1 Aim

The National Health and Medical Research Council of Australia (NHMRC) suggests that the greatest level of evidence for the prognosis or etiology of disorders comes from systematic reviews of prospective cohort studies, with the next greatest level of evidence coming from systematic reviews of retrospective cohort studies or randomised controlled trials (RCTs), and then pseudo-randomised trials, non-randomised trials, and case-control studies (Coleman et al., 2005). The aim of Study 1 was to conduct a meta-review of systematic reviews that reported on prognostic or prescriptive risk factors for relapse or recurrence of depression in adults, to consider the completeness of the literature, and to highlight how any gaps might be addressed in future reviews and primary studies.

### 3.2 Methods

For the purpose of this review the description of the key features given by the Cochrane Collaboration was used to define systematic reviews as studies that: “seek to collate all evidence that fits pre-specified eligibility criteria in order to address a specific research question” (Cochrane Handbook for Systematic Reviews of Interventions, Higgins & Green, 2008, p.3).

#### 3.2.1 Data extraction

In addition to the procedure and details described in the General Methods (above) for this meta-review data were extracted from each review on whether the results related only to relapse, only to recurrence, to both undifferentiated, or to both separately. These judgements were made by comparing the methods and results of the primary studies in each of the reviews against the Frank et al. (1991) and Rush et al. (2006) definitions of relapse and recurrence, so the judgement was made irrespective of whether or not the authors of the primary studies or the reviews they were included in discussed the results as related to “relapse” or “recurrence”. Supplementary Table 1 provides details for each study and Appendix B details the information on which those judgements were based.

#### 3.2.2 Rating study quality

We used “a measurement tool to assess the methodological quality of systematic reviews” (AMSTAR) to judge the quality of the included reviews (Shea et al., 2007). In order to bring a common structure to the interpretation of the AMSTAR quality ratings, we also used the categories suggested by Guyatt and colleagues in the GRADE rating system (Guyatt et al., 2008), and considered additional criteria from PRISMA (Moher et al., 2009) and the Cochrane Reviewer's Handbook (Higgins & Green, 2008). Each study fit into one of the following:

### 3.3 Results

Ten systematic reviews met inclusion criteria and were included in the present meta-review (see Supplementary Fig. 2).

#### 3.3.1 Characteristics of included reviews

Of the ten included systematic reviews, three reviewed cohort studies (Hardeveld, Spijker, De Graaf, Nolen, & Beekman, 2010; Hughes & Cohen, 2009; Kok et al., 2013), two reviewed psychotherapy RCTs intended to prevent relapse or recurrence (Beshai et al., 2011; Clarke, Mayo-Wilson, Kenny, & Pilling, 2015), two reviewed continuation pharmacotherapy (Berwian, Walter, Seifritz, & Huys, 2017; Gueorguieva, Chekroud, & Krystal, 2017), one reviewed both RCTs and quasi-randomised controlled trials of any psychological relapse prevention treatment (Feng et al., 2012), one reviewed RCTs and pseudo-RCTs with Electroconvulsive Therapy (ECT) (Bourgon & Kellner, 2000), and one reviewed both cohort studies and clinical trials of either pharmacological or non-pharmacological relapse prevention treatments (Nanni, Uher, & Danese, 2012). Two articles reviewed studies of children or adolescents as well as studies of adults; details pertaining only to primary studies of adults 18 or over were used for the present review. Full details of the methods adopted by each included review can be found in Appendix B. Details of the primary studies included in each review, the participants, interventions, comparators, and outcomes, along with the main results in relation to prognostic and prescriptive factors associated or not associated with relapse or recurrence are outlined in Supplementary Table 1.

#### 3.3.2 Review and study quality

Quality judgements were made at the level of the review, not of the primary studies included in each review. Only three of the included reviews reported on the quality of the studies reviewed therein: Clarke et al. (2015) reported that the majority of the 29 studies they reviewed were of “low quality”; Feng et al. (2012) reported that the 32 studies they reviewed were all of “good quality”; and Kok et al. (2013) reported that the four studies they reviewed were of “low-to-moderate quality”.

#### 3.3.3 Quality of reviewed studies

Of the ten reviews included three were judged to be of high quality (See Supplementary Table 2) (Clarke et al., 2015; Feng et al., 2012; Nanni et al., 2012), three were rated moderate (Berwian et al., 2017; Hardeveld et al., 2010; Kok et al., 2013), three were rated low (Bourgon & Kellner, 2000; Gueorguieva et al., 2017; Hughes & Cohen, 2009) and one was rated very-low (Beshai et al., 2011).

These ratings tell us about the quality of the reporting by the AMSTAR, PRISMA and Cochrane guidelines and therefore are primarily about the methods for dealing with bias and adherence to reporting guidelines, not the quality of the reviews in relation to the research questions of this meta-review. The reviews which best investigated the questions of prognostic effects for relapse or recurrence were Hardeveld et al. (2010), and Nanni et al. (2012), the review that best investigated prescriptive effects was that of Berwian et al. (2017) who only investigated in the context of ADM discontinuation. Although the review by Feng et al. (2012) was rated as high quality there were some concerns in the reporting of results and a conflation of studies assessing CBT delivered one-to-one (not in a group) with studies in which the primary intervention was behavioural activation (BA), interpersonal psychotherapy (IPT), or mindfulness based cognitive therapy (MBCT).

#### 3.3.4 Prognostic indicators (see Supplementary Table 1 and Table 3 for details)

##### 3.3.4.1 Residual symptoms

The evidence for residual symptoms post-treatment being associated with greater risk of relapse or recurrence was based on five reviews that together included 116 different studies, with 14,486 patients, and found high levels of agreement among the studies. Four of the five reviews primarily included RCTs [three of psychological therapies (Beshai et al., 2011; Clarke et al., 2015; Feng et al., 2012) and one of continuation ADM (Gueorguieva et al., 2017)] with the fifth reviewing only cohort studies (Hardeveld et al., 2010). In the latter review the authors reported that four cohorts found residual symptoms to be related to recurrence but three did not. Looking more closely at those primary studies it became clear that one was misclassified (Melartin et al., 2004, OR(95%CI) = 2.14(1.06–4.31), the second was a cohort in which only five participants had residual symptoms so the effect could not be adequately assessed (Ilardi, Craighead, & Evans, 1997), and the third showed a trend towards an effect but had a very small sample in which just 19 had residual symptoms (Kennedy & Paykel, 2004, *n* = 59). On the whole, there was strong evidence that residual symptoms are prognostic for subsequent relapse and recurrence.

**Table 3:** Matrix of factors associated/not associated with relapse or recurrence of depression in adults.

Study citation and quality rating	Factors investigated for their association with relapse or recurrence							
	Residual symptoms	History of previous episodes	Severity of last episode	Duration of last episode	Younger age at first onset	Neuroticism	Demographics - sex, marital status, socio-economic status, educational history	Other
Beshai et al., 2011 Very low quality	{+}	{+}					Female sex (+)	Avoidant coping {+}; Day-to-day stress and life events {+}; Interpersonal Stress +; Negative thought content {=}; Rumination +
Berwian et al., 2017 Moderate quality	(=)	(= =)	(= =)	(= =)	(= =)		(= =)	Double-depression (= =); High comorbid Anxiety (+); History of Hypomanic Symptoms (= =); Melancholic Symptoms (= =); Neuro-vegetative symptoms (=); Somatic pain (+)
Bourgon & Kellner, 2000 Low quality							= =	Delusional symptoms {~}; DST sensitivity following ECT (+) for inpatient, but (~) for outpatients; Medication resistance prior to starting ECT +; TRH stimulation ~
Clarke et al., 2015 High quality	+	+						Physical comorbidities +; Psychiatric comorbidities +; Childhood Maltreatment (+)
Feng et al., 2012 High quality	++ (~)	(=)						Frequency of sessions, group size, type of manual, and whether using take-home assignments or not, all (= =)
Gueorguieva et al., 2017 Low quality	+	= =			=		Age =; Female sex +	Shorter time until clinical response ++
Hardeveld et al., 2010 Moderate quality	+++	++	+	=	~	+	= =	Comorbid Psychiatric Disorders +; Family history of Affective Disorders =; Low social support =; Low self-efficacy +; Low self-esteem & Mastery +; Psychosocial impairment post depression +; Severe life events ~
Hughes & Cohen, 2009 Low quality			=	=	=		=	
Kok et al., 2013 Moderate quality								Comorbid Physical Illness = = =
Nanni et al., 2012 High quality					=			Childhood Maltreatment +++

Key: +++ Strong evidence for positive association; ++ good evidence of positive association; + some evidence/suggestion of positive association

=== Strong evidence for no association; == good evidence of no association; = some evidence/suggestion of no association.

~ Inconclusive evidence regarding association.

Out of ( ) if prognostic; in ( ) if prescriptive; in {} if both prognostic and prescriptive.

##### 3.3.4.2 Previous depressive episodes

One review of RCTs (Clarke et al., 2015) commented that the number of prior episodes was associated with the risk of recurrence, and another suggested that there was some limited evidence for a prognostic effect (Beshai et al., 2011). The best placed review to assess this effect (Hardeveld et al., 2010) reviewed ten articles from five different cohort studies that found a prognostic association between having history of any previous episodes and an elevated odds of relapse or recurrence (from the different cohorts *n* = 1250) [this includes one study the authors reported as having not found the effect but the reported odds ratio for recurrence was OR(95%CI) = 1.34(1.01–1.77): Holma, Holma, Melartin, Rytsälä, & Isometsä, 2008]. The review also found six other articles from five cohorts that did not find evidence of this association (from the different cohorts *n* = 571) (Hardeveld et al., 2010). One of these found a trend towards an effect [Melartin et al., 2004: OR(95%CI) =1.12(0.98–1.28)], two others were unable to adequately address the question as one was a very small cohort with high rates of prior episodes and little variability across the groups (Ilardi et al., 1997), and another was a very small cohort with only 14 people with prior episodes (Kennedy & Paykel, 2004). Interestingly, a review of four ADM discontinuation RCTs using the individual patient data from those studies (*n* = 1462) found no prognostic effect of a history of prior episodes across the dataset, irrespective of the number of prior episodes (Gueorguieva et al., 2017). Approximately two thirds of the sample were on continuation medications so this finding might be considered to be less close to the “state of nature” and so less informative of the prognostic nature of having a history of previous recurrences than the information provided from the review of cohort studies. Overall then, prior depressive episodes appear to be prognostic for relapse or recurrence.

##### 3.3.4.3 Severity of depression

There was some evidence that a more severe last depressive episode was prognostically associated with worse odds of recurrence although this was rarely assessed in the included reviews and there was conflicting evidence with some studies finding an effect and others not. In one review the effect was found in five cohorts but not in three other cohorts with no apparent pattern emerging to explain the disparity in findings (Hardeveld et al., 2010). A second review reported no prognostic effect (Hughes & Cohen, 2009) though was focussed on those receiving long-term ADM and included three cohorts also assessed in the other review. So, the evidence here is suggestive but not conclusive of the severity of depression being prognostic for relapse or recurrence.

##### 3.3.4.4 Duration of depression

The evidence for the duration of depression as a prognostic indicator of recurrence was difficult to assess as only two reviews (and few primary studies within them) investigated this. One reported that a longer duration of depression was associated with greater odds of recurrence in one large cohort but not in four smaller cohorts (Hardeveld et al., 2010) and a second review also suggested that overall there might be a lack of effect (Hughes & Cohen, 2009).

##### 3.3.4.5 Age of initial onset

Younger age of initial onset was reported in one review to be a risk factor in three cohorts but not in four others (Hardeveld et al., 2010), there was no apparent pattern to explain why some cohorts found the effect and others did not. So, the evidence for a prognostic effect was inconclusive.

##### 3.3.4.6 Neuroticism

Only one review considered the association between neuroticism and recurrence (Hardeveld et al., 2010). In that review higher neuroticism was said to be associated with a greater odds of recurrence in one cohort (Gopinath, Katon, Russo, & Ludman, 2007, *n* = 386) but not in two articles from a second cohort. However, one of these articles did find an association between higher neuroticism and recurrence [Melartin et al., 2004, *n* = 269, OR(95%CI) = 1.11(1.02–1.21)] and the other from a smaller sample of the same cohort found a trend towards an association [Holma et al., 2008: OR(95%CI) = 1.09(0.99–1.20)]. So, although neuroticism was only assessed in two cohorts, both appear to have found some evidence that higher neuroticism was associated with increased probability of recurrence.

##### 3.3.4.7 Demographics

Demographic factors such as age, socio-economic status and civil status were not found to be associated with the odds of recurrence in one review of naturalistic cohort studies (Hardeveld et al., 2010). Female sex was associated with a higher probability of recurrence (within patients discontinuing ADM) in one review (Gueorguieva et al., 2017) but not in four other reviews (Beshai et al., 2011; Bourgon & Kellner, 2000; Hardeveld et al., 2010; Hughes & Cohen, 2009). So, there appears to be little evidence of prognostic indication by demographic factors.

##### 3.3.4.8 Others

###### 3.3.4.8.1 Childhood maltreatment

There was strong evidence for a prognostic effect of childhood maltreatment (defined as physical, sexual or emotional abuse; family conflict or violence; or neglect) coming from a single high quality review (Nanni et al., 2012) in which a meta-analysis found a greater odds of recurrence for those with a history of childhood maltreatment compared to those without such histories OR(95%CI) = 2.27(1.80–2.87). All but one of the primary studies found this effect (combined *n* = 6838). The evidence here is strongly supportive of a prognostic effect for childhood maltreatment.

###### 3.3.4.8.2 Time with clinical response

There was good evidence for an association between shorter periods of time between starting ADM and reaching “clinical response”, and higher odds of subsequent relapse (for details see Supplementary Table 1) (Gueorguieva et al., 2017).

###### 3.3.4.8.3 Family history of depression

Family history of depression was assessed in just one review which found it was not associated with recurrence in five cohorts (including large community samples and small outpatient and inpatient samples), though it was associated with increased risk of recurrence in one small cohort (with a predominantly inpatient sample) (Hardeveld et al., 2010). Somewhat surprisingly, the evidence here provides little support for a prognostic effect of family history.

###### 3.3.4.8.4 Comorbidities

Overall there was some evidence that psychiatric comorbidities are associated with increased risk of recurrence. One review found that having psychiatric comorbidities was associated with greater risk in three cohorts but not in three others (Hardeveld et al., 2010). However, of these three reported to have not found an effect one found that comorbid personality disorder diagnoses were associated with relapse (*p* < .01: Ilardi et al., 1997), and another had only 32 relapses and just 5% with the comorbidity of interest so could not adequately assess the effect (Kanai et al., 2003). Another review suggested there was some limited evidence for an increased risk of recurrence in patients with comorbid psychiatric or physical disorders (Clarke et al., 2015). A third review which focussed solely on depression among those with comorbid physical health problems found no prognostic effect of such conditions on recurrence (Kok et al., 2013). Delusional symptoms were found to have a prognostic effect in studies of ECT treated patients though this was only assessed in one review and was based on just three relatively small primary studies (combined *n* = 226). Overall, there was some limited evidence for prognostic indication depending on the nature of the comorbidity.

###### 3.3.4.8.5 Psychosocial impairment and coping style

There were suggested links between psychosocial impairment and higher odds of recurrence in one review (Hardeveld et al., 2010). This was based on findings from the only two cohort studies to assess the effect. Poor coping skills were also associated with higher odds of recurrence (Hardeveld et al., 2010). This was assessed in one primary study as lower self-efficacy (Gopinath et al., 2007), and in another as lower self-esteem and mastery (Conradi, de Jonge, & Ormel, 2008). Avoidant coping style and daily hassles/life events were found to have prognostic effects on recurrence in one very low quality review (Beshai et al., 2011), although this was based on just two primary studies. The evidence was sparse but largely supportive of prognostic effects.

###### 3.3.4.8.6 Stress

The role of environmental stressors as prognostic indices were discussed in several reviews but rarely assessed directly. Higher levels of interpersonal stress were found to be associated with greater probability of relapse although this was assessed in just two primary studies in (Beshai et al., 2011). So, the evidence was sparse but somewhat supportive of a prognostic effect.

###### 3.3.4.8.7 Cognitive biases

The presence of cognitive biases, particularly rumination, was found to be associated with greater odds of relapse although the evidence for these associations was limited due to few primary studies assessing the effects (Beshai et al., 2011). Again, the evidence was sparse but supportive of prognostic indication.

#### 3.3.5 Prescriptive factors (see Supplementary Table 1 and Table 3 for details)

##### 3.3.5.1 Residual symptoms

Considering the primary studies assessed in one review (Beshai et al., 2011) there was some limited evidence that residual symptoms may moderate the effects of treatment as patients discontinuing CBT did not experience the same increased risk of recurrence as those discontinuing ADM (e.g. Paykel et al., 1999). Several studies modified the CBT delivered to focus specifically on reducing residual symptoms (e.g. Fava, Rafanelli, Grandi, Canestrari, & Morphy, 1998) and found a lower proportion of participants relapsing in the CBT or combination CBT and ADM arms of their trials relative to the ADM monotherapy or placebo arms, though some studies did not find that these differences reached statistical significance. No prescriptive effect was found ADM discontinuation studies (Berwian et al., 2017). On the whole, residual symptoms appear to moderate the differential effects of CBT over ADM.

##### 3.3.5.2 Previous depressive episodes

There was inconclusive evidence regarding moderation by previous episodes of the effect of CBT or other psychotherapies in comparison to ADM or treatment as usual (TAU) on the risk of relapse or recurrence (Feng et al., 2012). A review of ADM discontinuation studies found no moderating effect (Berwian et al., 2017). In contrast a review of psychological therapy studies (Beshai et al., 2011) presented some evidence of a prescriptive effect of previous episodes such that the active psychological therapy ameliorated the increased risk of recurrence relative to the passive/TAU comparison condition. This occurred when considering histories of three or more episodes compared to less than three in two small MBCT trials, and five or more compared to less than five episodes in one study of CBT (see Supplementary Materials Fig. 3 for a graphical representation of these effects). So, although widely accepted as a prescriptive index in the field, the quality of evidence to support this effect is relatively weak.

##### 3.3.5.3 Demographics

Sex did not moderate the effect of ADM treatments in five primary studies reviewed by Berwian et al. (2017). No prescriptive effects were found in ADM discontinuation trials for age, race or ethnicity (Berwian et al., 2017). On the whole, the evidence is largely unsupportive of a prescriptive effect by demographic factors.

##### 3.3.5.4 Others

###### 3.3.5.4.1 Childhood maltreatment

The prescriptive effect of childhood maltreatment was very rarely assessed. One RCT assessed in one review (Clarke et al., 2015) found that those with more severe childhood maltreatment were less likely to relapse if treated with MBCT relative to TAU, but not less likely to relapse if treated with MBCT compared to clinical psychoeducation, and there was no difference in the likelihood of relapse between the treatment groups for those with less severe childhood maltreatment (Williams et al., 2014). So, there is limited evidence of prescription.

###### 3.3.5.4.2 Comorbidities

Berwian et al. (2017) found higher rates of relapse in patients with most types of comorbid anxiety symptoms that discontinued ADM compared to those on continuation ADM in one primary study, although phobic anxiety and somatic anxiety symptoms did not interact with treatment effects. Patients with delusional symptoms were less likely to relapse if treated with ECT and ADM compared to ADM alone in three studies reviewed by Bourgon and Kellner (2000). On the whole, there is minimal evidence of a prescriptive effect.

###### 3.3.5.4.3 Coping style

Avoidant coping style and daily hassles were found to have a prescriptive effect in two successive reports from the same study with different lengths of follow-up (Bockting et al., 2006; Bockting, Spinhoven, Wouters, Koeter, & Schene, 2009) included in one review (Beshai et al., 2011). These studies suggested a three-way interaction with avoidant coping, treatment allocation and a history of multiple prior episodes such that a history of more prior episodes reduced the effect of avoidant coping style on risk of recurrence in the TAU group, however this history was associated with an increased effect of avoidant coping on recurrence in the preventive CBT plus TAU group. Further, reporting more daily hassles was associated with recurrence in the TAU group but not in the preventive CBT plus TAU group, and unusually, reporting more negative life events between 16 years of age and the index depressive episode was associated with higher odds of recurrence in the preventive CBT plus TAU group but not in the TAU group (Beshai et al., 2011). However, the randomised groups differed significantly both in the number or prior episodes and levels of self-reported daily hassles making this finding difficult to interpret. Overall, there is some limited evidence of a prescriptive effect but it is far from conclusive.

###### 3.3.5.4.4 Rumination

Two primary studies in one review found that patients with high levels of rumination were less likely to have a recurrence if treated with CBT or MBCT compared to ADM or TAU (Beshai et al., 2011). The evidence is sparse but suggestive of a prescriptive role for rumination.

Table 3 presents the matrix of factors associated with relapse or recurrence examined in the included reviews.

### 3.4 Discussion

Study 1 was a meta-review of systematic reviews and found that two prognostic factors were consistently associated with relapse or recurrence, with strong evidence: childhood maltreatment and residual symptoms of depression post-treatment. Childhood maltreatment was considered in only a single review but one that was of such quality and included multiple large and high quality studies that the evidence it provided was compelling (Nanni et al., 2011). The notion that residual symptoms predict subsequent relapse and recurrence is wholly consistent with “conventional wisdom” and the extant literature. There was some suggestion that both factors may act as prescriptive factors that moderate treatment effects, although childhood maltreatment was only assessed in one primary study reported in a single review (Clarke et al., 2015) and residual symptoms was only assessed in a handful of primary studies in two reviews (Beshai et al., 2011; Feng et al., 2012). So, we have less confidence with respect to each as prescriptive indices compared to their prognostic status.

A history of prior depressive episodes also appeared to be prognostic, as it was associated with greater risk of relapse or recurrence in three of the four reviews in which it was assessed (Beshai et al., 2011; Clarke et al., 2015; Hardeveld et al., 2010), but the evidence was neither as strong nor as consistent as it was for childhood maltreatment or residual symptoms, and in one instance it was only prognostic among patients who were free from residual symptoms (Judd et al., 1998). A history of prior episodes may also be prescriptive with respect to MBCT and CBT: a prescriptive effect was found in the first two MBCT RCTs, both of which were conducted in small samples by the same research group, but this effect has not been replicated since because patients with less than three episodes are now routinely excluded from those trials. Similarly, one study has shown in a post hoc analysis that CBT's enduring effect (relative to TAU) was only evident among patients with five or more episodes, but this finding has yet to be replicated (Bockting et al., 2005). A review of ADM discontinuation studies found no prescriptive effect (Berwian et al., 2017). Therefore, while a history of prior episodes appears to be prognostic and may well be prescriptive, the certainty with which this effect has been accepted in the field appears to be somewhat overstated.

Despite the widespread acceptance of the distinction between relapse and recurrence virtually none of the reviews included in this meta-review distinguished between them. The only one that did suggested that residual symptoms had a prognostic effect on relapse but not on recurrence (Beshai et al., 2011). This hypothesis is plausible and suggests that it might be worthwhile to keep the distinction between the two phenomena in subsequent research. However, due to the lack of separation between the two in the reviewed literature we now refer to relapse only when results are relevant only to relapse, and to recurrence when they might be relevant to either or both.

Other relevant findings could highlight differential mechanisms of recurrence for those who have received different modes of treatment. Patients with comorbid anxiety were particularly likely to relapse if they discontinued ADM (Berwian et al., 2017) and there was evidence of a prognostic effect of psychiatric comorbidities (including anxiety disorders) on the risk of recurrence (Clarke et al., 2015; Hardeveld et al., 2010). Somewhat surprisingly given its status in the “conventional wisdom”, only one review considered the effect of neuroticism on recurrence and this was based on just three primary studies from two cohorts (Hardeveld et al., 2010). *Re*-assessment of the primary studies suggested consistent evidence that higher neuroticism is associated with greater risk for recurrence. Age of initial depressive onset, duration of depression, and severity of the last depressive episode were not found to have prescriptive effects on recurrence in those discontinuing ADM (Berwian et al., 2017), although a more severe index episode maybe a prognostic indicator of greater odds of recurrence (Hardeveld et al., 2010). There was some limited evidence for a prognostic effect of chronic interpersonal stress being associated with greater risk of relapse (Beshai et al., 2011), and both prognostic and prescriptive effects such that those experiencing high levels of stress day-to-day responded better to CBT than to TAU (Beshai et al., 2011). There was some evidence of a prognostic effect of medication resistance pre-treatment on relapse for those treated with ECT (Bourgon & Kellner, 2000). Physical illness comorbid to depression was not prognostic for recurrence (Kok et al., 2013). Certain groups of factors related to cognitive information processing and cognitive biases, particularly rumination were associated with greater risk for recurrence prognostically and with better outcomes prescriptively for those with high levels of rumination from CBT and MBCT relative to TAU or ADM, but with very few studies assessing these effects (Beshai et al., 2011).

This meta-review had several limitations. It relied on the quality of the reviews studied rather than the quality of the primary studies reviewed therein, and by reporting results from published reviews of published studies might be even more affected by publication bias than a systematic review of primary studies. This meta-review was focussed on highlighting the completeness of the literature, identifying where gaps in the literature lie and formulating suggestions of how such gaps might be filled, so it was beyond its scope to perform meta-analyses of primary studies. Nonetheless, during the process of this meta-review it became clear that there were inconsistencies and errors in the reporting of effects in certain reviews that required a reassessment of the primary studies. Several of these “misclassifications” were due to consideration of multivariate not univariate effects. As the included reviews differed in the extent to which they relied on univariate or multivariate associations, we considered univariate effects only to avoid any inconsistencies.

There was a lack of consistent reporting on moderators of treatments. However, given the likely insufficient statistical power in many RCTs to investigate patient-by-treatment interactions a meta-analysis based on individual patient data (IPDMA) is indicated (Bower et al., 2013). One such IPDMA was included here but was limited to discontinuation of just two types of ADM in four primary studies. There was also a lack of reviews that adequately assessed the prognostic effects of many risk factors, particularly in untreated samples, and recently two large cohort studies have published new articles reporting prognostic effects on recurrence. So, a further review of appropriate cohort studies, including a meta-analysis of the prognostic effects of apparent risk factors is indicated to better address the question of risk factors for recurrence; this will be the focus of Study 2.

The two factors found to be most consistently associated with recurrence prognostically give rise to further questions: whether or not they differentially impact risk for relapse compared to recurrence is unclear; the type and intensity of both residual symptoms and childhood maltreatment sufficient to increase the risk of recurrence has yet to be determined. It is also unclear from the literature whether the absence of one or both factors is sufficient to remove the risk of recurrence altogether. Similarly, the less consistently found prognostic effect of previous episodes is also non-specific, it is unclear whether or not there is a linear or non-linear effect, and whether there is a ceiling to this. It is also unclear if the type of episode suffered in each recurrence affects the prognosis, and how treatments (whether the same continued over time or different treatments trialled singularly or in combination) differentially impact prognosis in patients with particular numbers of previous episodes. There were several factors suggested to be associated with recurrence that have the potential to help elucidate mechanisms and so could guide research into effective prevention strategies, but there were very few primary studies assessing these effects. It may be that other thematic reviews focussed specifically on these factors, may provide further information on the mechanisms and help guide thinking about how to address this issue. This will be the focus of Study 3, but first we turn to consideration of studies that might help better understand the prognostic effect of previous depressive episodes and other factors on the risk of recurrence.

## 4 Study 2 – systematic review and meta-analysis of cohort studies

### 4.1 Aim

Prospective cohort studies that follow depressed patients for years across the course of treatment while tracking outcomes like remission and recovery are particularly well suited to investigate prognostic risk factors for recurrence of depression. They are especially suited to consider whether there are differential risk factors for relapse versus recurrence (for example, whether number of prior episodes predicts recurrence but not relapse). The aim of Study 2 was therefore to review prospective cohort studies of adults with depression.

### 4.2 Methods

We used the same searches and procedures that we used in Study 1 but applied a different set of inclusion and exclusion criteria (see Table 1) to identify cohort studies reporting on prognostic factors associated with relapse or recurrence of depression and to extract their data for both qualitative and quantitative syntheses.

#### 4.2.1 Quality ratings

Quality ratings were made based on the GRACE checklist (Dreyer, Velentgas, Westrich, & Dubois, 2014), details of which can be found in the Supplementary Materials preceding Table 3.

#### 4.2.2 Quantitative synthesis

Meta-analyses were conducted for each prognostic index using Review Manager (RevMan) Version 5.3 (http://ims.cochrane.org/revman) (Cochrane, 2008) with studies grouped based on whether or not they reported on the risk factor of interest. If more than one study from the same cohort and using the same sample (rather than different subsamples) reported on the risk factor only the study with the highest sample size was chosen with others from the same cohort excluded from the given analysis. When sufficient data were provided in the published articles or any online supplementary materials accompanying the articles these were entered into the meta-analyses or information from these sources was used to derive the appropriate measure of effect for each study. When this was not possible authors were contacted for unpublished data from their studies. As discussed above we chose to use univariate, unadjusted measures of effect. The measures of effect were entered into random effects meta-analyses using the generic inverse variance method (DerSimonian & Laird, 1986) to derive summary odds ratios or hazard ratios and their 95% confidence intervals. If both an odds ratio and hazard ratio were given for the same risk factor in a minimum of two studies from different cohorts, separate meta-analyses were conducted for each measure of effect.

Heterogeneity was assessed using the tau-squared statistic and risk-of-bias judgements were made using the GRACE quality ratings as described above. Publication bias was assessed using funnel plots. Statistical adjustments for potential reporting bias were not made given the small number of studies included in each meta-analysis and the lack of evidence for the direction of any potential publication or reporting bias on the effect of the given risk factors. However, sensitivity analyses were planned such that low quality studies would be removed and if two studies from the same cohort used different means of defining or measuring the risk factor of interest the one excluded from the primary analysis would replace the other study from the same cohort, in order to consider the consistency of the findings. Further sensitivity analyses were planned to remove studies that reported only multivariate data to consider the unadjusted effects of the risk factors on recurrence.

### 4.3 Results

Twelve studies were identified as meeting inclusion criteria from the bibliographic database searches and were included in the present review (see Supplementary Fig. 4).

#### 4.3.1 Characteristics of included studies

Of the twelve reviewed articles six were from the Netherlands Study of Depression and Anxiety (NESDA) and a further three were from the Collaborative Depression Study (CDS), so although the individual studies sometimes drew different subsamples or included different periods of follow-up there were only five separate cohorts represented in the articles reviewed (see Table 4 and Supplementary Table 3 for details). The cohorts were drawn from populations in only two countries, the USA [CDS, NESARC and the Group Health Cooperative (GHC: Gopinath et al. (2007) study] and the Netherlands (NESDA and NEMESIS). The USA cohorts tended to draw on more severely impaired clinical populations that required participants to be seeking or to have sought treatment, with high rates of inpatient care in the CDS and participants at high risk of recurrence due to their clinical presentations in the GHC study. In contrast, the Dutch cohorts included community samples of depressed people not seeking or receiving treatment, and drew more from primary care than specialist care centres. Both NESDA and CDS limited their study populations in terms of ethnicity either indirectly as in NESDA by requiring all participants to be able to speak fluently in Dutch or directly as in CDS which only recruited white English speaking American adults. All studies excluded participants at baseline if they had diagnoses of Bipolar disorder or psychotic conditions though some of the CDS studies kept participants in the cohort if their episodes were reclassified later as Bipolar or Schizoaffective. The CDS cohort stands apart from the others by having up to 15 years of completed follow-up compared to no more than six years for the other cohorts. Only the GHC study controlled treatment (it was a cohort formed from an RCT that found no main effect for treatment) and the CDS measured ADM or ECT received weekly but did not control treatment. None of the studies measured any psychological therapies received either historically or during follow-up.

**Table 4:** Factors associated and not associated with recurrence to depression in adults reported in each of the included non-systematic reviews, and proposed mechanisms of action.

Study citation	Factors investigated for their association with recurrence								Proposed mechanisms of action
	Stressful Life Events	Neuroendocrine or HPA Axis Dysregulation	Sleep Dysregulation	Cognitive Reactivity	Information Processing Biases	Cognitive Biases	Rumination	Other	
Beckerman & Corbett, 2010					++	++			Depressive recurrence may be due to faulty thought processing and attentional control.
Belsher & Costello, 1988	++	++						Duration of wellness after last episode ==	None stated
Bockting et al., 2015				~		++			None stated
Burcusa & Iacono, 2007	++								None stated
Costa e Silva, 2004								Decreased Neuroplasticity ++	Alteration of metabolism or atrophy in neural structures involved in the control of mood and emotions may play a key role in the etiology of depression.
Carvalho, Sharma, Brunoni, Vieta, & Fava, 2016		++							Early life stress leads to permanent changes in the HPA axis and may lead to the development of depression in adulthood
Dedovic & Ngiam, 2015		++							None stated
Farb et al., 2015		++			++	++	++		Fixation on negative features of life events and dysphoric rumination co-occur during depressive episodes becoming mutually reinforced as a pattern of response to events. This leads to sensitization to adverse events with minor life events leading to fixation on negative aspects of the events triggering powerful dysphoric elaborations about the self, the future, and the world and hence to depressed mood.
Hammen, 2003	++								Maladaptive cognitions about attachment and dysfunctional interpersonal skills contribute to the stress generation and hence recurrence.
Hick & Chan, 2010				++			+		Small change of mood to one that is dysphoric may trigger negative attitudes, thinking patterns and beliefs, and these may be underlined by rumination. When this happens negative feelings worsen and intensify and magnify negative thoughts and feelings. This may generate even more overwhelming negative thoughts and negative attitudes setting off a depressive recurrence.
Hollon, Thase, & Markowitz, 2002					+	++		Hopelessness =	Change in readily accessible beliefs and expectations may mediate the reduction of acute distress, whereas change in underlying information processing may play a larger role with respect to the prevention of recurrence.
Keller, 1996								Double depression ++ Poor symptom control during continuation phase treatment +	None stated
Kerr et al., 2013	+				++	+	++		Body-focused attentional practice enhances localized attentional control, thought to play a key role in regulating sensory input to sensory neocortex and in enhancing signal-to-noise properties across the neocortex, thus acting against the information processing biases apparent in depressed and formerly depressed individuals which otherwise negatively impact on their ability to utilize social interactions and support and therefore increase their risk of recurrence.
Kessler, 1997								Younger age ++; Demographics = =	Inventories of stressful events predict subsequent depression. Partly due to events causing depression but the reverse is also true. The association between stressful life events and depression is moderated by prior characteristics of the people exposed to the events and the environments in which these people are embedded.
Lau et al., 2004				++	++	++			Cognitive reactivity is an independent predictor of depressive recurrence; increased negative cognition(s) manifest during induced sad mood or a naturally occurring mild depressed mood is predictive of depressive recurrence.
Liu, 2013	++							5-HTTLPR genotype interacting with relational attachment security +	Genetic factors moderate the relationship between behavioural risk factors and recurrence of depression. Stress generation is tentatively proposed to provide the mechanism for this: Dependent stress may interact with the underlying depressogenic vulnerability that produces it, thereby elevating risk for depression beyond what may be accounted for by either variable alone.
Lopresti et al., 2014		++							None stated
Metcalf & Dimidjian, 2014				~	+		++		MBCT proposed to reduce risk of recurrence by decreasing cognitive reactivity to negative emotions through regular mindfulness practice including thoughts that lead to both neutral and negative emotional reactions.
Modell & Lauer, 2007			++						Dysregulated REM sleep affects risk for recurrence mediated by the noradrenergic, serotonergic, and cholinergic systems, and considerable genetic control. Alteration in CREB gene increasing REM sleep, REM sleep is normally inhibited during brain maturation, a genetic predisposition to the lack of this inhibition may increase risk of recurrence.
Monroe & Harkness, 2005	++								The stress sensitization model predicts that life events meeting minimal criteria for triggering recurrence are even more common given stress generation. In contrast, for the stress autonomy model, stress generation would have less powerful implications over time, given the proposed uncorrelated eventual relationship between life stress and recurrence.
Palagini et al., 2013			++						Genetic factors include cholinergic receptors, circadian rhythm genes and orexinergic mechanisms affect REM sleep latency. REM sleep may help regulate affective reactivity and emotional information processing. Stress-related REM sleep hyperactivation/disinhibition could affect adult neurogenesis and thus might endanger hippocampal integrity, thereby contributing to the development of mood disorders through allostatic load.
Robinson & Sahakian, 2008	+		+		+	+	+		Different symptoms of depression involve different patterns of neural activation that become associated during a first episode and that this could underlie the “kindling” effect.
Scher et al., 2005				++	++				Cognitive vulnerability may contribute to depression recurrence and that vulnerability may originate in part from interactions that occur within the context of childhood attachment relationships.
Scott, 2001					+	++		Extreme Response Style (on self-report questionnaires) ++	The way depressed patients process depression related material, rather than the content of their thoughts may be critical in preventing recurrence.
Segal et al., 1996					++				Once activated, depression-related patterns of processing may lead to behavioural processes that in turn increase risk of recurrence.
Sipe & Eisendrath, 2012			+			+	+	High amygdala reactivity and dysfunction in cortico-limbic circuits +	Catastrophic ruminations and threat avoidance may be accompanied by limbic dysfunction. Mindfulness may interrupt the cycle of rumination about past regrets or future fears, and enhance self-compassion, breaking the link between cognitive reactivity and escalating depressive symptoms.
Slavich & Irwin, 2014		++						Interpersonal stress in general +, particularly if including social rejection ++	The role of inflammation in stress sensitization could be due to exposure to early adversity and successive depressive episodes potentiating the SNS and HPA axis in response to stress, galvanizing the regulatory link between the brain and the inflammatory system. The authors speculate that this results in a decreasing threshold for the magnitude of stress sufficient to trigger inflammatory responses and evoke depressive episodes.

Key: ++ good evidence of positive association with recurrence; + some evidence/suggestion of positive association with recurrence; = = good evidence of no association with recurrence; = some evidence/suggestion of no association with recurrence.

Abbreviations: CT – cognitive therapy; HPA – hypothalamo-pituitary-adrenal; REM – rapid eye movement; SSRI – selective serotonin reuptake inhibitor; ST – standard.

#### 4.3.2 Study quality

Study quality was assessed using the GRACE checklist (Dreyer et al., 2014), no overall quality descriptions were assigned unlike the quality ratings of the systematic reviews included in Study 1 above, see Supplementary Table 4 for details.

#### 4.3.3 Qualitative synthesis (see Supplementary Table 6).

##### 4.3.3.1 Childhood maltreatment

A prognostic effect of a history of childhood maltreatment being associated with greater odds of recurrence was endorsed by all five of the studies across four cohorts to have assessed this (Gopinath et al., 2007; Hardeveld et al., 2013a; Hardeveld et al., 2014; Hardeveld et al., 2015; Wang et al., 2014).

##### 4.3.3.2 Residual symptoms

All studies that assessed the effect of residual symptoms found evidence of an association with increased risk of recurrence.

##### 4.3.3.3 Previous depressive episodes

Seven studies across four cohorts found evidence of a prognostic effect of previous depressive episodes on the odds or hazard of recurrence. Three of these compared any previous episodes to none (Hardeveld et al., 2013a NEMESIS; Hardeveld et al., 2013b NESDA; Hardeveld et al., 2014 NESDA), two from the CDS reported an increase in risk with each consecutive episode (Mueller et al., 1999; Solomon et al., 2000), one measured a history of three or more compared to less than three episodes (Gopinath et al., 2007 GHC), and one measured a history of four or more compared to less than four episodes (Judd et al., 1998 CDS). Two studies did not find an association between the odds of recurrence and having two or three compared to just one previous episodes (not compared to zero previous episodes). One only assessed the effect in participants that had been in remission for at least six months (Spinhoven, Drost, de Rooij, van Hemert, & Penninx, 2016) and the other found that the variable was still important in a predictive model of recurrence (Wang et al., 2014). Further, when calculating the odds ratios for previous episodes from the study by Mueller et al. (1999) there was a considerable difference between the comparison of any or no previous episodes, two compared to one previous episode, and of at least three compared to less than three previous episodes (see Supplementary Table 5). In addition, two of the CDS studies reported that the effect was impacted by a third factor. In one CDS study those without residual symptoms were at only a modestly increased risk of recurrence if they had a history of four or more episodes (Judd et al., 1998), and in another those with five or more depression free years were not at increased risk of recurrence even if they had a history of multiple previous episodes (Mueller et al., 1999). Unpublished data from the authors of the Spinhoven et al. (2016) NESDA study support this latter finding as comparing any to no previous episodes was associated with a considerably higher odds of recurrence than restricting this comparison to cohort members that had been in remission for at least six months; the effect estimate was reduced by approximately 27%. So, it appears that the prognostic effect of prior episodes on recurrence is strongest when comparing any to no prior episodes, in the absence of residual symptoms, and when the period of recovery is less than five years. In the converse of these conditions the prognostic effect is either considerably weaker or absent altogether. Further, the “conventional wisdom” that there is an important increase in risk of recurrence with each subsequent depressive episode appears not to be all that well supported by the reviewed studies.

##### 4.3.3.4 Severity of depression

More severe symptoms of depression at the start of an episode were associated with greater probability of recurrence in GHC, NEMESIS, and NESDA but not in CDS.

##### 4.3.3.5 Duration of depression

A longer duration of the index episode was associated with greater odds of recurrence in CDS as was longer duration of the longest ever past depressive episode in GHC, but the percentage of months with depression in the past year was not associated with recurrence in NESDA.

##### 4.3.3.6 Age of initial onset

Only two cohorts assessed age of onset, one found that a younger age of first onset was associated with shorter time to recurrence (NEMESIS), and there was a borderline effect for this in NESDA.

##### 4.3.3.7 Family history of depression

Findings were inconsistent with respect to family history of depression; it predicted increased risk for recurrence in NESARC but not in GHC or NESDA.

##### 4.3.3.8 Neuroticism

Higher neuroticism predicted recurrence in GHC (as reported in Study 1 above). In NEMESIS and one NESDA study there were small univariate but not multivariate effects (Hardeveld et al., 2013a NEMESIS; Spinhoven et al., 2016 NESDA) and another NESDA study found no univariate association between neuroticism and the hazard of recurrence (Hardeveld et al., 2013b).

##### 4.3.3.9 Demographics

Younger age at baseline was associated with greater likelihood of recurrence in NEMESIS but not in CDS, NESARC or NESDA. Marital status, race and gender predicted recurrence in NESARC but not in two different samples of the NESDA cohort (Hardeveld et al., 2014; Spinhoven et al., 2016).

##### 4.3.3.10 Comorbidities

A recent history of anxiety disorders (in NESDA and NESARC) and symptoms of fear or panic (GHC) were associated with greater odds of recurrence, although unlike other anxiety disorders comorbid panic disorder was not associated with recurrence in those that had been in remission for at least six months (NESDA: Spinhoven et al., 2016). Comorbid anxiety disorders were not related to greater speed of recurrence in NEMESIS (Hardeveld et al., 2013a). There was some evidence of an association with increased risk of recurrence for: multiple physical symptoms (NESDA: Dijkstra-Kersten et al., 2017), chronic pain grade, the total number of chronic pain locations and particularly neck, chest, or abdominal pain (NESDA: Gerrits et al., 2014); comorbid physical health problems (NESDA); and avoidant personality disorder (NESARC).

##### 4.3.3.11 Others

###### 4.3.3.11.1 ADM medication

Adherence was associated with lower odds of recurrence in the one study to assess this (GHC), being on ADM was not associated with recurrence in CDS but was in NESDA. Given that the latter study sampled predominantly from community settings this could suggest that being on ADM was a proxy for risk of recurrence in that only those most at risk were given ADM.

Other factors found to be associated with increased risk of recurrence but only assessed in a single cohort were: rumination, worry, experiential avoidance, psychosocial difficulties (all NESDA); lower self-efficacy (GHC); elevated cortisol awakening response (NESDA); and physical, racial and sexual abuse in adulthood (NESARC).

###### 4.3.3.11.2 Quantitative syntheses

**Fig. 1:**
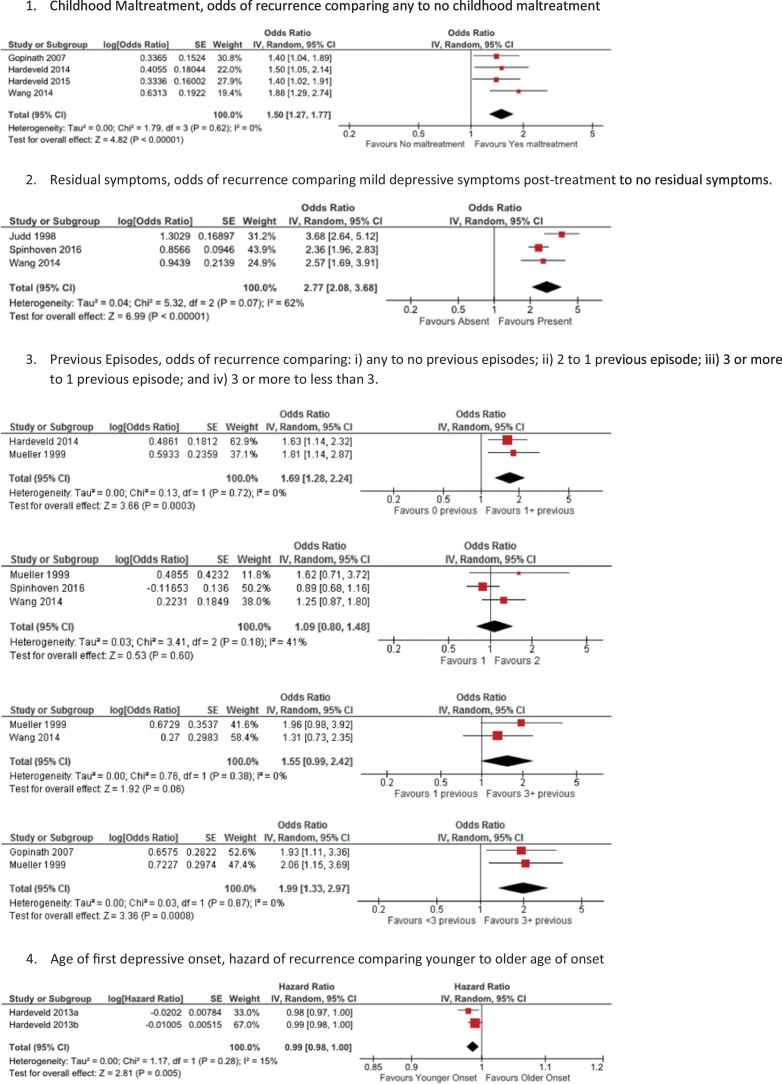

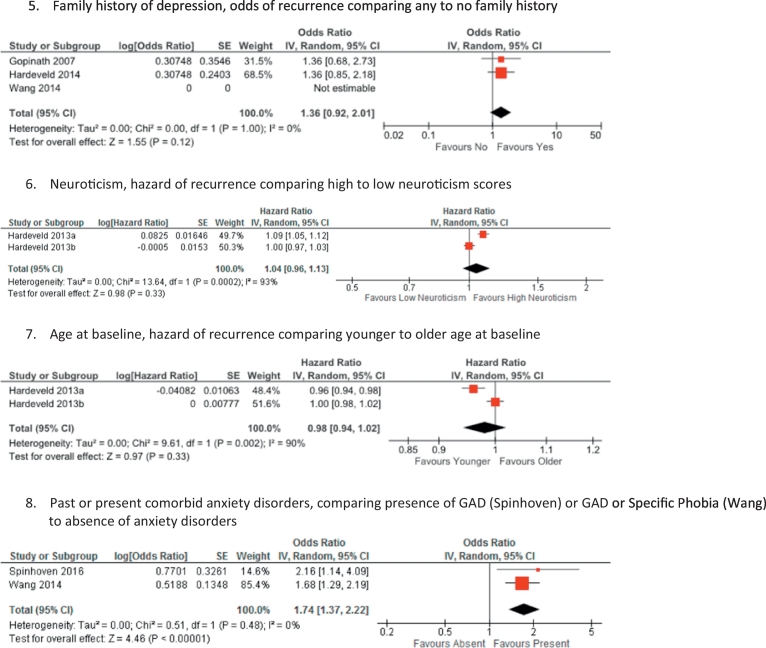
Forest plots from Generic Inverse-Variance meta-analyses of prognostic risk factors for relapse or recurrence of depression.

There were sufficient data from the reviewed studies on eight prognostic risk factors to combine in meta-analyses (Fig. 1). The data for each factor were sometimes reported as odds ratios and other times as hazard ratios (only number of prior episodes was reported using both). Greater heterogeneity was observed among studies evaluating previous depressive episodes, residual symptoms, neuroticism and age at baseline, with very little heterogeneity in studies evaluating childhood maltreatment, family history of depression, and age at first onset. On the basis of the combined summary statistics, the presence of residual depressive symptoms, a history of childhood maltreatment, a history of previous episodes (when comparing any to zero previous episodes and when comparing more than three to less than three previous episodes), a younger age at first depressive onset, and past or present comorbid anxiety disorders were associated with a greater likelihood of recurrence. A history of two compared to one, or three or more compared to one previous depressive episode, higher neuroticism, a family history of depression, and younger age at baseline were not significant risk factors. Summary statistics could not be calculated for baseline symptom severity as measures of effect were different across the studies, no measures of effect were reported or calculable for duration of the index episode, and although two studies reported hazard ratios for being on ADM they were from the same sample so were not combined.

###### 4.3.3.11.3 Sensitivity analyses

Sensitivity analyses were conducted for all of the analyses involving data from the NESARC cohort which included more than one other study, as only multivariable results were published in Wang et al. (2014) and all other studies reported univariate results for each factor. No sensitivity analyses were performed removing studies based on their GRACE quality ratings as there would have been insufficient data to quantitatively synthesise results across the remaining studies. Sensitivity analyses were conducted to assess: 1) The odds of recurrence for two compared to one previous episode deleting data from the NESARC cohort (Wang et al., 2014), this resulted in an OR(95%CI) = 1.05(0.62–1.79), that was marginally lower than in the primary analysis; 2) the odds for residual symptoms removing data from the NESARC cohort resulted in an OR(95%CI) = 2.88(1.86–4.45), marginally higher than in the primary analysis; 3) the odds of recurrence for childhood maltreatment removing data from the NESARC cohort resulted in an OR(95%CI) = 1.43(1.19–1.71), again slightly higher than in the primary analysis; and 4) The odds of recurrence for childhood maltreatment substituting data from the self-report factor recording being left alone often before the age of ten, instead of using the factor reporting being abused before the age of 18 from the Wang et al. (2014) study. This reduced the size of the overall effect but did not change the direction of the summary estimate [OR(95%CI) = 1.45(1.22–1.72)], compared to [OR(95%C) = 1.50(1.27–1.77)] in the primary analysis. 5) The hazard of recurrence of any compared to no previous episodes, as with the combined odds ratios, suggested a clear prognostic association between a history of any previous depressive episodes and recurrence HR(95%CI) = 1.64(1.25–2.14). 6) The odds of recurrence associated with current or past different anxiety disorders substituting the odds related to GAD from Spinhoven et al. (2016) for: i) social anxiety disorder [OR(95%CI) = 1.90(1.40–2.58)]; ii) agoraphobia [OR(95%CI) = 1.76(1.38–2.23)]; and iii) panic disorder [OR(95%CI) = 1.62(1.29–2.05)], see Supplementary Fig. 5.

### 4.4 Discussion

Study 2 was in general agreement with Study 1 in that the strongest evidence for prognostic risk factors for recurrence to depression appeared to be for childhood maltreatment and residual depressive symptoms. Recurrent patients with a history of prior episodes were clearly at elevated risk compared to patients in their first episode, but risk did not appear to increase much with each successive episode; it remained relatively static. Risk was thought to increase as a function of an increasing number of episodes in the CDS (Mueller et al., 1999; Solomon et al., 2000) and those findings largely defined the consensus in the field. However, as shown in Supplementary Materials Figs. 3a-3e only slightly higher percentages of participants had another recurrence with each subsequent episode observed prospectively, and with each further prior episode pre-baseline, and these recurrences occurred increasingly quickly. The increase in percentages after the first recurrence was largest in going from one to two recurrences or prior episodes, with small increases beyond that. In addition, the odds of having a recurrence did not increase significantly when comparing each additional prior episode to one fewer prior episode, or each subsequent episode measured prospectively, suggesting a monotonic function with diminishing increments with each episode. Only CDS gave sufficient data to consider these effects as the other studies dichotomized on the number of prior episodes, likely due to sample size. Overall, it appears that risk increases with each successive episode but by far the largest increment is associated with the increment from zero (not recurrent) to one or more prior episodes (recurrent) and that subsequent increments associated with successive episodes are modest at best and decline in magnitude. In addition, a history of prior recurrence was most prognostic among patients who were in full remission (residual symptoms trumps recurrence) (Judd et al., 1998) and not among patients recovered for at least five years (Mueller et al., 1999).

Family history of depression and age at baseline were not associated with increased likelihood of recurrence. There was some support for a prognostic effect of neuroticism although the findings were inconsistent across the reviewed studies. Unlike in Study 1, age of first onset was found to be associated with a marginal increase in the hazard of recurrence, and it seems likely that increased severity of the index depressive episode and longer durations of depression are also associated with increased risk of recurrence. A recent or lifetime history of comorbid anxiety disorders was associated with increased probability of recurrence although there was not universal agreement on which disorders and symptoms are associated with recurrence. There was some evidence that the following were also associated with greater probability of recurrence, albeit with evidence from only one cohort for each: avoidant personality disorder; a high degree of fear or panic symptoms; rumination; worry; experiential avoidance; multiple physical symptoms; chronic pain (grade, number of locations and overall and various specific locations) and other comorbid physical health problems; psychosocial difficulties; lower self-efficacy; racial, physical or sexual abuse in adulthood; and higher cortisol response.

There were virtually no data on relapse as all the reviewed studies measured recurrences in terms of the Rush et al. (2006) definitions. Therefore there was no way to investigate the hypothesis that multiple previous episodes might be a risk factor for recurrence not relapse. The reviewed cohort studies found very good evidence for residual symptoms as a risk factor for recurrence and it is likely that this is relevant to both relapse (as found in Study 1) and recurrence, contrary to the hypothesis that residual symptoms are only relevant to relapse and prior episodes only relevant to recurrence.

The findings on previous episodes of depression add weight to the findings of Study 1 above in that overall the prognostic effect appears to be strongest when comparing any to no previous episodes but that there is some inconsistency beyond that, with only modest support for a linear increase in the risk as the number of episodes increase (for more detail see Supplementary Discussion).

A number of other factors were also found to predict risk of recurrence. Not adhering to the ADM treatment regime or not being in receipt of ADM was associated with greater risk of recurrence in the clinical samples when medication use was closely monitored, though being prescribed ADM was associated with a greater hazard of recurrence in a mixed community and clinical setting sample, perhaps representing a proxy for risk in this setting. This fits with clinical lore on the importance of continuation and maintenance ADM and their role in treatment offered by specialised mental health services. Finding a prognostic effect for chronic pain was somewhat at odds with the findings from Study 1 although the study reviewed here offered greater specificity on the effects by considering types, degrees and locations of pain, which might explain the discrepancy. Some of the findings from the cohort studies were more contradictory to the findings of Study 1 in that there was some evidence to suggest that younger age of initial depressive onset, and severity of the index episode, were associated with greater odds of recurrence. There was also some evidence that female sex, and the duration of episodes were associated with recurrence but the evidence was not consistent and could have been due to a lack of appropriate adjustment for potential Type I errors.

Overall, only twelve articles from five different cohorts met our inclusion criteria. As shown in Supplementary Fig. 4 many other articles were considered including those from well-known large-scale cohort studies but most did not have continuous measurement of symptoms throughout follow-up, some were based on children and adolescents rather than adults, and others did not report risk factors for recurrence. So, the included studies only drew samples from the USA and the Netherlands and most restricted the ethnicity of their samples either directly or indirectly. This opens up our findings to potential problems of generalisability to the wider population of people at risk of recurrence, and to potential biases associated with publication and reporting. Inspection of the funnel plots suggested relatively robust findings but it is likely that whatever publication and reporting biases exist would have pulled results towards the null. This might perhaps have resulted in fewer prognostic indicators being found or less strong support for those that were found.

There were only three factors consistently found to have a prognostic effect on the risk of recurrence across all cohorts in which they were studied but all have somewhat limited clinical utility as a patient's history is unmodifiable and many studies have shown that it is not possible for all patients to remit to the point of being asymptomatic (e.g. Paykel, 2008). Factors such as cortisol response, rumination and experiential avoidance assessed here and others such as cognitive and information processing biases indicated as potentially prognostic for recurrence in Study 1, might have greater clinical utility as they are potentially modifiable; they might also have a mechanistic role in the action of the risk factors highlighted here. We could not have investigated such things from the articles reviewed in Studies 1 and 2 thus far. In order to broaden the consideration of the literature on the reported effects of potentially modifiable risk factors and reported mechanisms of action, we would need to consider a different but related literature. This is the focus for the next phase of the review, Study 3.

## 5 Study 3 – meta-review of non-systematic reviews

### 5.1 Aim

There was limited evidence derived from systematic reviews and cohort studies of potentially modifiable risk factors for recurrence of depression. However, a number of other potential risk factors were described that might be modifiable and merit further consideration. The aim therefore was to consider the literature on those potentially modifiable risk factors and consider mechanisms of action proposed in the literature. There were relatively few systematic reviews that focussed on identifying risk factors and mechanisms for recurrence but a large number of non-systematic reviews that did so but as they were non-systematic did not meet inclusion criteria for Study 1. So, in our third study, we undertook a meta-review of the non-systematic reviews reporting on risk factors for recurrence of depression or the mechanisms by which risk factors might have their effect.

### 5.2 Methods

We used the same searches and procedure for identification of studies conducted for Study 1 but applied a different set of inclusion and exclusion criteria as detailed in the General Methods (above).

#### 5.2.1 Quality ratings

Since they do not typically report any specific methods for conducting the review it is reasonable to assume that against any existing quality rating criteria for systematic reviews all would be judged as of very low quality.

### 5.3 Results

Twenty-seven non-systematic reviews were identified as meeting inclusion criteria and were included in the present study (see Supplementary Fig. 7). The primary studies included in each of the non-systematic reviews were checked against those reviewed in the systematic reviews in Study 1 to ensure that they were able to contribute knowledge beyond those findings. Of the 1598 studies included in all 27 non-systematic reviews, only 30 were included in reviews in both Study 1 and Study 3. None of the 27 non-systematic reviews included more than two articles reviewed in Study 1.

#### 5.3.1 Characteristics of the included reviews

Given the lack of a standardised format of reporting results for qualitative reviews data extracted from each study differed and determining whether or not the effects reported were prognostic or prescriptive (and if prescriptive what the interactions suggest) was not always possible (see Supplementary Table 8). All of the included studies reviewed clinical trials, cohort studies, experimental studies, or neuroimaging studies, with 15 of the 27 including more than one type of study. Several of the included studies also reviewed case-control studies, cross-sectional studies, quasi-experimental studies, or animal studies.

#### 5.3.2 Matrix of factors associated/not associated with relapse or recurrence

Details of the factors associated with recurrence in each of the included reviews along with any proposed mechanism of action can be found in Table 4. Many of the reviews reported on factors adding weight to some of the findings that had little or inconsistent evidence from Study 1. Risk factors associated with greater odds of recurrence were: the presence of cognitive biases (eight reviews); stressful life events (nine reviews); and rumination (six reviews). However, a number of factors not identified in Study 1 were also associated with an increased risk of recurrence: the presence of information processing biases (ten reviews); dysregulation of the hypothalamic pituitary adrenal (HPA) axis (five reviews); and dysregulation of rapid eye movement (REM) sleep (four reviews).

#### 5.3.3 Mechanisms of action

There were some common themes among the proposed mechanisms of action, including: the suggestion that cognitive biases were triggered by a change in mood (becoming dysphoric); that there is a learned association between the depressed state and the biases, as they occur together; and that biases may trigger rumination by the differential processing of negatively and positively valenced information, impacting the ability to recognise positive social cues, and privileging depressogenic attributions. Such processes might further engage depressive thinking that in turn might increase depressive symptoms and eventually trigger a recurrence. These processes are associated with limbic and neocortical reactivity to mood changes, especially dysregulation of the HPA axis and inflammatory responses that may act as diathesis for depressive onset, and are themselves associated with genetic alterations as more distal risk factors. Dysregulated REM sleep also interferes with the ability to process emotionally valenced information and regulate affect, and is particularly affected by alterations in a number of REM sleep related genetic mechanisms.

### 5.4 Discussion

The twenty-seven non-systematic reviews provided some continuity with what was found in Studies 1 and 2 above. The reviews point to stressful life events, cognitive and information processing biases, and ruminative thinking, in producing changes in mood. They also highlighted greater reactivity in the neocortical and limbic pathways and dysregulation of the HPA axis as more directly related to recurrence. There were suggestions that a number of genetic factors might act prognostically, potentially further “up-stream” by giving rise to an underlying diathesis for recurrent depression and potentially impacting upon other factors, including those related to hippocampal volume, brain maturation, REM sleep, and HPA axis dysregulation. There were also some potentially prescriptive effects highlighted including stressful life events, rumination, cognitive and information processing biases, and cognitive reactivity all of which might be more amenable to change with psychological therapies, whereas dysregulation of REM sleep might be more usefully treated with ADM. In addition, a prescriptive effective of childhood maltreatment such that treatment with MBCT lowered the elevated risk of recurrence relative to treatment with TAU. A number of genetic factors were highlighted as having potentially prescriptive effects, and mechanisms considered such that variants that lead to greater HPA axis dysregulation or REM sleep dysregulation might respond differentially to ADM compared to its absence in a manner not needed by low risk patients without such genetic variants. Given that the reviews were non-systematic, these indications must be treated with caution.

Some investigators have reconceptualised models of depression to consider relationships between cognitive and information processing biases and the onset of depressive symptoms that suggest mechanistic links between a number of the risk factors considered above (e.g. Disner, Beevers, Haigh, & Beck, 2011; Roiser, Elliot, & Sahakian, 2012). The “cognitive neuropsychological” model of depression (e.g. Roiser et al., 2012) proposes that negative affective biases result in changes in monoamine transmission and that this gives rise to negative belief systems that subsequently result in the signature features of a depressive episode: anhedonia and dysphoria (Roiser et al., 2012). Such biases rely on both “bottom-up processes” (triggered by emotionally salient stimuli like stressful life events), and “top-down processes” (cognitive mechanisms needed to inhibit reactions to emotionally salient but task-irrelevant information are sub-optimal in depression) (e.g. Castaneda et al., 2008; Roiser et al., 2012). These authors also suggested that evidence for the mechanisms proposed in the “cognitive neuropsychological model” of depression will be best delivered by neuroimaging studies or studies utilising an experimental paradigm to manipulate mood, cognitive processing, or information processing (Roiser et al., 2012). Given the findings of this meta-review, whereby potentially important risk factors and mechanisms for recurrence of depression have been alluded to but not assessed with sufficient depth in the literature, the final phase of this review focuses on reviewing these types of studies to better elucidate potential mechanisms of action.

## 6 Study 4 – systematic review of experimental & neuroimaging studies

### 6.1 Aim

The final study aimed to review neuroimaging and experimental studies to further investigate factors proposed to be associated with recurrence of depression in adults and to investigate potential mechanisms of action for those factors highlighted in Studies 1–3 above.

### 6.2 Methods

A systematic review was conducted of experimental and neuroimaging studies using the same search terms as for the previous three studies and including further bibliographic databases as detailed in the General Methods section above and in Table 1.

#### 6.2.1 Quality ratings

Study quality was assessed with criteria proposed by Kmet, Lee, and Cook (2004), using a 14-item rating scale where each item questions how the study was conducted or reported, e.g. “Design evident and appropriate to answer study question?” Each item has four possible responses “yes”, “partial”, “no”, or “n/a”; items are given a score of two if the answer is “yes”, one if the answer is “partial”, and zero if the answer is either “no” or “n/a”. A summary score is then calculated by summing the score from the 14 items and dividing it by 28 minus the number of not-applicable items multiplied by two, to derive a total score of between zero and one. Since no grading of study quality was suggested by the authors (Kmet et al., 2004), GRADE categories (Guyatt et al., 2008) were used as described above in Study 1. The following was used to guide judgements of overall study quality, studies scoring between: 0 and 0.25 were considered to be very low quality; 0.26 and 0.50 low quality; 0.51 and 0.75, or studies scoring above 0.75 but scoring 0 on the sample size item or not scoring 2 on items related to using appropriate outcome measures, conducting appropriate analyses, and supporting conclusions from the data, were considered moderate quality; studies that met these criteria and scored 0.76 or above they were high quality.

Information extracted on factors thought to predict depressive recurrence, along with any proposed mechanisms of action, was used to build a matrix as in Studies 1–3 above.

### 6.3 Results

Twenty studies met inclusion criteria and were reviewed (see Supplementary Fig. 8).

#### 6.3.1 Characteristics of the included studies

Fourteen of the twenty studies utilised experimental paradigms to manipulate cognitive processing or induce sad mood via inductions such as sombre music or tryptophan depletion. Three studies included only neuroimaging, five included neuroimaging and an experimental task such as the Stroop Colour-Word Task, and two were quasi-experimental with laboratory-based measurements of cognitive information processing but no experimental manipulation [e.g. Risch et al., 2010 used an Implicit Association Test to measure the association between implicit self-esteem and recurrent depressive episodes but did not manipulate self-esteem), see Supplementary Table 8 for details].

#### 6.3.2 Study quality

Nine of the included studies were judged to be of high quality, ten of moderate quality and one of low quality (see Supplementary Table 9).

#### 6.3.3 Factors associated with risk of relapse or recurrence

Several factors were associated with an increased risk of relapse or recurrence, with good evidence from high quality experimental or neuroimaging studies, although most of the studies investigated factors associated with recurrence only and not relapse. Five high quality and seven moderate quality studies found that cognitive and information-processing biases were associated with an increased risk of relapse (one study) and recurrence (11 studies). There was good evidence that cognitive reactivity is associated with an increased risk of recurrence from two high, three moderate, and one low quality study. One high and another moderate quality study found that HPA axis dysregulation was associated with an increased risk of recurrence. The neuroimaging studies reported good evidence of dysregulation in areas related to emotional or information processing and recurrence, in particular hypoactivity in neocortical and limbic areas (particularly the dmPFC and rACC) (Nixon et al., 2013), reduced hippocampal volume (Arnone et al., 2012; Kronmuller et al., 2008), and reduced grey matter volume in the right anterior cingulate and right inferior frontal gyrus (Serra-Blasco et al., 2016), see Table 5 and Supplementary Table 8 for details.

**Table 5:** Factors associated and not associated with relapse or recurrence to depression in adults reported in each of the included experimental and neuroimaging studies, and proposed mechanisms of action.

Study citation and quality rating	Factors investigated for association with relapse or recurrence to depression								Proposed mechanisms of action
	Stressful Life Events	Neuroendocrine /HPA Axis Dysregulation	Cognitive Reactivity	Information Processing Biases	Cognitive Biases	Rumination	Self-Esteem	Other	
Anderson et al., 2011 Moderate				++					Depression is associated with abnormalities in face emotion recognition that differ according to current mood state. The increased bias to recognise emotions in remitted depression is a possible mechanism of susceptibility to depression, although the effect was small.
Arnone et al., 2013 Moderate								Grey matter changes in hippocampi ++	Longer duration of untreated depressive symptoms before commencing treatment with antidepressants is associated with volumetric reduction in the hippocampus suggesting an ongoing toxic effect of the depressive state on hippocampal neurones or neurophil. Genetic factors may give rise to smaller hippocampi.
Chen et al., 2014 High				++	++				Taken together, the evidence suggests that N170 impairment during facial processing may underlie one of the hallmark features of depression - anhedonic symptoms. It may also lead to a better understanding of interpersonal difficulties that are related to depression, since patients utilize facial expressions as important indicators to manage their own behaviour and to evaluate the attitudes of others.
Chen et al., 2015 Moderate				++					Findings suggest that the recurrence of depressive episodes can lead to impaired pre-attentive information processing. This can then propagate a subsequent bottom-up orienting of attention to the deviant stimulus as the neurophysiological transmission travels from the MMN to the P3a.
Chopra et al., 2008 Moderate		++	+		++				Combination of high cortisol and three or more prior episodes increased risk of relapse. Cortisol and cognitive predictors of relapse were not associated with each other; they might represent separate vulnerabilities to relapse.
Dai & Feng, 2011 High				++					MDD and RMD participants showed deficient attentional inhibition of negative material.
Franck et al., 2007 High							++ Implicit ==Explicit	Severity of last episode ++	A higher implicit self-esteem reveals a vulnerability for depression
Huffziger & Kuehner, 2009 Moderate						++			Induced rumination maintained rather than exacerbated negative affect. Rumination may both prolong and exacerbate periods of negative mood.
Kronmuller et al., 2008 Moderate								Smaller left, right and total hippocampi in males +	It remains unclear whether smaller hippocampal volumes exist before the onset of depression or the reductions are the result of depression or other psychosocial influences.
Lethbridge & Allen, 2008 High	++		++					Dysfunctional thinking =	Change in dysfunctional thinking following sad mood induction did not emerge as a causal risk factor for depressive relapse. However, affective reactivity to the mood induction, specifically the degree of reduction in happy mood, emerged as an independent predictor of relapse. Life stress was also a strong and significant predictor of relapse.
Lythe et al., 2015 High						++		Connectivity of the RSATL with the SCSR during experience of self-blame ++	Finding supports the hypothesis that self-blame–selective changes in connectivity with the RSATL have a causal role in the pathophysiology of MDD but causality has not been proven as yet.
Moreno et al., 2000, Low			++					Double depression, duration of wellness after last episode and demographics, all = =	How a formerly depressed patient responds to a change in mood for the worse can determine whether or not they will relapse.
Morris et al., 2012, High	+	++						Cortisol reactivity to high stress ==	Cortisol reactivity to low stress predicted recurrence. Individual differences in HPA function during remission may serve as an indicator of vulnerability to recurrent depression.
Nixon, Liddle, Worwood, Liotti, & Nixon, 2013, High	+			++	++			Hypoactivity in right dmPFC and right ACC ++	The importance of dmPFC and rACC processing within the time frame utilised in the present study may lie in early, adaptive reappraisal of negative experiences, and a failure to do this may lead to vulnerability to depressive relapse.
O'Brien-Simpson et al., 2009 High								Attenuated startle response ++	Inhibited baseline startle may be a reliable endophenotypic marker of biological predisposition to recurrent depression
Risch et al., 2010 Moderate							++ Implicit		None stated
Segal et al., 1999 Moderate			++		++				None stated
Segal et al., 2006 High			++						A mild negative mood experienced by someone with a history of depression, can trigger some of the cognitive features of a depressed episode. The presence of such reactivity in recovered patients signals a residual but heightened risk for recurrence.
Serra-Blasco et al., 2016 High								Grey matter volumes of right inferior frontal gyrus +++ & Anterior Cingulate +++	Grey matter volumes in combination with duration of depressive illness and number of prior MDEs can improve predictive ability of models predicting clinical outcomes combining recurrence and chronic course.
Watkins & Baracaia, 2002 Moderate	+		+	++		++		Psychosocial impairment post MDE ++	One possible mechanism of relapse in recovered depressed patients is state-oriented ruminative responses to a stressful life events leading to impaired social problem solving and further escalation of the stressful situation.

Key: ++ good evidence of positive association with relapse or recurrence; + some evidence/suggestion of positive association with relapse or recurrence.

= = good evidence of no association with relapse or recurrence; = some evidence/suggestion of no association with relapse or recurrence.

#### 6.3.4 Mechanisms of action

Specific mechanisms of action were proposed in five studies (Chen et al., 2014; Chen et al., 2015; Lythe et al., 2015; Nixon et al., 2013; Segal et al., 2006). Although the authors of the remaining studies did not propose specific mechanisms there was some consistency across the studies. Taken together the reviewed studies suggested a role for dysfunction in the neural networks responsible for information processing or affective and cognitive processing in response to stress. The results of some studies suggested this might be influenced by dysfunction in the HPA axis or other inflammatory responses, and changes in mood that prevent appropriate reappraisals of negative experiences. Other studies found that rumination prolonged negative mood and prevented utilising social cues and social support, thus conferring greater risk of recurrence.

### 6.4 Discussion

The finding that cognitive and information processing biases are associated with an increased risk of recurrence from Studies 1, 2 and 3 above was further supported by the findings from the 20 studies in the present review. HPA axis dysfunction, stressful life events, and greater affective reactivity were also associated with an increased risk of recurrence. There was evidence of an increased risk of recurrence with hypoactivity and morphological alterations in neocortical and limbic regions and the proposed mechanism suggesting that these neurological factors are associated with the cognitive and information processing biases which themselves impact the ability to manage stress or changes in mood, therefore providing support for the mechanisms alluded to in Study 3 above.

## 7 General discussion

This series of reviews found strong evidence for three factors associated with an increased risk of relapse and recurrence in depression: a history of childhood maltreatment; residual depressive symptoms at the end of treatment; and a history of recurrence. There was little evidence that risk increased in a linear fashion as a function of the number of prior episodes. Moreover, residual symptoms and duration of recovery both play a moderating role such that a history of recurrence is mostly prognostic only among patients in full remission (patients with residual symptoms are at elevated risk regardless) and patients who sustain recovery for five or more years move to low risk status. Younger age of first onset, greater severity of the index episode and high neuroticism appear to be prognostic of recurrence but were not universally found to be so in the literature. There was good evidence that having a comorbid anxiety disorder is prognostic for risk and the same is true for high levels of comorbid anxiety symptoms even sans diagnosis. Both rumination and cognitive biases were prognostic for subsequent recurrence, as were affective and information processing biases; attentional and cognitive control; cognitive and affective reactivity; dysregulation of REM sleep; dysregulation of certain neuroendocrine functions and dysfunction and morphological changes in associated neocortical and limbic regions. Some studies reported that the duration of the index episode was associated with greater risk of recurrence but others found no such effect. There was very limited and inconsistent evidence that family history of depression is prognostic of recurrence. Only one of the nearly seventy studies we reviewed looked at risk for relapse and recurrence separately. This was both surprising and disconcerting given the importance placed on the distinction and the implications that it has for treatment.

There was some evidence that childhood maltreatment, residual symptoms, and a history of recurrence all moderated the effects of treatment relative to controls, usually by virtue of reducing risk for those at higher risk on these factors if allocated to an active treatment group, down to a similar level to those low on the factors regardless of treatment. Whether they moderate differential response among different specific active treatments remains to be seen. A number of other factors were found to be associated with an increased risk of recurrence as moderators of the effects of psychological, somatic or pharmacological treatments, these include rumination, cognitive and information processing biases, cognitive reactivity, and dysregulation of REM sleep in general; interpersonal stress in psychosocial relapse prevention treatments; medication resistance and DST reactivity for those treated with ECT; and comorbid anxiety symptoms, somatic pain, and a shorter time in response for those discontinuing ADM. On the basis of these findings we developed a conceptual framework that can be utilised to guide future research into relapse and recurrence.

### 7.1 Interpretation

**Fig. 2:**
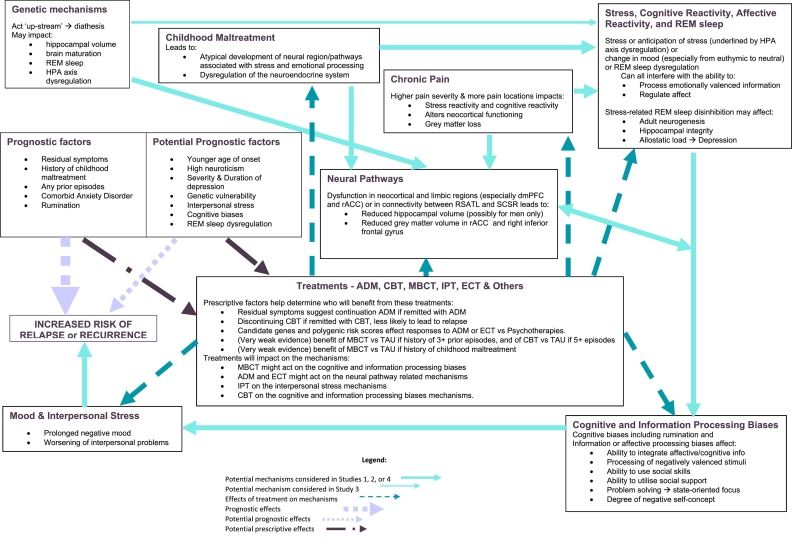
Conceptual Framework: Prognostic and prescriptive factors and their interaction with mechanisms of relapse and recurrence of depression in adults.

That residual symptoms and a history of recurrence predict subsequent risk fits with the “consensus view” of recurrence, but the inclusion of childhood maltreatment is something new and suggests the operation of novel mechanisms, discussed below and illustrated in Fig. 2. The evidence was not conclusive that each successive episode was associated with an increment in risk although there were several mitigating factors that might explain this deficit. The biggest problem is that few studies reported risk as a function of number of prior episodes but instead dichotomized at some point along the continuum in a manner that makes it hard to test. Examination of the data from the one study that did examine risk as a function of specific number of episodes showed that there is an incremental effect, that is largely monotonic in nature but of decreasing magnitude. Residual symptoms and duration of recovery both moderated that relationship such that a history of recurrence was only prognostic among those in full remission and less than five years in recovery. It is also possible that perceived or actual risk drove more and longer treatment in a fashion that could have masked such a relationship.

Causal processes unfold over time and some of our prognostic indices may be more distal and others more proximal to the actual recurrence. If this is the case, more proximal “down-stream” factors like residual symptoms might be better predictors of subsequent recurrence than more distal “up-stream” factors such as a history of recurrence, this might explain why the strength of the effect for previous episodes was found to be less than that for residual symptoms. It is also possible that all three factors are proxies for another underlying factor such as a neurobiological diathesis for recurrence, and so although the number of previous episodes has been considered to have a linear association with recurrence this may not best reflect the nature of any such underlying diathesis. If there is such a diathesis it would seem likely that it effects whether depressed patients end up with ruminative thinking patterns, information processing and cognitive biases that give rise to a negative self-concept once in recovery, as these appear to be related to a greater risk of recurrence. Negative self-concept has itself been related to multiple previous episodes (e.g. Elgersma, Glashouwer, Bockting, Penninx, & de Jong, 2013). It also is possible that the risk of recurrence related to having previous episodes is not static over time, and that the cohorts that have found evidence of an effect of multiple compared to just one previous episode on recurrence might have captured different points in time when the risk may be different. Considering the results of the National Comorbidity Survey the 12-month prevalence of MDD decreased with increasing age (Kessler et al., 2003), and when examining non-clinical samples from childhood onwards in the Oregon Adolescent Depression Project there were more first onsets at ages 13–18 than in adulthood and a peak of recurrences in the 18–24 year old group which had lessened in the 24–30 year old group (Rohde, Lewinsohn, Klein, Seeley, & Gau, 2013). The cohort followed in the CDS was about half a decade younger than the cohorts followed in the other studies (GHC, NEMESIS, NESARC and NESDA) and it is possible then that multiple previous episodes is a better predictor of subsequent recurrence for younger adults that no longer remains predictive if they survive without recurrences into middle adulthood. This would be consistent with the limited evidence from Study 2 that younger age of onset is predictive of a marginal increase in the speed of recurrence. We are not able to determine which if any of these reasons is correct, but given the “consensus view” that the number of previous episodes is important over and above simply having a history of recurrence, it would have implications for the timing and optimal amount of treatment. It is worth investigating whether increased risk is associated with the number of episodes (if it is, it might either be because simply going through a depressive episode increases subsequent risk or because risk remains static, but high risk individuals have more episodes), particularly when controlling for some of the issues that may have biased or confounded the studies reporting this effect (discussed in detail in above).

Also of note was the inconsistent evidence for an association between neuroticism and increased risk of relapse or recurrence, which although not part of the “consensus view” in the same way as history of recurrence, has been reported to increase the risk of recurrence in many studies in the past (e.g. Bos, Bouhuys, Geerts, Van Os, & Ormel, 2007). Overall, across the four studies of this review it would appear as though there is support for neuroticism as a prognostic indicator of recurrence but the inconsistency in the findings is somewhat surprising; why it should be associated with a greater odds of recurrence but not a greater hazard of recurrence is puzzling and not explicable by any mechanism considered in this review. It is more likely that this is an artifact of the different measures of neuroticism and different samples used in the studies that reported different indices (hazards and odds of recurrence). In the two studies from the same cohort (discussed in Study 1) the null finding regarding the hazard of recurrence was close to reaching significance despite using a smaller subset than used in the study that reported the odds of recurrence by neuroticism.

In Studies 1 and 2 we found good evidence that a recent history of comorbid anxiety disorders or elevated symptoms of anxiety, and rumination are also prognostic indicators of recurrence. Several of the factors found to be associated with higher risk of recurrence in Studies 1 and 2 have yet to be replicated. These include chronic pain and multiple physical symptoms, cognitive biases, cognitive reactivity to stress or changes in mood, rumination, interpersonal stress, and a higher cortisol awakening response. Further, Study 1 highlighted a number of prescriptive factors that modified the risk of recurrence relative to the effects of certain treatments among patients that have residual symptoms the treatment used to achieve partial remission matters; discontinuing CBT may not lead to the same increased risk of recurrence as discontinuing ADM does if the patient has residual symptoms. In addition in two small trials of MBCT a history of three or more previous episodes was prescriptive such that those with this history did better with MBCT than they did with ADM, and in one trial of CBT this pattern occurred for those with five or more compared to less than five previous episodes. However, as something cannot be prescriptive without being prognostic at least in one of the treatment groups, and as these findings were not supported by much of the rest of the reviewed literature this suggested prescriptive effect is worth further consideration. It could be due to chance, a number of biases in those studies reporting the effect or a number of potentially alternative explanations, or it could point to a “critical-mass” of previous episodes rather than a linear increase in risk with each additional episode that could potentially be in keeping with the findings of Study 2 here. However, we could not determine which if any of these applies. There was also the suggestion of a prescriptive effect of childhood maltreatment with less bad outcomes for those with such histories in MBCT relative to TAU. Study 3 offered further support to these factors and highlighted several others such as: genetic vulnerabilities particularly relating to neurogenesis and REM sleep; information processing biases; inflammatory responses/markers particularly dysregulation of the HPA axis and C-reactive protein levels; and REM sleep dysregulation. However, the studies reviewed were of very low quality by virtue of being non-systematic and so their conclusions were considered with caution. Study 4 showed that there is evidence for the associations between the cognitive, information processing and affective biases, and recurrence of depression considered in Studies 1, 2 and 3. In addition it suggested that these biases may be associated with dysregulation of neuroendocrine/inflammatory responses such as the HPA axis, reduced neuroplasticity and volumetric changes particularly in limbic areas, and dysregulation of the neural systems responsible for information processing and emotional processing (recognising emotions from facial expressions).

Studies 3 and 4 investigated mechanisms concerning the nature of the relationships between risk factors and recurrence. These were considered all together and used to inform the development of the conceptual framework of relapse and recurrence of depression shown in Fig. 2. Risk factors with strong evidence from Studies 1 and 2 were given greater importance in the model than those identified in Studies 3 and 4 due to the higher quality of evidence from these types of studies (Coleman et al., 2005). Potential mechanisms illustrated in the conceptual framework were taken from related themes of proposed mechanisms in Studies 3 and 4, and were expressed in the model more directly if the mechanism was explicitly tested in studies reviewed in Study 4, rather than mechanisms proposed or alluded to but without being directly tested in that way in Study 3.

The conceptual framework points to a focus in several domains: 1) What population we might study to better understand prognostic indices of recurrence of depression and the prescriptive steps that can be taken to reduce that risk; 2) How research efforts might better elucidate the mechanisms driving recurrence and therefore how to potentially prevent these outcomes; 3) How clinicians might modify their treatments in lieu of more accurate prognostic indicators of recurrence; and 4) How treatments might interact with risk factors and mechanisms to effect risk for recurrence.

#### 7.1.1 Clinical implications

From the conceptual framework clinicians might consider keeping patients in treatment longer in an attempt to get them to the point of full remission (minimal residual symptoms); targeting efforts at prevention to those not able to achieve full remission, those with a history of childhood maltreatment, and those with a history of any prior recurrences. In addition, longer and additional types of treatment might be offered to those whose first depressive onset occurred at a younger age, those with problems with rumination, those with a recent history of or current comorbid anxiety disorders or high levels of anxiety symptoms, and those with REM sleep dysregulation, with the latter two indicating the consideration of ADM. The findings of these reviews suggest that residual depressive symptoms might be a prescriptive factor, modifying the effects of treatments with CBT or continuation ADM relative to ADM withdrawal. This raises the question of whether combination therapy for those not treated to complete remission particularly adding psychological therapy after completing a course of ADM, might help protect against or delay a recurrence. This has been trialled successfully by adding CBT as ADMs are discontinued (Guidi et al., 2015) and could be considered as a particularly protective option given the suggestion that long term ADM use can in some patients become a contributing factor in increasing risk for recurrence (Fava, 2003). These findings underscore the importance of assessing for a history of childhood maltreatment and adding adjunctive psychological interventions specifically targeting its residual effects. Such interventions might take the form of compassion-focussed work to target shame-based difficulties and self-blame, cognitive behavioural interventions targeting low self-esteem, trauma focussed interventions (if appropriate), or interpersonal interventions targeted at relational difficulties. Further questions raised by this review centre around whether adding interventions targeted at chronic pain, rumination, worry, cognitive reactivity, interpersonal difficulties, sleep hygiene, and cognitive and attentional control could also help reduce the risk of recurrence. Finally, we would encourage clinicians to assume that the number of prior episodes is a useful index of future risk; the existing literature is not as conclusive as we expected it to be, but that is more a lack of supportive evidence than any clear indication that it is not so. It may be that a history of recurrence or family history of depression are less important than presence of residual symptoms or a history of childhood trauma or it could be that the impact of all these factors is summative and need to be considered.

#### 7.1.2 Research implications

From the conceptual framework we can speculate that those with: residual symptoms post-treatment; a history of childhood maltreatment; a history of recurrence, comorbid or recent anxiety disorders; or problems with rumination might be studied to enrich our understanding of the mechanisms underpinning relapse and recurrence. In particular, greater focus might be given to investigating effects of childhood maltreatment on the neocortical and limbic pathways that control processing of affective information or cognitive control, and outcomes of acute phase treatment for depression. Efforts to further develop our understanding of the mechanisms underlying relapse and recurrence might include tests of information processing, cognitive and affective reactivity to stress or changes in mood and they might include biological tests of inflammatory markers such as HPA axis regulation, C-reactive protein expression or cortisol upon waking. Further, in neuroimaging studies they might particularly focus on neocortical and limbic regions of interest involved in functions such as those above and those involved in attentional or cognitive control. There are useful examples of how future studies might be set up to best investigate these factors. For example Lythe et al. (2015) included functional neuroimaging of people with remitted depression, recurrent depression and stable depression, and had 14 months of follow-up to capture recurrences. Another example is Watkins and Baracaia (2002) who used a cross-sectional comparison having randomly allocated currently depressed, recovered depressed and never depressed people to their experimental paradigm and controlled for treatment. In addition, the conceptual framework suggests that as the identified mechanisms become better elucidated, they become targets for intervention.

Finally, to better consider the effect of increasing episodes, neuroticism, age of onset, and duration and severity of depression on risk for recurrence the best way to investigate this would be with a large-scale prospective cohort study that: i) samples populations prior to first onset depression or which seeks to confirm past experiences of depression with corroborating evidence such as medical records or confirmed treatment history not just retrospective self-reports, and if sampling populations with depression at baseline the study should stratify to ensure representative samples from community, primary, secondary/specialist and inpatient care settings; ii) contains a long-term follow-up; iii) charts the course of symptom changes over the full follow-up period (with the CIDI or LIFE interviews with life-chart analyses for example), with clinical events confirmed by appropriate clinicians or by corroborating evidence from medical records, so capturing all possible relapses and recurrences; iv) records all depression treatments over follow-up either directly or through data linkage with health records or national health surveillance systems or registers; and v) uses propensity score matching or other methods to control for some of the selection biases involved in treatment (who is treated, with what, at what dose, and how long for). It would also be helpful if cohorts were developed outside the USA or the Netherlands and included ethnically diverse populations representative of the population of adults with depression at large.

### 7.2 Limitations

There are several limitations to the approach taken in this paper. Study 1 was limited by a paucity of systematic reviews of risk factors of relapse and recurrence to depression, and in particular systematic reviews that had a stated aim to identify or consider the relative strengths of different risk factors. The reviews included in Study 1 were equally limited by a paucity of primary studies investigating risk factors as their primary objective. The included reviews that attempted to do this were of low or very low quality and so the results from these reviews were necessarily treated with caution. Nonetheless, we were able to support the “consensus view” (e.g. Campbell, 2009; Lin et al., 1998; Paykel & Priest, 1992) with regard to residual symptoms, and to a lesser degree than expected with regards to a history or previous episodes, and propose the addition of childhood maltreatment.

Study 2 was limited by the small number of cohorts using continuous measurement of symptoms over follow-up and robust measures of confirming relapses or recurrences, so all the reviewed studies were from cohorts in the USA or the Netherlands. We limited our inclusion criteria to studies of adults only; coupled with the insistence on the continuous measurement of symptoms over follow-up, this led to the exclusion of a number of big cohort studies. Were our inclusion criteria less strict in this regard we would have been better placed to investigate the effects of increasing numbers of episodes, age at baseline, and age of onset on the risk for recurrence.

Studies 1 and 3 were limited by the lack of reporting on the quality of the studies reviewed in each reviewed article. While we judged the quality of the reviews, it may be the case that some of the lower quality reviews included high quality primary studies and therefore the findings from these may have effectively been “downgraded” by the quality criteria being applied at the level of the review and not at the level of the included studies. Further, the reviews included in Studies 1 and 3 did not include a number of studies relevant to the present review. As a part of the systematic review process, a large number of cohort studies (not meeting inclusion criteria for Study 2), RCTs and case control studies were identified that reported on risk factors for relapse or recurrence but these studies were not included in the reviews which made up Studies 1 and 3. That these studies were not included does not mean that they were “missed”, instead they may just not have met the inclusion criteria of those reviews given that the aims of those reviews were generally not focussed on specifically identifying risk factors for relapse and recurrence of depression. While there were a number of areas of agreement and themes coming out of our qualitative syntheses of the four phases of this article, we were not able to combine results across our four studies and were only able to conduct quantitative synthesise on the data from the cohorts reviewed in Study 2. The “box score” type method used to develop the matrices in each of the four studies did not allow for sophisticated statistical testing or adjustments for potential sources of bias, so we were unable to consider the impact of publication bias on the results reported.

In addition, the individual studies included in the reviews in Studies 1 and 3 primarily used the Frank et al. (1991) definitions of relapse and recurrence, but this may have led to a degree of bias in the results as they tend to compare people who do not relapse or experience recurrence over the study period with people that do relapse or recur, without separating out those that have had multiple previous episodes from those that have had their first lifetime episode. It is therefore likely that they under-sampled those who only have a single lifetime episode (Monroe & Harkness, 2011).

Finally, despite relatively longstanding data on cognitive and information processing biases and their association with relapse and recurrence, there are significant difficulties in translating knowledge of these risk factors to the consulting room. Assessing for these biases in brief and non-invasive ways requires technologies not available to the majority of clinicians. Studies attempting to address this particular issue by using wearable technology are being set up (e.g. Remote Assessment of Disease and Relapse – Central Nervous System: “Innovative Medicines Initiative”, n. d.), though it is too early to determine whether or not they are indeed helpful to this end.

## 8 Conclusion

Taken together these four reviews suggest that: 1) childhood maltreatment, residual symptoms, history of recurrence, a history or current comorbid anxiety disorders, and rumination are prognostic indicators of recurrence; 2) these may also all be prescriptive risk factors too but the evidence for each is limited; 3) higher neuroticism and earlier age of onset appear to be prognostic risk factors but were less well supported by the literature; 4) longer duration and greater severity of depression may be prognostic risk factors but there was a lack of agreement in the reviewed literature; 5) there are a number of factors that have not previously been systematically reviewed that may be targeted in psychological interventions (information processing and cognitive biases, reactions to stress or changes in mood, attentional or cognitive control) or pharmacological interventions (comorbid anxiety symptoms, and REM sleep dysregulation) aimed at prevention. The four reviews have identified several potential mechanisms for the action of such risk factors and have proposed potential inter-relationships between these factors to form a conceptual framework that may help guide clinicians and future research into relapse and recurrence of depression.

## Role of funding sources

At the time of writing Dr. Joshua E J Buckman was on a Fellowship funded by the Wellcome Trust, Grant reference 20,129/Z/16/Z. Dr. Alan Underwood was seconded to UCL as a Clinical Tutor in the Research Department of Clinical Educational and Health Psychology. Professors Pasco Fearon and Stephen Pilling, Ms. Katherine Clarke and Dr. Rob Saunders were all funded by University College London through the Research Department of Clinical, Educational and Health Psychology and respectively funded by the Higher Education Funding Council for England and the British Psychological Society. Professor Steven Hollon was on faculty in the Department of Psychology at Vanderbilt University in Nashville Tennessee USA.

None of these funders had any role in the study design, collection, analysis or interpretation of the data, writing the manuscript, or the decision to submit the paper for publication.

## Contributors

JB and SP conceived the original idea for this review with support from PF. JB and AU developed the framework and procedures for the four linked studies with support from SP and SH. JB conducted the reviews with AU co-reviewing and co-rating study quality, with SP providing support where any differences in determinations of inclusion/exclusion or study quality existed between JB and AU. KC and RS helped JB prepare the data and conduct the meta-analyses. JB prepared the manuscript with repeated revisions commented on and amended by AU, KC, RS, PF, SH and SP.

## Conflicts of interest

None.

## Appendix A Supplementary data

Bibliographic database searches and results. Image 1

Additional details extracted from each reviewed study. Image 2

Supplementary Data Tables and Figures. Image 3
