# Supplementary material for: Risk factors for relapse and recurrence of depression in adults and how they operate: A four-phase systematic review and meta-synthesis
Source: Clin Psychol Rev. 2018 Aug;64:13–38. doi: 10.1016/j.cpr.2018.07.005 (PMC6237833; doi:10.1016/j.cpr.2018.07.005)
Supplement: Supplementary file 1 — Bibliographic database searches and results. [file mmc1.pdf]

Appendix A

Bibliographic database searches and results

Searches	Results
**CAB Global Health Archive 1910 to 1972**	
1. (depression or Depressive disorder or Major depression or Unipolar depression or MDD).mp.	3255
2. (risk adj5 relapse).mp.	47
3. 1 and 2	1
4. (bipolar or bipolar disorder or manic depression).mp.	145
5.(alcohol abuse or drug abuse).mp.	359
6. 4 or 5	504
7. 3 not 6	**1**
**Cochrane CENTRAL Trial Register (searched on 8th May 2017)**	
1. ("Depression" or "MDD" or "Unipolar" or "Depressive"):ti,ab,kw (Word variations have been searched)	46057
2. ("relapse" or "Recurrence" or "remission"):ti,ab,kw (Word variations have been searched)	56043
3. ("Bipolar" or "schizophrenia" or "psychosis" or "schizoaffective"):ti,ab,kw (Word variations have been searched)	16778
4. #1 and #2	4510
5. #4 not #3	**3784**
**Cochrane Database of Reviews (searched 8th May 2017)**	
1. ("Depression" or "MDD" or "Unipolar" or "Depressive"):ti,ab,kw (Word variations have been searched)	1550
2. ("relapse" or "Recurrence" or "remission"):ti,ab,kw (Word variations have been searched)	54235
3. ("Bipolar" or "schizophrenia" or "psychosis" or "schizoaffective"):ti,ab,kw (Word variations have been searched)	17866
4. #1 and #2	128
5. #4 not #3	**105**
**Embase 1947 to 2017 Week 19**	
1. exp recurrent brief depression/ or exp major depression/ or exp depression/ or exp long term depression/	390578
2. (depression or Depressive disorder or Major depression or Unipolar depression or MDD).mp.	546774
3. 1 or 2	577642
4. relapse.mp.	198614
5. recurrence.mp.	429938
6. 4 or 5	577352
7. 3 and 6	15951
8. (risk adj5 relapse).mp.	21582
9. 8 and 3	1661
10. ((relapse or recurrence) adj5 depressi*).mp.	2731
11. 9 or 10	3956
12. bipolar.mp.	92009
13. bipolar disorder/ or bipolar mania/ or bipolar depression/ or bipolar II disorder/ or bipolar I disorder/	50743
14. exp schizoaffective psychosis/	8257
15. alcohol abuse/	24774
16. drug abuse/	48482

17. 12 or 13 or 14 or 15 or 16	124307
18. 11 not 17	3149
19. limit 18 to (human and english language and journal)	**2617**

Scoping Search: Embase 1947 to 2013 Week 47 Search for Review Articles Only

1. exp recurrent brief depression/ or exp major depression/ or exp depression/ or exp long term depression/	323279
2. (depression or Depressive disorder or Major depression or Unipolar depression or MDD).mp.	460246
3. 1 or 2	486582
4. relapse.mp.	154579
5. recurrence.mp.	338066
6. 4 or 5	461682
7. 3 and 6	12839
8. (risk adj5 relapse).mp.	19560
9. 8 and 3	1444
10. ((relapse or recurrence) adj5 depressi*).mp.	4673
11. 9 or 10	5379
12. bipolar.mp.	77167
13. bipolar disorder/ or bipolar mania/ or bipolar depression/ or bipolar II disorder/ or bipolar I disorder/	40107
14. exp schizoaffective psychosis/	6134
15. alcohol abuse/	19526
16. drug abuse/	43593
17. 12 or 13 or 14 or 15 or 16	103069
18. 11 not 17	1068
19. ((risk or odds or chance or probability) adj5 relapse).mp.	20885
20. 3 and 19	1538
21. 11 or 20	5417
22. 21 not 17	1150
23. systematic review.mp. or "systematic review"/	98424
24. meta analysis.mp.	115569
25. literature review.mp.	55934
26. 23 or 24 or 5	221228
27. 22 and 26	**77**

International Pharmaceutical Abstracts 1970 to April 2017

1. (depression or Depressive disorder or Major depression or Unipolar depression or MDD).mp.	9262
2. (risk adj5 relapse).mp.	328
3. 1 and 2	51
4. (bipolar or bipolar disorder or manic depression).mp.	1663
5. (alcohol abuse or drug abuse).mp.	7457
6. (schizophrenia or psychosis or schizoaffective).mp.	4614
7. 4 or 5 or 6	13057
8. 3 not 7	40
9. limit 8 to (English language and human)	**39**

Ovid MEDLINE 1946 to May Week 1 2017

1. exp major depression/ or exp "depression (emotion)"/	97036
2. exp Depressive Disorder, Major/	25059
3. exp Depressive Disorder, Major/ or exp Depressive Disorder/ or exp Depression/	185779

4. 1 or 2 or 3	186094
5. ((relapse or recurrence) adj5 depressi*).mp.	1748
6. bipolar disorder/ or affective psychosis/ or mania/	36514
7. exp Schizoaffective Disorder/	47821
8. Substance-Related Disorders/	88464
9. Schizophrenia/	93120
10. 6 or 7 or 8 or 9	157171
11. 4 not 10	166993
12. Child/	1549731
13. 11 not 12	154096
14. 13 and 5	1378
15. limit 14 to (English language and humans and journal article)	**1221**

Scoping search: Ovid MEDLINE 1946 to November Week 3 2013 Search for Review Articles Only

1. exp major depression/ or exp "depression (emotion)"/	73699
2. ((relapse or recurrence) adj5 depressi*).mp.	1406
3. bipolar disorder/ or affective psychosis/ or mania/	30705
4. exp Schizoaffective Disorder/	37751
5. 3 or 4	65458
6. exp Depressive Disorder, Major/	18234
7. exp Depressive Disorder, Major/ or exp Depressive Disorder/ or exp Depression/	148814
8. 2 or 7	149074
9. Bipolar Disorder/	30705
10. 8 not 9	139693
11. Child/	1339552
12. 10 not 11	129087
13. 2 and 7	1146
14. 13 not 19	1054
15. 14 not 11	1001
16. Substance-Related Disorders/	76481
17. 15 not 16	990
18. Systematic review.mp	37815
19. literature review.mp	38952
20. meta analysis.mp	67893
21. 18 or 19 or 20	127903
22. 17 and 21	**33**

PsycEXTRA 1908 to May 8, 2017

1. exp Postpartum Depression/ or exp Depp Recurrent Depression/ or exp Atypical Depression/ or exp Endogenous Depression/ or exp Reactive Depression/ or exp Treatment Resistant Depression/ or exp Major Depression/	4554
2. (risk adj5 relapse).mp.	61
3. (risk adj5 recurren*).mp.	65
4. 2 or 3	125
5. 1 and 4	11
6. exp Bipolar Disorder/ or exp Mania/	681
7. exp Schizoaffective Disorder/ or exp Schizophrenia/ or exp Psychosis/	1600
8. mental health/	6344
9. exp Alcohol Abuse/	3193
10. exp Drug Abuse/	11241
11. 6 or 7 or 8 or 9 or 10	19292
12. 5 not 11	11
13. limit 12 to (human and English language)	**10**

Prospero (searched on 8th May 2017)

("Depression" OR "Depressive" OR "MDD" OR "Unipolar") AND (published):RS	**123**

PsycINFO 1806 to May Week 1 2017

1. exp major depression/ or exp "depression (emotion)"/	134770
2. recurrent depression/	727
3. exp Relapse Prevention/ or exp "Relapse (Disorders)"/	8179
4. 1 or 2 or 3	141654
5. ((relapse or recurrence) adj5 depressi*).mp.	2317
6. 4 and 5	2010
7. bipolar disorder/ or affective psychosis/ or mania/	27009
8. exp Schizoaffective Disorder/	2856
9. exp schizophrenia/	82005
10. exp psychosis/	104723
11. 7 or 8 or 9 or 10	126783
12. exp Alcohol Abuse/	44337
13. exp Drug Abuse/	100937
14. 11 or 12 or 13	223192
15. exp Child Psychopathology/	2329
16. 14 or 15	225277
17. 6 not 16	1700
18. Limit 17 to (human and English language and “0100 journal”)	**1367**

Scoping Search: PsycINFO Search for Review Articles Only

exp major depression/ or exp "depression (emotion)"/	110021
(depression or Depressive disorder or Major depression or Unipolar depression or MDD).mp.	213339
recurrent depression/	613
1 or 2 or 3	213628
relapse.mp.	17745
recurrence.mp.	5591
exp Relapse Prevention/ or exp "Relapse (Disorders)"/	6898
5 or 6 or 7	22074
4 and 8	5177
exp "Literature Review"/ or exp Meta Analysis/	25128
systematic review.mp. [mp=title, abstract, heading word, table of contents, key concepts, original title, tests & measures]	8537
meta analysis.mp. [mp=title, abstract, heading word, table of contents, key concepts, original title, tests & measures]	14296
10 or 11 or 12	42663
9 and 13	192
exp Bipolar Disorder/	18849
exp Alcohol Abuse/	38107
exp Drug Abuse/	85184
exp Drug Addiction/	11194
15 or 16 or 17 or 18	103406
14 not 19	161
((relapse or recurrence) adj5 depressi*).mp.	1920
20 and 21	**52**
