# Supplementary material for: Risk factors for relapse and recurrence of depression in adults and how they operate: A four-phase systematic review and meta-synthesis
Source: Clin Psychol Rev. 2018 Aug;64:13–38. doi: 10.1016/j.cpr.2018.07.005 (PMC6237833; doi:10.1016/j.cpr.2018.07.005)
Supplement: Supplementary file 2 — Additional details extracted from each reviewed study. [file mmc2.pdf]

Appendix B

Additional details extracted from each reviewed study

Study 1:

Reviewed Studies	Databases searched and years included	Search terms	Inclusion Criteria	Exclusion Criteria
Beshai et al., 2011	PsycINFO and PubMed years not stated	keywords only: "relapse", "recurrence", "prevention", "depression", "cognition", "mindfulness", "interpersonal" AND "therapy"	a) adult participants (18 or over), b) employing some form of psychotherapy, c) psychotherapy used as a stand alone procedure targeting relapse/recurrence prevention d) used either a treatment or non-treatment control group	None Stated
Berwian et al., 2016	Pubmed	"(depress* OR MDD*) AND (relapse* OR recurren*) AND (predict* OR risk) AND (discontinu* OR withdraw* OR maintenance OR maintain or continu*) AND (antidepress* OR medication OR drug)	a) patient population aged between 18 and 65; b) main diagnosis of Major Depressive Disorder (MDD); c) remitted from a depressive episode while treated with antidepressant medication; d) follow-up of at least 6 months to assess relapse; e) part of the sample discontinued the ADM (discontinuation could either be part of a randomized controlled design, whereby part of the sample received placebo after randomization starting at a predefined point in time or based on the decision of the patient and treating physician as part of a naturalistic design); f) reported relapse predictors either in the discontinuation group alone; or reported interaction of treatment x relapse predictors.	a) anonymous data derived from health systems prescription records; b) confounds with psychotherapy.
Bourgon & Kellner, 2000	Medline and hand searching Also searching paper journals prior to 1966	Keywords: "electroconvulsive therapy" AND textwords: "relapse" or "recurrence"	None Stated	None Stated
Clarke et al., 2015	CENTRAL, Embase, Medline, PsycINFO and PROQUEST from beginning of databases to 2012	Keywords and MeSH terms: depression' and 'long-term/ relapse/recurrence' using a highly sensitive RCT filter	Randomised controlled trials of non-pharmacological interventions were included if participants were adults who had experienced an episode of major depression and partially or fully recovered. There was a minimum follow-up period of 12-months from the start of the intervention.	None Stated
Feng et al., 2012	Cochrane Library, EBSCO Host, Medline OVID, ProQuest Medical Library, and PubMed, and hand searching of references. All searched 2000-2010.	Keyword and MeSH terms: group therapy, cognitive therapy, cognitive behavioral therapy, CBGT, psychotherapy, depression, relapse, and recurrence	Subjects were diagnosed with depression using the following standards: International Classification of Diseases (ICD) 10, Diagnostic and Statistical Manual of Mental Disorders (DSM) III, DSM-IIIR, DSM-IV, DSM-IV-TR or Research Diagnostic Criteria (RDC). Subjects were over 18 years of age and could be any gender or race; (2) The experiment was a randomized controlled trial; (3) At least one group underwent CBGT; (4) At least one control group was included; (5) The study was written in English; (6) The results were presented in terms of the level of depression and relapse rate of depression; and (7) The study had sufficient data (e.g., sample size, percentage, t-value, F value, p value, average, standard deviation, and changes in variables) to calculate the effect size.	if the CBGT took place over the phone or the Internet.
Gueorguieva et al., 2017	Pubmed	("depression" OR "major depressive disorder") AND "discontinuation" AND "trial" in any field	Studies of adults 18 years or older, with a HAM-D score of at least 16 at baseline, trials of either Fluoxetine or Duloxetine vs placebo.	None stated at level of the review, details of exclusion criteria are given for each of the reviewed studies

Hardeveld et al., 2010	PsycINFO and Medline, plus hand searching of references. All searched January 1980-August 2008	Keywords only: 'Recur*', 'Relaps*', 'Depress*', 'Predict*' and 'course'.	English Language, adult population only. Then: naturalistic cohort study, including subjects with MDD, MDD diagnosed by interview of checklist based on RDC/BCC, DSM-III/III-R/IV or ICD-9/10; course measured by standardised instrument/checklist, minimum f/u of 6 months, at least 50 ps, criteria of remission/recovery/relapse/recurrence according to Frank et al 91 criteria.	Bi-polar, SAD, Post-partum depression, studies including specific age groups only.
Hughes & Cohen, 2009	Medline, PscyINFO, Embase, Cochrane Library, Hand searching of references of included studies. Searched 1988-2008	Keyword and MeSH terms: Given in tables for each database	We searched for reports published since 1988 in English. Other inclusion criteria were: 1) follow-up period of at least10 years including at least 1 follow-up assessment post-1988, 2) adult (over age 18) sample, no minimum size, 3) at least 1 group (in comparative studies) or subset of participants identified as having a unipolar depressive disorder, 4) a statement or description that participants received AD treatment (no minimum duration or number treated), and 5) at least 1 clinical or psychosocial outcome, excluding suicide or mortality, measured or assessed. We used the same inclusion criteria for studies of nondrug treated individuals, except for requiring a statement that participants had not received ADs, and accepting studies conducted before 1988 because explicitly drug-free samples were more likely to have been assembled prior to that year.	We excluded studies that examined only suicide or mortality because the extensive literature on these outcomes deserves its own analysis
Kok et al., 2013	PubMed, Embase, PsycINFO. Searched from beginning of databases to 4th December 2012.	Keyword and MeSH terms: ‘depression or depressive disorder or major depression were combined with heart diseases or gastrointestinal diseases or diabetes mellitus or arthritis, rheumatoid or asthma or HIV or neoplasms and incidence or follow-up studies or prognos* or predict* or course or outcome or relaps* or recur* or remis* or epidemiology.	In english, dutch, spanish, polish or german. (1) longitudinal measurement of the course of depression (2) providing absolute numbers or percentages of recurrence a) diagnosis established with an interview based on state- of the- art depression criteria (e.g. Diagnostic and Statistical Manual of Mental Disorders, DSM-III/III-R/DSM-IV) [12–14] or b) with standardized questionnaires that assess depressive symptoms (e.g., Inventory of Depressive Symptomatology, IDS) [15] or -, Hamilton Rating Scale for Depression, HRSD) [16] (3) with a follow-up of at least six months (4) in which data were collected for patients with and without a certain co-morbid chronic somatic illness at the same measurement intervals a) where co-morbid chronic somatic illnesses were assessed either via self-report or b) medical records or c) by a (1) longitudinal measurement of the course of depression (2) providing absolute numbers or percentages of recurrence a) diagnosis established with an interview based on state- of the- art depression criteria (e.g. Diagnostic and Statistical Manual of Mental Disorders, DSM-III/ III-R/DSM-IV) [12–14] or a) with standardized questionnaires that assess depressive symptoms (e.g., Inventory of Depressive Symptomatology, IDS) [15] or -, Hamilton Rating Scale for Depression, HRSD) [16] (3) with a follow-up of at least six months (4) in which data were collected for patients with and without a certain co-morbid chronic somatic illness at the same measurement intervals a) where co-morbid chronic somatic illnesses were assessed either via self-report or b) medical records or c) by a diagnosis of a medical professional.	Bi-polar disorder
Nanni et al., 2012	Medline, PsycINFO, and Embase. Searched from beginning of the databases to December 2010	Keywords only: child* maltreatment, child* abuse, child* neglect, early experience) and relevant depression measures (search terms: depress*, mood disorder, MDD, recurrence, persistence, chronic, duration, length, improvement, response, remission, treatment, psychotherapy, CBT, pharmacotherapy, antidepressant, SSRI)	Definition of childhood adversities consistent with childhood maltreatment physical abuse, sexual abuse, neglect, or family conflict or violence); diagnosis of depressive disorder, ascertained either in population-based or in clinical samples; and evaluation of relevant depression measures (12). human subjects, written in English.	Animal studies; articles not published in english

Study 4: Additional Details extracted from each reviewed neuroimaging and experimental study

Reviewed Studies	Inclusion Criteria	Exclusion Criteria	How Sample Recruited
Anderson et al., 2011	aged 18–60 years and in good physical health. Control group participants were required to be without a personal history of psychiatric disorder or a family history of treated depression; currently depressed participants needed to meet criteria for a current major depressive episode; and those in the remitted depressed group were required to be in full remission with no other current psychiatric disorder, a history of at least two prior major depressive episodes (one lasting at least 2 months) and a Montgomery–A˚ sberg Depression Rating Scale (MADRS) score of less than 13	current or past physical illness or debilitating physical condition; current alcohol or drug dependence or harmful use; a history of bipolar affective disorder, psychosis, dementia or mental impairment; and taking medication that might interfere with neuropsychological function (apart from antidepressants, antipsychotics or lithium in the case of patients with depression). Depressed participants could meet criteria for a comorbid anxiety disorder provided this had not pre-dated the primary diagnosis of major depressive disorder.	From Larger sample of 2004 Primary Care Patients in 2 UK centres supplemented with psychiatric outpatients with history of depression
Arnone et al., 2013	For currently depressed group: Medication free; DSM-IV diagnosis of MDD, MADRS score of 20+ . For Remitted Group: At least one past episode of MDD and score of less than 10 on MADRS.	Current comorbid Axis 1 Psychiatric Disorder and primary cluster A or B Axis II disorders were excluded. Any unstable medical condition, neurological disorders, history of significant head trauma, lifetime history of substance or alcohol misue disorders, and contraindication ot MRI scanning, and postiive family history for Psychiatric Disorders if due to be included in healthy control group.	Recruited at University of Manchester. No mention of method of doing so.
Chen et al., 2014	Chinese Han, Right Handed,	Not Stated	From inpatient wards at Center for Mental Disease Control and Prevention of Baoji Third Hospital of the People's Liberation Army in China
Chen et al., 2015	Chinese Han, Right Handed,	Not Stated	From inpatient wards at Center for Mental Disease Control and Prevention of Baoji Third Hospital of the People's Liberation Army in China
Chopra et al., 2008	Not Stated	Not Stated	The mood challenge was conducted as part of a larger study to examine cognitive predictors of depressive relapse (Segal et al., 2006). In brief, in Phase 1 of this study, 301 patients with MDD were randomized to receive either CBT or antidepressant therapy. In Phase 2 of the study, subjects who remitted from depression underwent a mood provocation protocol and were followed for 18 months.
Dai & Feng, 2011	All had to be Chinese-speaking adults aged 18-40 years old. For NC participants: score of 4 or below on the BDI and BAI, and no history of depressive disorder or any other psychological disturbance, according to the DSM-IV (SCID). For Remitted Depressed Group (RMD): scoring 7 or below on HRSD and at least two previous depressive episodes with the most recent onset at least 8 weeks ago. MDD Group: scoring 20 or higher on the HRSD and diagnosis of MDD by a psychiatrist according to the DSM-IV criteria.	Severe head trauma and learning disabilities, current or lifetime psychotic symptoms, bipolar disorder and alcohol or substance abuse within the past 6 months. All participants had normal or rectified eyesight with no colour blindness, and all were right-handed.	NC Group: Participants recruited through advertising on university bulletin boards. RMD Group: recruited through psychotherapists, and were screened to determine whether they had fully recovered from depression, according to the DSM-IV criteria. MDD Group: recruited through psychiatrists

Franck et al., 2007	for currently depressed group: DSM-IV diagnosis of MDD, HRSD score of 13+ and BDI score 20+. For Formerly Depressed: past diagnosis of MDD given by a psychiatrist or clinical psychologist, had received pharmacotherapy or psychotherapy during the episodes, and symptom free for at least one month. In addition HRSD score below 13 and a BDI-II total score lower than 20. The never depressed: were screened with the M.I.N.I. and were included if they reported no prior history of depression. HRSD score lower than 13 and a BDI-II-NL total score below 20.	For Never Depressed: any past psychological disorder based on self-report questionnaire.	Currently depressed individuals (N= 28, CD) were recruited from different psychiatric hospitals in Belgium. The formerly depressed and never depressed controls were recruited using media advertisements in Belgium. All participants were financially compensated for their participation in the study.
Huffziger & Kuehner, 2009	Diagnostic inclusion criteria were Major Depression, single (F32) or recurrent (F33) episode, and Dysthymic Disorder (F34) according to ICD-10 (WHO,1992) at index admission	Not stated	Within the context of a 3.5 year follow-up assessment of a larger longitudinal study, we investigated 76 depressed patients originally recruited during their inpatient treatment at the Central Institute of Mental Health in Mannheim, Germany
Kronmuller et al., 2008	Not Explicitly Stated	Not Explicitly Stated	in-patients with DSM–IV major depression treated in the Department of Psychiatry of the University in Heidelberg were recruited
Lethbridge & Allen, 2008	Meeting DSM-IV-TR criteria for at least one previous episode of MDD.	Current depression at time of first testing session or during 10week period prior to that. History of psychosis or bi-polar disorder. Inability to speak English, and/or inability to provide informed consent.	recruited from the University of Melbourne and from readers of the Herald Sun, the largest circulating Melbourne newspaper. Participants were recruited via research advertisements placed around the University of Melbourne campus and in the post-graduate email newsletter and from the general community via an editorial in the 'Health Watch' section of the newspaper.
Lythe et al., 2015	In MDD Group was MDD according to DSM-IV-TR in remission for at least 6 months. At least two months duration of one past major depressive episode, a past moderate or severe depressive episode according to the International Classification of Diseases. Both Groups had to be Psychotropic medication free, and right handed, native English speaking, with normal vision or vision corrected to normal.	For MDD Group: current Axis 1 disorders including history of substance or alcohol abuse and past comorbid Axis 1 disorders being likely cause of depressive symptoms.<br>For HC: Current or past Axis 1 disorders, first-degree family history of MDD Bipolar or Schizophrenia. MRI contraindications, psychotropic medication, psychotherapy whilst taking part in the study, significant psychosocial impairment as an indicator of a possible personality disorder (assessed on the Global Assessment of Functioning scale (GAF)2), a Montgomery Åsberg Depression Rating Scale3 (MADRS) score of > 10, current self-harming behaviour, clinically relevant MRI abnormalities, developmental disorders, learning disabilities, an Addenbrooke's Cognitive Exam-R score < 88 (completed in participants over 50 years of age4), neurological illness, or physical illnesses that significantly alter brain function or blood flow.	Participants were recruited using online and print advertisements as part of the UK Medical Research Council funded "Development of Cognitive and Imaging Biomarkers Predicting Risk of Self-Blaming Bias and Recurrence in Major Depression" project.
Moreno et al., 2000	Not Stated	Not Stated	The screening methods, and demographic and clinical characteristics of the subjects have been described in a previous report detailing the acute mood response to TRP depletion (Moreno et al 1999).
Morris et al., 2012	Inclusion in Remitted Depressed group required past diagnosis of MDD according to DSM-IV criteria assessed with the SCID-I.	Current MDD, current or past bi-polar disorder or PTSD or health conditions known to influence HPA function (e.g. Cushing's disease, hyperthyroidisim, diabetes) and non-prescription drugs that might affect cortisol levels.	Recruited from graduate and undergraduate programs at Vanderbilt University. All participants were part of a larger study examining cortisol reactivity to a psychosocial stress or in remitted depressed and never depressed young adults.
Nixon et al., 2013	Minimum inclusion for the patient group required at least two previous episodes of DSM-IV major depression, diagnosed through the SCID, now in sustained remission/ recovery (with normalized function for at least 3 months and a 17-item HAMD score <8).	co-morbid Axis I psychiatric disorder (with particular care to exclude bipolar II disorder and anxiety disorders); personality disorder; drug/alcohol disorder; untreated medical disorder; any previous or current central nervous system (CNS) disease; or fMRI safety issues. In addition to these criteria, potential<br>controls were excluded if there was evidence of current or past psychiatric disorder.	Patients were recruited through physician referral from general adult psychiatric clinics in Nottingham, UK. Controls were mainly recruited through posters displayed at a General Hospital and a community surgery in Nottingham.

O'brien-Simpson et al., 2009	All participants had experienced a previous case level episode of MDD, but did not meet criteria at the time of testing.	none of the participants selected for the study met criteria for drug or alcohol abuse or dependence. Additionally, none of the participants had a history of brain injury or central nervous system disease.	Not stated
Risch et al., 2010	Patients were included if they met DSM-IV criteria for major depressive disorder or recurrent major depressive disorder, currently in remission.	Depressive disorder with psychotic symptoms, bipolar disorder, posttraumatic stress disorder, organic psychiatric disorders, substance-abuse related disorders, schizophrenia, schizoaffective disorders, and borderline personality disorder. Patients with acute suicidality were also excluded. RD patients were excluded if they scored higher than 18 on the Beck Depression Inventory (BDI). ND individuals were recruited through advertisement and were matched to the total patient group with respect to age, sex, and education. ND individual were included after having been screened for the absence of depressive disorders using the IDCL and provided that they had no prior history of depression.	CD, RCD, and RD patients were recruited from the outpatient psychiatric clinics of the Friedrich Schiller University of Jena and the Sophien and Hufeland Clinic of Weimar, Germany.
Segal et al., 1999	All participants were between the ages of 18 and 65 years old, met minimum eighth-grade education requirement or were able to complete assessment instruments unassisted, and spoke English as their primary language. Only chose if their most recent episode of depression met criteria for a primary diagnosis of MDD.	a) Current diagnosis of bipolar affective disorder, substance abuse disorder, or schizophrenia, and b) a trial of ECT within the past 6 months. `	From one of two outpatient clinics offered through the Clarke Institute of Psychiatry's Mood and Anxiety Disorder Division and the Depression Clinic.
Segal et al., 2006	Inclusion criteria were diagnosis of major depressive disorder according to DSM-IV criteria, aged between 18 and 65 years, minimum eighth-grade education, and ability to read English and to provide informed consent. Posttreatment evaluations were based on DSM-IV criteria derived from the Longitudinal Interval Follow-Up Evaluation (LIFE) interview and the 17-item Hamilton Depression Rating Scale (HDRS-17). Following the consensus recommendations, 20 remission was defined as the patient reporting minimal symptoms for a minimum of 12 weeks, no longer meeting diagnostic criteria for major depressive episode (MDE), and a HDRS-17 score of less than or equal to 10. These criteria were used to determine which patients would be eligible to participate in the mood provocation phase of the study.	(1) a current diagnosis of bipolar disorder, substance abuse disorder, schizophrenia, or borderline personality disorder, (2) a trial of electroconvulsive therapy within the past 6 months, and (3) a score of less than 12 on the Hamilton Depression Rating Scale.	Subjects were recruited through clinical referrals from the Mood and Anxiety Disorders Program at the Centre for Addiction and Mental Health or from media announcements.
Serra-Blasco et al., 2016	Right Handed, with MDD, willing to undergo MRI scan.	Participation in a Deep Brain Stimulation study	Recruited from the Psychiatric Department of the Hospital de la Santa Creu I Sant Pau in Barcelona, Spain.
Watkins & Baracaia, 2002	Aged 18-65 years old. Currently depressed had to meet criteria for MDD on SCID and score 16+ on the BDI. Recovered Depressed did not meet criteria for a current MDE on SCID but had at least 1 previous episode, scored 14 or less on BDI. Never Depressed did not meet criteria for any past or current diagnosis of MDE on the SCID and scored 14 or less on the BDI	None stated	Currently depressed were recruited from both inpatient and outpatient settings. Recovered Depressed were recruited from a self-help charity for depression. Never Depressed group were recruited but the authors did not state how.
