# Supplementary material for: Risk factors for relapse and recurrence of depression in adults and how they operate: A four-phase systematic review and meta-synthesis
Source: Clin Psychol Rev. 2018 Aug;64:13–38. doi: 10.1016/j.cpr.2018.07.005 (PMC6237833; doi:10.1016/j.cpr.2018.07.005)
Supplement: Supplementary file 3 — Supplementary Data Tables and Figures. [file mmc3.pdf]

Supplementary Data Tables and Figures

Figure 1 illustrates the difference between prognostic and prescriptive effects. In each instance the control group is depicted in blue with a dotted-line and the treatment group in red with a solid line. The left panel depicts an instance in which there is a main effect for the prognostic index (patients who are high on the risk factor are 25% more likely to relapse than those who are low on the risk factor) and a main effect for treatment (those who do not get treated are 25% more likely to relapse than those who do get treated). Note that there is no suggestion of a patient-by-treatment interaction and thus the prognostic factor is not prescriptive. The right panel depicts an ordinal interaction in which patients at low risk do not benefit from treatment whereas those at high risk do (by 25%). Note too that the risk factor is prognostic in the control condition but not in the treatment condition; in effect, treatment off-sets the consequences of whatever mechanisms put some people at greater risk. This is a common pattern described in the acute treatment literature in which efficacious treatments have their greater differential effects on those who are more severe such that they start out worse but end treatment no worse off than those initially less severe (e.g. Dimidjian et al., 2006; Elkin et al., 1989). Disordinal interactions (not depicted) involve situations in which some patients do better on one treatment whereas other patients do better on the other, such that a plot of differential response takes the shape of an “X”. Such disordinal interactions are rare when a treatment is compared to a control since it is unlikely that anyone would actually do worse on the latter, but they are more common when two or more efficacious interventions are compared that likely operate through different causal mechanisms. For example, in one study patients with personality disorders were more likely to respond to ADM than to CBT and those without showed the opposite pattern (Fournier et al., 2008).

Supplementary Figure 1. Risk indices can be prognostic or prescriptive: Prognostic indices are best studied in longitudinal designs with minimal treatment. Prescriptive effects require treatment interventions.

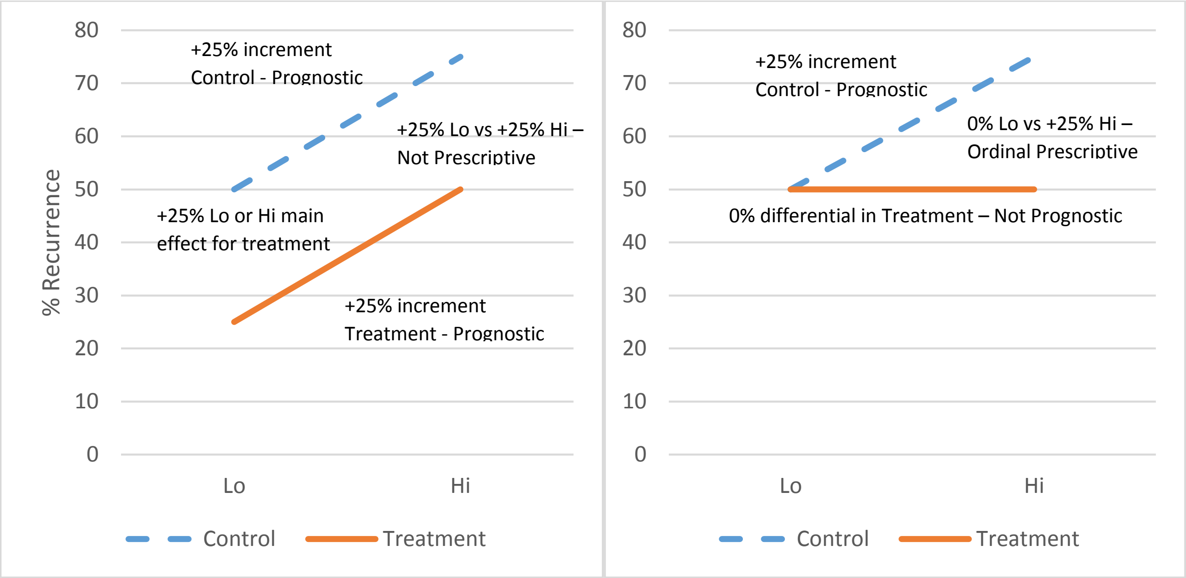

Note: Lo & Hi represent categories of a given risk factor for recurrence

Supplementary Figure 2. Flow diagram of study selection for Study 1.

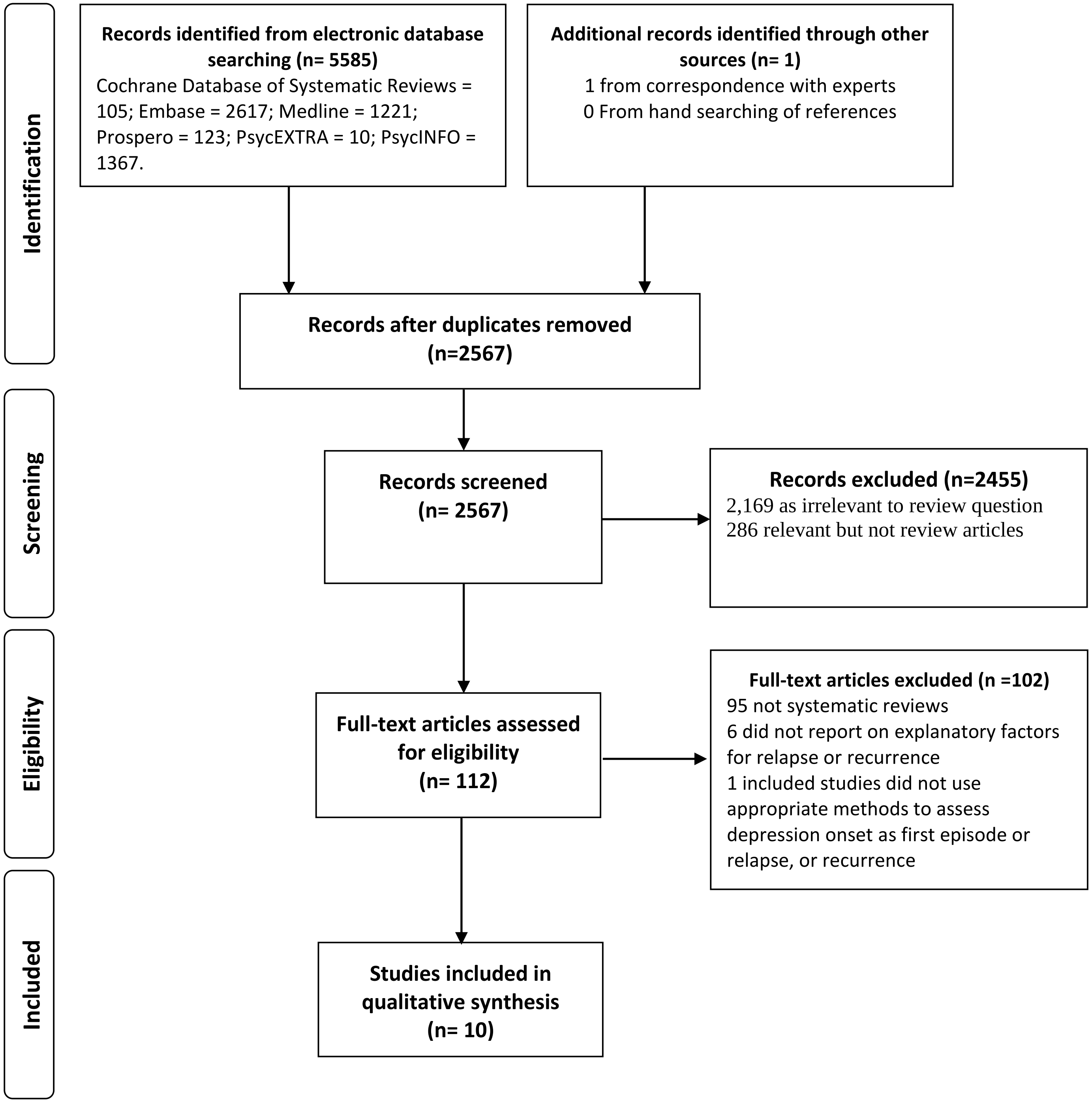
Identification
Records identified from electronic database searching (n= 5585)
Cochrane Database of Systematic Reviews = 105; Embase = 2617; Medline = 1221; Prospero = 123; PsycEXTRA = 10; PsycINFO = 1367.
Additional records identified through other sources (n= 1)
1 from correspondence with experts
0 From hand searching of references
Records after duplicates removed (n=2567)
Screening
Records screened (n= 2567)
Records excluded (n=2455)
2,169 as irrelevant to review question
286 relevant but not review articles
Eligibility
Full-text articles assessed for eligibility (n= 112)
Full-text articles excluded (n =102)
95 not systematic reviews
6 did not report on explanatory factors for relapse or recurrence
1 included studies did not use appropriate methods to assess depression onset as first episode or relapse, or recurrence
Included
Studies included in qualitative synthesis (n= 10)

Supplementary Table 1. Data extracted from included systematic reviews.

Context of findings	Review	Number of included studies and study designs	Populations	Definitions of relapse and recurrence used	Interventions	Comparisons of relevance and what results relate to	Main results relevant to relapse and recurrence
**Closest to the "state of nature", purely prognostic**	Hardeveld et al., 2010	27 naturalistic cohort studies, N=4286	Adults MDD	Frank et al. (1991)	N/A	Non-recurred within settings; rates compared across settings.<br><br>**Mostly recurrence**.	Recurrence rates were high in specialty care (60% after 5 years, 67% after 10 years, and 85% after 15 years from the Collaborative Depression Study (CDS)) and markedly lower in single studies from primary care (31% at one year) and in the general population (35% at 15 years). Residual symptoms and the number of previous depressive episodes were reported to be the strongest prognostic indicators of recurrence. Neuroticism and psychosocial difficulties were considered prognostic factors but were rarely studied. Baseline severity was considered a risk factor but there was some disagreement in the reviewed literature. There was disagreement in the reviewed literature regarding the prognostic effects of younger age of initial onset, longer duration of depression, a family history of depression, and having psychiatric comorbidities. The authors suggested that poor "coping skills" are also associated with greater odds of recurrence, this was reported from two cohorts one which assessed self-efficacy and another that assessed self-esteem and mastery. Demographic indices did not predict recurrence.
	Nanni et al., 2012	16 Epidemiological studies: 9 Population health surveys with prospective follow-up, 7 clinical sample surveys with prospective follow-up, combined N=23,544. 10 Clinical Trials (2 controlled observational studies; 6 RCTs; 2 non-randomised controlled trial) combined N = 3,098.	Adults 18+ in Epidemiological studies (except one study 15+), Adolescents and adults of all ages in RCTs.	Frank et al. (1991)	In Clinical Trials - CBT or Anti-depressants	Recurred Vs non-recurred; Duration of episode. **Combined relapse and recurrence**.	Meta-analysis of the 16 epidemiological studies found an increase in odds of recurrence for those that were maltreated in childhood compared to those that were not OR(95%CI) = 2.27(1.80-2.87), confounding by age of initial onset was reported to be unlikely. All but one of the primary studies found this effect (combined n=6838), and in the one that did not (n=3353) there was a trend towards an effect for experiencing a frightening event before the age of 17 [risk ratio(95%CI) = 1.27(0.98-1.61)]. In addition, a meta-analysis of 10 RCTs to assess the effects of childhood maltreatment on treatment outcomes found increased odds of lack of clinical improvement in those with a history of childhood trauma or maltreatment OR(95%CI) = 1.40(1.19-1.66). The latter was not significant for psychotherapy 1.12(0.68-1.85) but was for both pharmacotherapy 1.26(1.01-1.56) and combined treatment 1.90(1.40-2.58). The latter contrasts were prognostic only.
	Hughes & Cohen, 2009	15 long-term prospective cohort studies with at least 8 years of follow-up (27 publications), n=5061	Adults 18+ with depression, dysthymic disorder or mood disorder	Rush et al. (2006)	Long-term ADM in 24 studies, no-ADM treatment in 3 studies.	Recurred Vs non-recurred. **Recurrence only**.	Three of the cohorts were also included in the Hardeveld et al. (2010) review. Recurrence rates ranged from 40% to 85% (the latter the 15-year rate in the CDS), with approximately 25% achieving recovery or clinical response and another quarter evidencing systematically poor outcomes. Long-term outcomes were generally poor with little difference in the treated and untreated samples. The heterogeneity of treatment received across the follow-ups and the lack of relation to subsequent recurrence makes it difficult to draw any firm conclusions, the authors suggested that there was inconsistent evidence for episode duration and symptom severity as predictors of recurrence.

	Kok et al., 2013	3 Prospective cohort studies (1 of children and adolescents (n=54)), 1 retrospective and prospective cohort study. N for studies of adults = 1956	Adults with MDD and Chronic Somatic Illness (3 studies) children (aged 8-13) with MDD and CSI (1 study)	Frank et al. (1991)	No specific intervention.	Adults with MDD without Chronic Somatic Illness in three of the four studies, children (aged 8-13) with MDD without CSI in the other. **Combined relapse and recurrence**.	No indication that comorbid somatic illness was associated with a greater prognostic risk for relapse or recurrence. Risk ratios in the three studies of adults at two or three year follow-up ranged between RR(95%CI)= 0.94(0.65-1.36) and 1.37(0.78-2.41). The authors did not consider other prognostic effects.
**Prognostic in treated samples ignoring treatment allocation**	Gueorguieva et al., 2017	4 RCTs, N=1462	Adults in remission on continuation phase ADM at baseline.	Not stated	Discontinuation after Duloxetine or Fluoxetine vs placebo	Return of clinical level of symptoms Vs no return of symptoms. **Combined relapse and recurrence**.	After discontinuation of ADM prognostic effects across treatment conditions leading to an increased likelihood of relapse were found for shorter periods between randomisation and scoring below 10 on the HRSD per 1-week OR(95%CI)=1.10(1.06-1.15), higher levels of residual symptoms (OR(95%CI)=1.28(1.01-1.62)) and female sex (OR(95%CI)=1.56(1.23-2.06)). There was no prognostic effect of previous episodes on likelihood of relapse, either 1 or 2 vs 0 OR(95%CI) =1.33(0.78-2.28), 3 or 4 vs 0 OR(95%CI)=1.06(0.61-1.87), or 5+ vs 0 OR(95%CI) =1.34(0.78-.230). Calculating the effect of any compared to 0 prior episodes OR(95%CI) = 1.06(0.71-1.59), and if including all those with missing data in the group that did have prior episodes as a sensitivity analysis OR(95%CI) = 1.16(0.78-1.72). There was no prognostic effect of age either at baseline or age of onset on likelihood of relapse. Being on active drug vs placebo was associated with approximately 13% difference in the likelihood of relapse.
	Clarke et al., 2015	29 RCTs. 22 included in meta-analysis combined N= 4216	Adults with at least one past episode of MDD who had partially or fully recovered	any kind of significant deterioration preceded by clear improvement	Post-remission relapse prevention psychotherapy	Between groups, relapsed Vs non-relapsed. **Combined relapses and recurrence**.	The authors noted that the presence of residual symptoms and a history of multiple previous episodes worsened prognosis in some studies but were not able to assess the risk factors themselves due to the absence of consistent summary statistics across the reviewed studies, so comparison between trials could not be made.
**Prognostic apparently ignoring treatment allocation, some prescriptive effects between treatment groups**	Feng et al., 2012	32 RCTs. 19 included in meta-analysis, combined N=2152	Adults 18+ with MDD	Not stated.	Group CBT	Relapsed vs Non-relapsed. **Combined relapse and recurrence**	It is not particularly clear what these authors found as they reviewed studies of individual CBT, MBCT, IPT, behavioural activation (BA), and positive psychology and considered these all under the term "CBT group therapy", they examined both remission and relapse and found an effect of residual symptoms increasing the risk of relapse or recurrence, this appeared to be prognostic (irrespective of treatment condition), they suggested that it also has a prescriptive effect for group CBT vs other assessed interventions but the effect size was small and there were a number of studies that did not find the effect. Past depressive episodes, experience of the therapist, type of control group, group size, group manual, therapy frequency, and take-home assignments showed no significant effects. It is likely that these were assessed prescriptively as the authors considered the variables as moderators of the effect of "CBT group treatment".

	Beshai et al., 2011	20 RCTs, 4 non-randomised controlled trials, n=2370	Adults with current depression	Frank et al. (1991)	Any psychological relapse prevention therapy	Relapsed Vs recovered/remitted patients in different intervention groups of trials. **Relapse and recurrence separately.**	A prognostic effect of residual symptoms within ADM and CBT treatment conditions was reported, and a potential prescriptive effect for CBT (but some contradictory evidence) and for ADM but not for combined ADM-CBT vs ADM alone. Some suggestion that residual symptoms may be a prognostic factor for relapse but not recurrence. In one study chronic interpersonal stress (as opposed to cognitive or personality factors) was the only prognostic factor significant for depressive relapse, also in one study avoidant coping style and day-to-day stress were both prognostically and prescriptively related to worse odds of relapse (they interacted with previous episodes and treatment allocation such that patients with avoidant coping styles and more prior episodes were more likely to relapse after CBT than TAU, the 3-way interaction reduced the effect of avoidant coping on recurrence in the TAU group). Rumination was considered prognostic and there was no effect of the content of negative thoughts. Several reviewed studies found no difference in rate of relapse or recurrence for those with a history of three or more compared to those with less than three previous episodes. Others found a clear prognostic effect within treatment conditions, and a clear prescriptive effect such that 3+ episodes was related to higher rates of recurrence in TAU (as was 5+ in another study) but not in MBCT or in CBT.
	Bourgon & Kellner, 2000	44 RCTs and Pseudo-RCTs, n=1895 (adults; excluding numbers from studies of adolescents and geriatric populations only).	Adults with current severe depression	Frank et al. (1991)	ECT	Relapsed vs Non-relapsed. **Mostly relapse alone.**	Six of nine studies of the dexamethasone suppression test and one study of cortisol hyper-secretion showed a prescriptive effect for ECT patients such that post-ECT non-suppressors were at higher risk of relapse relative to those treated with ADM. Studies of the thyrotropin-releasing hormone stimulation test and shortened rapid eye movement sleep latency were inconclusive. Although there was some evidence that delusions worsen prognosis and that it has a prescriptive effect such that ECT patients with delusions had lower rates of recurrence compared to ADM-continuation without ECT, in one study, these effects were not confirmed in other studies. Medication resistance before ECT was the only other patient characteristic shown to have a prognostic effect among ECT treated patients.
**Purely prescriptive between treatment conditions**	Berwian et al., 2017	13 RCTs, n=2558	Adults aged 18-65 treated with ADM	Frank et al. (1991)	Discontinuation of ADM	Relapsed vs Non-relapsed. **Relapse only**	No prescriptive effect of multiple previous episodes, episode duration, chronicity, age of onset, severity of depression at baseline, and demographics (including age, gender, ethnicity, and race). Residual symptoms were found not to have a prescriptive effect on risk of relapse following ADM discontinuation in most reviewed studies though there was a trend in one study. Neurovegetative and melancholic symptoms did not interact significantly with treatment response, neither did double-depression or history of hypomanic symptoms. Some evidence of a prescriptive effect of high comorbid anxiety, with lower rates of recurrence for those continuing ADM vs discontinuing, likewise for somatic pain.

Abbreviations: BA – behavioural activation; BDI – Beck Depression Inventory; CBT; cognitive behaviour therapy; CI – confidence interval; CSI –chronic somatic illness; DSM-IV/DSM-III – Diagnostic and Statistical Manual of Mental Disorders 4th/3rd Edition; ECT – Electroconvulsive Therapy; HAM-D – Hamilton depression rating scale; HRSD - Hamilton rating scale for depression; ICD-10 – International Classification of Diseases 10th Edition; IPT - Interpersonal psychotherapy; MADRS Montgomery-Asberg Depression Rating Scale; MBCT –Mindfulness based cognitive therapy; MDD – major depressive disorder; MDE – major depressive episode; RCT – randomised controlled trial; RDC – research diagnostic criteria; SCID – Structured Clinical Interview for DSM-IV; TAU – treatment as usual

Supplementary Table 2. Rating of study quality for included systematic reviews using AMSTAR and items from PRISMA and the Cochrane Reviewer's Handbook.

Study Citation	Research question and inclusion criteria established prior to review?	Were there at least 2 independent data extractors and a consensus procedure for disagreements?	2+ databases searched? Keywords/MeSH terms stated, searches supplemented by hand searching?	Was publication status used as an inclusion criteria?	List of included and excluded studies provided?	Characteristics of included studies provided?	Scientific quality of included studies assessed and documented?	Was scientific quality of included studies used appropriately in formulating conclusions?	Were the methods used to combine findings of studies appropriate?	Was the likelihood of publication bias assessed?	Was the conflict of interest stated? - both in the review and in the included studies	*At least cursory reasons given for exclusion of studies **	*Investigators blinded to participant allocation in included clinical trials#*	*Fully assessed and dealt with sources of bias#*	*Overall Quality Rating*
Beshai et al., 2011	Yes	Can't answer	No	Can't answer	No	No	No	N/A	N/A	No	No	No	No	No	Very low
Berwian et al., 2017	Yes	Yes	No	No	No	Yes	No	N/A	Yes	No	Yes	No	Yes	No	Moderate
Bourgon & Kellner, 2000	Yes	Can't answer	No	Can't answer	No	No	No	N/A	N/A	No	No	No	No	No	Very low
Clarke et al., 2015	Yes	Yes	Yes	Yes	Yes	Yes	Yes	Yes	Yes	Yes	Yes	Yes	Yes	Yes	High
Feng et al., 2012	Yes	Yes	Yes	Yes	No	Yes	Yes	Yes	Yes	Yes	No	Yes	Yes	No	High
Gueorguieva et al., 2017	Yes	Can't answer	No	No	No	Yes	No	N/A	Yes	No	No	Yes	No	No	Low
Hardeveld et al., 2010	Yes	Yes	Yes	Yes	No	Yes	No	N/A	N/A	No	Yes	Yes	N/A	No	Low
Hughes & Cohen, 2009	Yes	No	Yes	Yes	No	Yes	No	N/A	N/A	No	Can't answer	Yes	N/A	No	Low
Kok et al., 2013	Yes	Yes	Yes	Can't answer	No	Yes	Yes	No	Can't answer	No	No	Yes	N/A	No	Moderate
Nanni et al., 2012	Yes	Yes	Yes	Can't answer	No	Yes	No	Yes	Yes	Yes	Yes	Yes	No	Yes	High

All items are from AMSTAR unless otherwise stated; [*]From PRISMA; [#] From Cochrane Reviewer's Handbook.

Supplementary Figure 3. Percentage recurrence by treatment group in three RCTs, showing that **n**umber of prior episodes is both prognostic (in TAU) and prescriptive (in both MBCT and CBT)

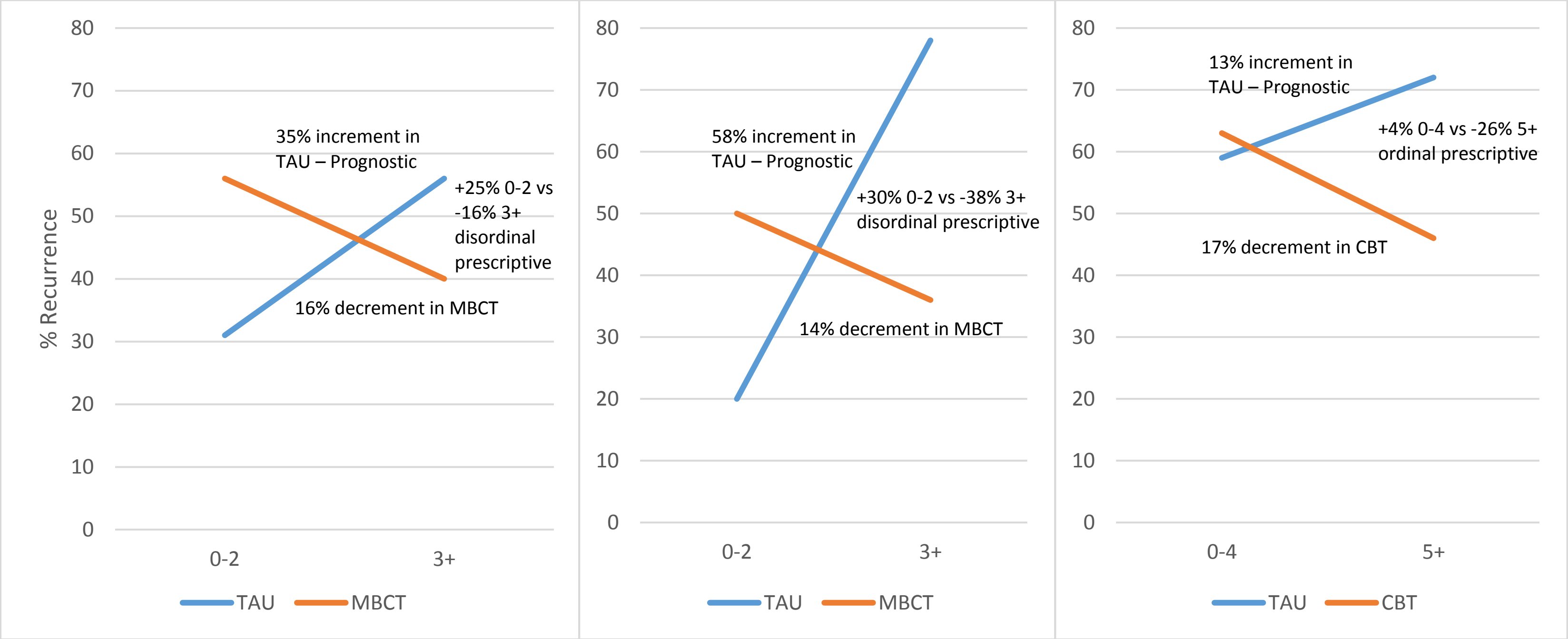

Source: Left panel – Teasdale et al. (2000); Middle Panel – Ma & Teasdale (2004); Right Panel – Bockting et al. (2005).

Supplementary Figure 4. Flow diagram of study selection for Study 2.

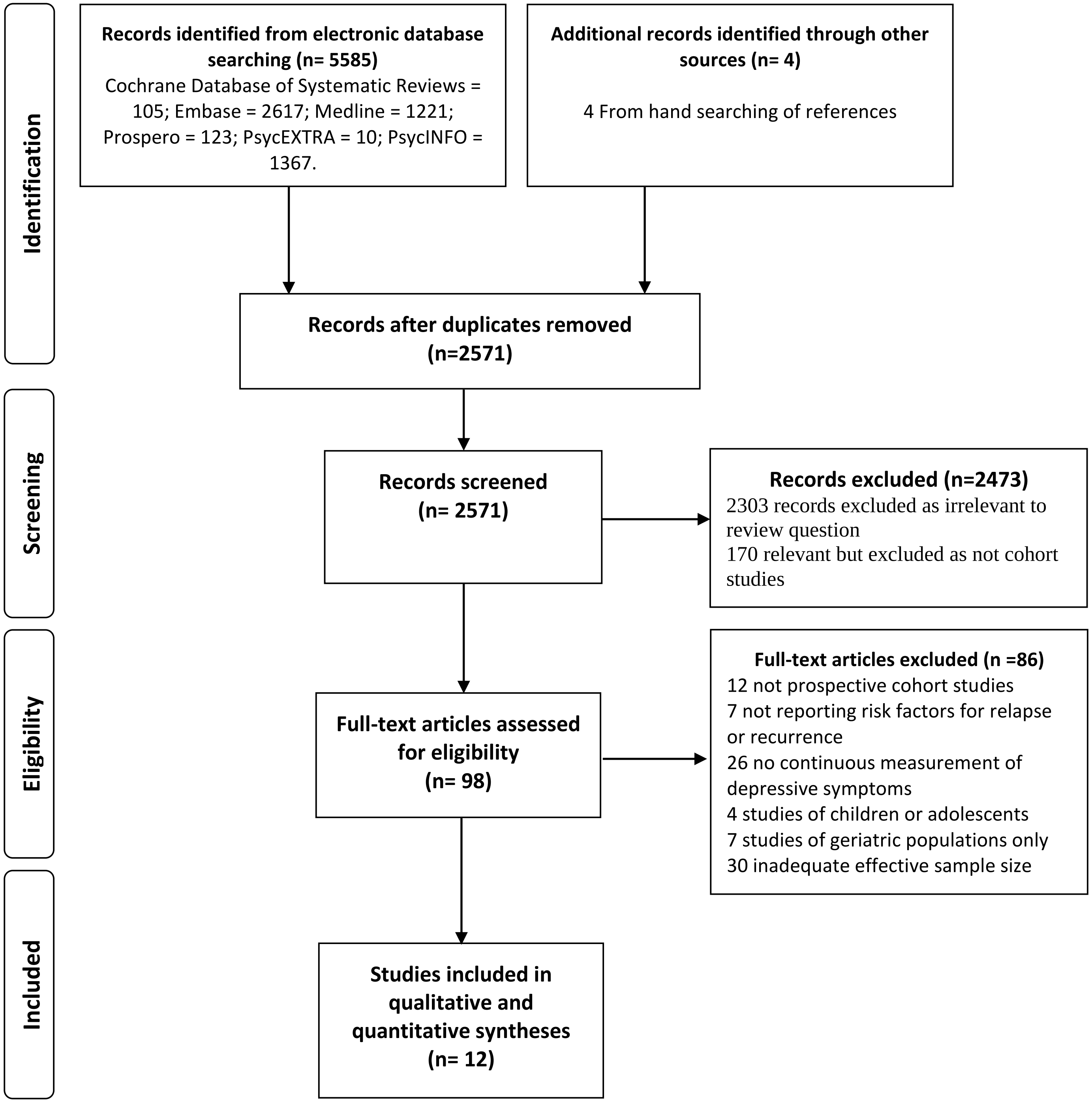
Identification
Records identified from electronic database searching (n= 5585)
Cochrane Database of Systematic Reviews = 105; Embase = 2617; Medline = 1221; Prospero = 123; PsycEXTRA = 10; PsycINFO = 1367.
Additional records identified through other sources (n= 4)
4 From hand searching of references
Records after duplicates removed (n=2571)
Screening
Records screened (n= 2571)
Records excluded (n=2473)
2303 records excluded as irrelevant to review question
170 relevant but excluded as not cohort studies
Eligibility
Full-text articles assessed for eligibility (n= 98)
Full-text articles excluded (n =86)
12 not prospective cohort studies
7 not reporting risk factors for relapse or recurrence
26 no continuous measurement of depressive symptoms
4 studies of children or adolescents
7 studies of geriatric populations only
30 inadequate effective sample size
Included
Studies included in qualitative and quantitative syntheses (n= 12)

Supplementary Table 3. Additional data extracted from included cohort studies.

Reviewed Study and overall Cohort	Setting & Sample	How Depression was diagnosed at baseline	How relapse or recurrence were defined and determined	N at baseline and follow-up	N first episode depression Vs N previous episodes	Other Sample Characteristics	N Relapses and Recurrences
Judd et al., 1998 **CDS**	Secondary care outpatient and inpatient. Ethnically white, English speaking adults seeking treatment at one of 5 centres with RDC confirmed MDE without dysthymia, and who completed follow-up at 10 years post-baseline.	SADS to make RDC diagnosis	LIFE every 6 months for 5 years then yearly; chronological memory prompts were given to get accurate recall of weekly symptom severity; every 5 years also cross check for relapse/recurrence from medical and research records.	237 at 10 year follow-up	59 had ≥4 previous episodes, numbers with 0, 1, 2 and 3 previous episodes were not given.	Demographics: Mean age 39.9(15.5), 62.4% female, 55.3% married/cohabiting, 52.3% College or more education. Clinical: 72.6% Inpatients at intake, mean age of onset 32.1(14.5), weeks duration of index episode 20.7% 0-6months, 20.7% 6months-1year, 28.3% 1-2years, 30.4% 2+ years, mean GAS score 30.11(10.6), mean weekly CAD for ADM 1.69(1.10).	173 all recurrences (to any depressive episode; 85 MDD, 88 first to MinD of which 28 merged with MDD and 27 later had MDD, so 33 had no MDD)
Mueller et al., 1999 **CDS**	As above except without requiring all to have completed 10-year follow-up and including 15-years of follow-up	As above	As above	431, 380 at 15 years	141 vs 99	Of those that had a recurrence: Age 37.7(14.7), 64% female, 32% never married, 59% primary MDD at intake 10% Psychotic subtype, mean HAM-D 20.3(7.1), mean GAS 40.4(11), mean weeks duration of index depressive episode 68(108). Of those without a recurrence: Age 39.6(14.2), 51% female, 19% never married, 59% primary MDD at intake, 7% Psychotic subtype, HAM-D 19.4(7.3), GAS 42.3(10.8), weeks duration of index episode 50(85).	279 all recurrences
Solomon et al., 2000 **CDS**	Similar to above but included only MDD patients who had recovered from index episode over 10 years of follow-up.	As above	As above	318, 263 at 5 years, 208 at 10 years	121 vs 197 (76 with 1 episode, 41 with 2 episodes, 80 with ≥3 episodes)	Demographics: Mean(SD) age 39(15), 59% female, 54% married or cohabiting, SES class 16% III, 35% IV, 17% V. Clinical: Mean GAS 44(12), Mean HDRS 25(7), 74% Inpatients, 38% 0 previous episodes, 24% 1, 13% 2, 25% 3+; 89% probable or definite Endogenous Depression, 8% Psychotic, 59% primary (Current) depression.	202 all recurrences

Hardeveld et al., 2013a<br><br>**NEMESIS**	Community setting. Adults with lifetime MDD in remission for at least 6 months	CIDI Lifetime Version	CIDI with life chart interview at 1 and 3 years	836, 687 at 1 year, 590 at 3 years	323 vs 364	68% female; 41% low education; 74% married/cohabiting; 70% employed; 32% severe index episode; 40% comorbid anxiety disorders; 48% comorbid somatic illness over 12 months; 28% childhood trauma	135, recurrences, did not allow for relapse as inclusion criteria required 6 months or more without MDE pre-baseline
Wang et al., 2014<br><br>**NESARC**	Community, primary care, secondary care and emergency room. Adults with current or lifetime MDE, remitted for at least 2 months pre-baseline, sought or received professional help to improve mood.	AUDADIS-IV lifetime and past-year versions	AUDADIS-IV for diagnoses since baseline.	1518 development sample, 1195 validation sample, 3 year follow up response rate was 86.7%, missing data were imputed by Hot-deck method so all included in analysis		Validation sample only: Demographics: 74.9% female, mean age 45.4(0.41). 48.8% married, 29.5% household income over $60k, 78.41% white, 16.0% hospitalised due to depression, 14.2% went to emergency room	307 (validation sample) 382 (in development sample), all recurrences as inclusion criteria included being in remission for at least two months pre-baseline
Dijkstra-Kersten et al., 2017<br><br>**NESDA**	Community, primary care and secondary care. 628 adults with past depressive or anxiety episodes remission for at least six months	CIDI Lifetime Version at baseline	CIDI with life chart interview at 2 and 4 post-baseline. Recurrence defined as mild symptoms or worse after remission, for at least 1 month, and meeting CIDI criteria for MDD during follow-up	628, 574 at 4 years	115 with 0 previous episodes, 235 with 1, 173 with ≥2	Demographics: 71.1% female, mean age 44.2, education in years 12.6(3.19). Clinical: number of chronic somatic diseased 0(58.9%), 1(28.4%), >=2(12.7%), above cut-off for Multiple physical symptoms 29%, Neuroticism score 20.7(7.51), Mastery score 18.9(3.69), number of previous depressive episodes: 0 22.0%, 1 44.9%, >=2 33.1%, subclinical depressive symptoms score 14.0(8.72), subclinical anxiety symptoms score 7.07(6.38)	Recurrences: 121 between baseline and 2 years, 93 between 2 and 4 years

Gerrits et al., 2014<br><br>**NESDA**	Community, primary and secondary care. 18-65 year olds with lifetime depressive or anxiety disorders remitted for at least 6 months prior to baseline.	CIDI Lifetime Version at baseline	As above	1263, 1122 at 4 years	0 first episodes	Demographics: 68.2% Female, Mean age 43.4(12.8), mean years education 12.5(3.2). Clinical: Recency of latest episode pre-baseline <= 1 year = 14.3% (includes dep and anx), 48.4% history of both dep and anx, mean QIDS 5.4(3.7), mean BAI 7.2(6.4), mean pain locations 1.2(1.9), duration of pain >=90 days 31.6%, 64.2% Chronic Pain Grade 0-1, 21.9% CPG 2, 9% CPG 3.	292 recurrences, did not allow for relapse as inclusion criteria required 6 months or more without MDE pre-baseline
Hardeveld et al., 2015<br><br>**NESDA**	As above	As above	As above	810, 683 at 4 year follow-up	0 Vs 333	71.7% female, mean age 43.7(12.6), 51.5% Childhood Trauma, 35.1% past year life events, 22.5% on ADM	250 recurrences, did not allow for relapse as inclusion criteria required 6 months or more without MDE pre-baseline
Hardeveld et al., 2014<br><br>**NESDA**	As above	As above	As above	770, 702 at 4 years, only 549 with usable saliva samples for cortisol analysis	282 first episodes	Demographics: 71.4% female, mean age 45.0. Clinical: mean previous episodes 2.9, 88.2% remitted >12 months prior to baseline, 41.3% no depressive symptoms in 5 years prior to baseline, mean IDS score 17.2(9.9)	193 recurrences, did not allow for relapse as inclusion criteria required 6 months or more without MDE pre-baseline
Hardeveld et al. 2013b<br><br>**NESDA**	Similar to above but restricted sample to those with MDD diagnosis in 6 months prior to baseline, symptomatic one month prior to baseline, and achieved remission during follow-up. Only included if sought care for mental health in 6 months pre-baseline and had at least two contacts with clinicians regarding mental health in that time	As above	As above but restricted to two years follow-up	706, 566 at two years, 375 after exclusions for not achieving remission and changes in original diagnoses	181 vs 194 with at least 1 previous episode	Demographics: 66.9% female, mean age 40.3(11.8), education mean years 11.4(3.0). Clinical: Mean age of onset 38.1(12.9), family history 86.1%, 51.8% with Recurrent MDD (previous episodes), Severity of last MDE - 30.1% Mild, 31.0% Moderate, 38.9% Severe, 65.9% with comorbid anxiety disorder within 6 months of basline, 31.5% history of alcohol abuse/dependence, mean somatic illnesses 0.65(0.98). Psychological: mean childhood trauma score 1.2(1.2), mean negative life events 0.98(1.16), Neuroticism mean score 30.6(6.5). Treatment: 61.1% ADM, 58.7% Psychological.	119 – all labelled as recurrences but some were relapses – numbers of each could not be confirmed. Median time to recurrence was 5.5 months in the primary care group and 5.0 months in the specialized care group.

Spinhoven et al., 2016<br><br>**NESDA**	Adults aged 18-65, 3 subgroups from NESDA study i) depression or dysthymia over last six months, ii) history of depression or dysthymia in remission for at least six months, iii) healthy controls	As above	As above but included six years of follow-up	2596 (of which 1150 at risk of recurrence), 2256 at six years (977 of those at risk of recurrence)	Unknown	Of group at risk of recurrence (n=1150): 68.7% female, age 43.9(12.7), years education 12.4(3.3), depressive symptom severity score 14.7(9.5), GAD diagnosis 3.9%, Social Anxiety Disorder 11.5%, Panic Disorder 7.7%, Agoraphobia 5.7%, Rumination score 8.4(4.7), worry score 28.5(10.8), Neuroticism score 33.7(7.7).	360 recurrences
Gopinath et al., 2007<br><br>N/A	From RCT of patients with chronic depression in primary care. 18 to 80 year olds prescribed new ADM with a diagnosis of depression or anxiety. All recovered from episode 8 weeks later with history of ≥3 MDEs or dysthymia and one of the following: (a) <4 DSM-IV major depressive symptoms and/or (b) >4 residual depressive symptoms but with a mean SCL-20 score of <1.0.	SCID	SCL-20, current depression and dysthymia modules of SCID and LIFE, all by telephone interview with researchers blind to allocation at 3, 6, 9, and 12 months. "Relapse" was defined as meeting DSM-IV criteria on SCID or the LIFE in the preceding 3 months before each interview.	386, 336 at 12 months	0 first episode, 256 had ≥ 3 previous episodes	Demographics: Mean age 46.0(12.7), 73.7% Female, 87.6% at least 1 year college education, 90.2% White, 78% Employed. Clinical: mean SCL-20 0.84(0.37), mean Chronic Depression Score 1030(1111.9), Mean NEO Neuroticism 3.03(0.73), 75.4% Recurrent Depression (>=3 past episodes), 83.0% MDD in last 2 years, 49.2% Dysthymia, 81.4% given ADM at baseline, 12.6% comorbid panic disorder	120 recurrences, did not allow for relapses as inclusion criteria included being recovered at baseline.

Quality rating of Cohorts reviewed in Study 2:

Although GRACE was designed primarily for use with comparative effectiveness studies, it was deemed the best available tool to give a framework for considering the quality of the cohort studies reviewed. More thorough systems such as STROBE (von Elm et al., 2007) are available but focus more on the reporting of constituent studies than their design and methods per se. The GRACE Checklist includes six items related to data and five related to methods, with the rated study considered sufficient or insufficient on each item. The specific items are: D1) Were treatment or important details of treatment exposure adequately recorded for the study purpose in the data sources? Note: not all details of treatment are required for all research questions; D2) Were the primary outcomes adequately recorded for the study purpose (e.g., available in sufficient detail through data sources)?; D3) Was the primary *clinical* outcome measured objectively rather than left solely to clinical judgment (e.g., opinion about whether the patient's condition has improved)?; D4) Were primary outcomes validated, adjudicated, or otherwise known to be valid in a similar population?; D5) Was the primary outcome measured or identified in an equivalent manner between the treatment/intervention group and the comparison groups?; D6) Were important covariates that may been confounds or moderators available and recorded? Important covariates depend on the treatment and/or outcome of interest (e.g., body mass index should be available and recorded for studies of diabetes; race should be available and recorded for studies of hypertension and glaucoma); M1) Was the study (or analysis) population restricted to new initiators of treatment or those starting a new course of treatment? Efforts to include only new initiators may include restricting the cohort to those who had a washout period (specified period of medication non use) prior to the beginning of study follow-up; M2) If one or more comparison groups were used, were they concurrent comparators? If not, did the authors justify the use of historical comparison groups?; M3) Were important confounding and moderating variables taken into account in the design or analysis? Appropriate methods to take these variables into account may include restriction, stratification, interaction terms, multivariable analysis, propensity score matching, instrumental variables, or other approaches; M4) Is the classification of exposed and unexposed person-time free of "immortal time bias"? (Immortal time in epidemiology refers to a period of cohort follow-up time during which death, or an outcome that determines end of follow-up, cannot occur); and M5) Were any meaningful analyses conducted to test key assumptions on which primary results are based? (E.g., were analyses reported to evaluate the potential for a biased assessment of exposure or outcome, such as analyses where the impact of varying exposure or outcome definitions was tested to examine the impact on results?).

Supplementary Table 4. GRACE Quality Ratings of Reviewed Cohort Studies

Study Citation	D1: Were treatment and/or important details of treatment exposure adequately recorded for the study purpose in the data sources?	D2: Were the primary outcomes adequately recorded for the study purpose (e.g., available in sufficient detail through data sources)?	D3: Was the primary *clinical* outcome measured objectively rather than subject to clinical judgment?	D4: Were primary outcomes validated, adjudicated, or otherwise known to be valid in a similar population?	D5: Was the primary outcome measured or identified in an equivalent manner between the treatment/intervention group and the comparison groups?	D6: Were important covariates that may be known confounders or effect modifiers available and recorded?	M1: Was the study (or analysis) population restricted to new initiators of treatment or those starting a new course of treatment?	M2: If 1 or more comparison groups were used, were they concurrent comparators? If not, did the authors justify the use of historical comparison groups?	M3: Were important confounding and effect modifying variables taken into account in the design and/or analysis?	M4: Is the classification of exposed and unexposed person-time free of "immortal time bias"?	M5: Were any meaningful analyses conducted to test key assumptions on which primary results are based?
Judd et al., 1998	+	+	+	+	+	+	+	+	-	-	-
Mueller et al., 1999	+	+	+	+	+	-	+	+	+	-	-
Solomon et al., 2000	+	+	+	+	+	-	+	+	+	-	-
Hardeveld et al., 2013a	-	+	+	+	+	+	+	+	+	-	-
Wang et al., 2014	-	+	+	+	+	+	+	+	+	+	+
Dijkstra-Kersten et al., 2017	-	+	+	+	+	+	+	+	+	-	-
Gerrits et al., 2014	-	+	+	+	+	-	+	+	-	+	-
Hardeveld et al., 2015	-	+	+	+	+	-	+	+	-	-	-
Hardeveld et al., 2014	-	+	+	+	+	-	+	+	-	-	-
Hardeveld et al. 2013b	+	+	+	+	+	+	+	+	+	-	-
Spinhoven et al., 2016	_	+	+	+	+	+	-	+	+	+	+
Gopinath et al., 2007	+	+	+	+	+	+	+	+	+	+	-

Supplementary Table 5. Data extracted from included cohort studies.

Reviewed Study and overall Cohort	Setting & Sample	How relapse or recurrence were defined and determined	N at baseline and follow-up	N Relapses and Recurrences	Results of relevance to recurrence
Judd et al., 1998<br><br>**CDS**	Secondary care outpatient and inpatient. Ethnically white, seeking treatment, RDC confirmed MDE without dysthymia, completed follow-up at 10 years.	LIFE every 6 months first 5 years then yearly thereafter; chronological memory prompts were given to get accurate recall of weekly symptom severity; every 5 years also cross check for relapse/recurrence from medical and research records.	237 at 10 year follow-up	173 all recurrences (to any depressive episode; 85 MDD, 88 first to MinD of which 28 merged with MDD and 27 later had MDD, so 33 had no MDD)	Early relapse was associated with sub-threshold depressive symptom (SSD) group and having a **history of >4 prior depressive episodes** including the intake episode. Previous episodes **only predicted subsequent risk in the asymptomatic recovery patients and had little effect on the weeks to relapse in the SSD group**. After controlling for recurrent MDEs, patients with residual symptoms were 368% more likely to relapse than the asymptomatic group OR(95%CI)=3.68(2.64-5.12). In contrast **the >4 episodes factor after controlling for residual symptoms increase the risk by only 64%** OR(95%CI)=1.64(1.17-2.29).
Mueller et al., 1999<br><br>**CDS**	As above except without requiring all to have completed 10-year follow-up and including 15-years of follow-up	As above	431, 380 at 15 years	279 all recurrences	**Women** (OR(95%CI)=1.43(1.10-1.86), p=.007), **never married** (OR(95%CI)= 1.55(1.14-2.10), p=.005), **duration of index episode greater than 1 year** (per year OR(95%CI) = 1.11(1.05-1.18), p=.0004), all had **greater likelihood of recurrence**. **Each additional episode of major depression before intake was associated with an 18% increase in the risk of recurrence** OR(95%CI) = 1.18(1.06-1.31), p=.002. When consider any vs no previous episodes OR(95%CI)= 1.81(1.14-2.88), comparing 3+ vs <3 prior episodes OR(95%CI)= 2.06(1.15-3.68). When calculating **effect of previous episodes in subsample that remained well/did not have a recurrence for at least 5 years OR(95%CI)=1.43(0.56-3.64)**. **Baseline severity was not related to recurrence** (OR(95%CI)=1.00(0.95-1.05)). **Age and primary versus secondary depressive episode did not distinguish the two groups** in this long-term study, a finding contrary to the findings of reports from the collaborative depression study based on briefer follow-up periods. Subsyndromal symptoms predict a 3-fold shorter time to recurrence. Measured prospectively **subsyndromal symptoms appear to be a stronger predictor than all baseline measures (including number of previous episodes).**
Solomon et al., 2000<br><br>**CDS**	Similar to above but included only MDD patients who had recovered from index episode over 10 years of follow-up.	As above	318, 263 at 5 years, 208 at 10 years	202 all recurrences	The **number of lifetime episodes** of major depressive disorder was significantly **associated with recurrence** during the 10-year follow-up period (OR(95%CI) =1.16 (1.03–1.31) p=.02). Thus, for each successive episode of major depression, the risk of recurrence increased by 16%. **As duration of recovery increases risk of recurrence decays.**
Gopinath et al., 2007<br><br>**GHC**	From RCT of patients with chronic depression in primary care. 18 to 80 year olds prescribed new ADM with a diagnosis of depression or anxiety. All recovered from episode 8 weeks later with **history of ≥3 MDEs (n=291) or dysthymia (n=95)** and one of the following: (a) <4 DSM-IV major depressive symptoms and/or (b) >4 residual	SCL-20, current depression and dysthymia modules of SCID and LIFE, all by telephone interview with researchers blind to allocation at 3, 6, 9, and 12 months. "Relapse" was defined as meeting DSM-IV criteria on SCID or the LIFE in the preceding 3 months before each interview.	386, 336 at 12 months	120 recurrences, did not allow for relapses as inclusion criteria included being recovered at baseline.	Univariate associations with recurrence: **residual depressive symptoms, higher neuroticism, poorer self-efficacy, poor psychosocial functioning**, **3+ prior depressive episodes** (OR(95%CI) = 1.93(1.11-3.34), **poor medication adherence**, **higher scores on the CTQ abuse scale**, and **comorbid fear or panic symptoms**. **Family history of depression was not related to recurrence** (OR(95%CI)=1.36(0.68-2.73)). Multivariate: only three significant variables: self-efficacy (OR(95%CI)=0.82(0.72-0.93), medication adherence (OR(95%CI)=0.54(0.32-0.90), and the CTQ abuse scales score (OR(95%CI)=1.40(1.04-1.89). No other demographic or clinical variables were found to be significant predictors of relapse in the multivariable model.

	depressive symptoms but with a mean SCL-20 score of <1.0.				
Wang et al., 2014<br><br>**NESARC**	Community, primary care, secondary care and emergency room. Adults with current or lifetime MDE, remitted for at least 2 months pre-baseline, sought or received professional help to improve mood.	AUDADIS-IV for diagnoses since baseline.	1518 development sample, 1195 validation sample, 3 year follow up response rate was 86.7%, missing data were imputed by Hot-deck method so all included in analysis	307 (validation sample) 382 (in development sample), all recurrences as inclusion criteria included being in remission for at least two months pre-baseline	**Family history, duration of longest previous episode of depression, suicidal behaviour, and ongoing life events were associated with recurrence of major depression but did not add to the prediction of recurrence**. The final model contained 19 unique predictors and 4 interaction terms. The C statistics of this model was 0.7504. The model had excellent calibration in the development data (H-L χ2(8) = 10.48, p = .23). **Residual symptoms, physical functioning, childhood trauma or neglect, lifetime history of GAD or specific phobia, diagnosis of avoidant personality disorder, marital status, race, female sex, younger age, suffering racial abuse, physical abuse, and sexual abuse were all associated with recurrence. Number of previous episodes (2+ vs 1 OR(95%CI)=1.25(0.87-1.81); 3+ vs 1 OR(95%CI)=1.31(0.73-1.76)), residual symptoms (most severe OR(95%CI)=2.57(1.69-3.91)), being left alone often before 10 years old OR(95%CI)=1.64(1.03-2.62), being emotionally or physically abused before 18 years old OR(95%CI)=1.88(1.29-2.78)**, accurate prediction of recurrence can only be made when these factors are considered simultaneously. **When only number of previous episodes was used to predict recurrence, the model had a C statistic of 0.5897, similar to a model that included only gender and age (C = 0.5794). Therefore the number of previous depressive episodes alone cannot be used to make accurate predictions of recurrence risk**.
Hardeveld et al., 2013a<br><br>**NEMESIS**	Community setting. Adults with lifetime MDD in remission for at least 6 months	CIDI with life chart interview at 1 and 3 years	836, 687 at 1 year, 590 at 3 years	135, recurrences, did not allow for relapse as inclusion criteria required 6 months or more without MDE pre-baseline	Univariate associations with shorter time to recurrence (presented as HR(95%CI)) with a **history of previous depressive episodes** =1.79(1.26-2.54), **negative youth experiences** =1.82(1.29-2.56), **neuroticism** =1.09(1.05-1.12), **age of onset** =0.98(0.96-0.99), **age at baseline** =0.96(0.94-0.98), **and baseline symptom severity** =2.04(1.30-3.20). Multivariate analyses: younger age =0.96(0.94-0.98), a higher number of previous episodes =1.68(1.15-2.46)), a severe last depressive episode =1.91(1.22-3.00), negative youth experiences =1.59(1.10-2.29), and the presence of ongoing difficulties before recurrence remained significant predictors of time to recurrence. In data-driven post-hoc analysis these had a cumulative effect on risk of recurrence such that respondents with no predictors had a risk of just 3.4%, 1 predictor = 19%, 2 = 26.6%, 3=56.5% and 4+ = 65%
Dijkstra-Kersten et al., 2017<br><br>**NESDA**	Community, primary care and secondary care. 628 adults with past depressive or anxiety episodes remission for at least six months	CIDI with life chart interview at 2 and 4 post-baseline. Recurrence defined as mild symptoms or worse after remission, for at least 1 month, and meeting CIDI criteria for MDD during follow-up	628, 574 at 4 years	Recurrences: 121 between baseline and 2 years, 93 between 2 and 4 years	**Multiple physical symptoms were associated with recurrence** OR(95%CI) = 1.09(1.06-1.12), p<.0001**.** However, those above the binary cut-off for multiple physical symptoms were not at greater odds of recurrence after adjusting for demographics, number of somatic disorders, recency of last depressive episode, number of previous episodes, residual symptoms, neuroticism, mastery and childhood abuse OR(95%CI)=1.36(0.88-2.10), p=.17.

Gerrits et al., 2014<br><br>**NESDA**	Community, primary and secondary care. 18-65 year olds with lifetime depressive or anxiety disorders remitted for at least 6 months prior to baseline.	As above	1263, 1122 at 4 years	292 recurrences, did not allow for relapse as inclusion criteria required 6 months or more without MDE pre-baseline	No association between specific chronic diseases or the number of chronic diseases and recurrence (HR(95%CI) = 1.11(0.96-1.27), p=.16). **Chronic pain grade** (HR(95%CI) = 1.18(1.04-1.35), p=.01), and severe or disabling **neck** (HR(95%CI)=1.45(1.12-1.89),p=.005), **chest** (HR(95%CI) = 1.65(1.14-2.39), p=.008), and **abdominal pain** (HR(95%CI) = 1.52(1.16-2.02), p=.003), **and a higher number of pain locations** (HR(95%CI) = 1.10(1.04-1.16), p=.002) **were all associated with a shorter time to recurrence**, after adjusting for age, gender, years of education and recency of last depressive episode. **Residual depressive symptoms mediated the associations between pain and recurrence** (total effect of mediation between .18 and .77, ps between .09 and .001).
Hardeveld et al., 2015<br><br>**NESDA**	As above	As above	810, 683 at 4 year follow-up	250 recurrences, did not allow for relapse as inclusion criteria required 6 months or more without MDE pre-baseline	**Recurrence of MDD was related to childhood trauma** (p=.003) univariate OR(95%CI)= 1.40(1.02-1.91), **and use of ADM at baseline** (p<.001) (OR(95%CI)=2.21(1.53-3.18)). **No associations were found between the GR and MR polymorphisms and recurrence and there were no significant interactions** found between stress related factors and the GR or MR haplotypes.
Hardeveld et al., 2014<br><br>**NESDA**	As above	As above	770, 702 at 4 years, only 549 with usable saliva samples for cortisol analysis	193 recurrences, did not allow for relapse as inclusion criteria required 6 months or more without MDE pre-baseline	Univariate analyses OR(95%CI)s calculated from data presented in the article: **recurrence was associated with younger age** (p=.03), **>1 past episode of depression** (p<.01) (OR(95%CI)=1.63(1.14-2.31)), **more severe depressive symptoms at baseline** (p<.001), **a 6-month history of anxiety disorders prior to baseline** (p<.001), **a higher number of traumatic youth experiences** (p=.02) OR(95%CI)=1.50(1.05-2.13), **more frequent use of ADM** (p=.001) (OR(95%CI)=2.05(1.36-3.08)), **and had a higher AUCi in CAR** (p=.02). There were no differences between recurrent and non-recurrent subjects in sex, number of negative life events in the past year, family history of depression (OR(95%CI)= 1.36(0.85-2.18)), and covariates related to cortisol levels. Multivariate analyses HR(95%CI)s reported in the article: higher AUCi is associated with time to recurrence of MDD (HR(95%CI) = 1.03 (1.003—1.060), p = 0.03), adjusted for gender, age, smoking time of awakening, CVD, working day and use of ADM. Evening cortisol levels and cortisol suppression after dexamethasone ingestion were not related to time to recurrence. No interactions were found between HPA axis parameters and stress related factors so increased **risk of recurrence with high CAR is not dependent on life events or childhood trauma**

Hardeveld et al. 2013b<br><br>**NESDA**	Restricted sample to those with MDD diagnosis in 6 months prior to baseline, symptomatic one month prior to baseline, and achieved remission during follow-up. Only included if sought care for mental health in 6 months pre-baseline and had at least two contacts with clinicians regarding mental health in that time	As above but restricted to two years follow-up	706, 566 at two years, 375 after exclusions for not achieving remission and changes in original diagnoses	119 – all labelled as recurrences but some were relapses – numbers of each could not be confirmed. Median time to recurrence was 5.5 months in the primary care group and 5.0 months in the specialized care group.	No difference was found in time to recurrence for those treated in primary compared to specialized care centres or for neuroticism scores (HR(95%CI)=1.00(0.97-1.03)). A **family history of depression** (univariate = 2.16(1.09-4.26), multivariable =2.12(1.07–4.22)) **and having a previous MDE** (univariate: 1.61(1.10-2.35), multivariate: =1.59(1.08–2.35)) **were significant predictors of time to recurrence** after adjusting for gender, age, age of onset, and pharmacological treatment (as yes or no). **Age of onset was marginally related to time to recurrence** in univariate analysis HR(95%CI)=0;99(0.98-1.00) but not in multivariable models (1.00(0.98-1.02)), likewise age at baseline – univariate =0.99(0.97-1.00), multivariable = 0.99(0.97-1.01); duration of depression measured as % of months with depression in past year was not associated with recurrence HR(95%CI)=0.87(0.53-1.50).
Spinhoven et al., 2016<br><br>**NESDA**	Adults aged 18-65, 3 subgroups from NESDA study i) depression or dysthymia over last six months, ii) history of depression or dysthymia in remission for at least six months, iii) healthy controls	As above but included six years of follow-up	2596 (of which 1150 at risk of recurrence), 2256 at six years (977 of those at risk of recurrence)	360 recurrences	Experiential avoidance was associated with rumination, worry and neuroticism, each with moderate correlations. It therefore can't be considered an independent risk factor for recurrence, and after controlling for the other psychological factors it was not predictive of recurrence (OR(95%CI)=1.10-0.90-1.36)). **Recurrence was predicted by residual symptoms** at T2 (OR(95%CI) = 2.36(1.96-2.84)), **rumination** (OR(95%CI)=1.83(1.56-2.15)), **worry** (OR(95%CI)=2.04(1.74-2.39)), , **experiential avoidance** (OR(95%CI)= 1.72(1.47-2.03)), **neuroticism** (OR(95%CI)=1.98(1.66-2.35)), **education** (OR(95%CI)=0.94(0.90-0.98)),**comorbid GAD** (OR(95%CI)=2.16(1.14-4.08)), **Social Anxiety** (OR(95%CI)=2.32(1.54-3.49)), **Agoraphobia** (OR(95%CI)=2.16(1.23-3.76)). **but not by: 2 vs 1 previous episodes** OR(95%CI)= 0.89(0.68-1.16); **gender** (OR(95%CI)=1.26(0.95-1.68)); **age** (OR(95%CI)=0.99(0.98-1.00)); or **Panic Disorder** (OR(95%CI)=1.46(0.91-2.34)). In multivariate models adjusted for clinical and demographics variables only residual symptoms, worry and rumination remained as significant predictors of recurrence.

Abbreviations: ADM – Antidepressant Medication; AUCi – Area Under the Curve Increase; CAR – Cortisol Awakening Response; GAD – Generalised Anxiety Disorder; GR – Glucocorticoid Receptor; MR – Mineralcorticoid Receptor; PSWQ – Penn State Worry Questionnaire

Supplementary Table 6. Matrix of factors associated/not associated with recurrence of depression in adults from reviewed cohort studies.

**Study citation and quality rating**	**Factors investigated for their association with relapse or recurrence**									
	Residual Symptoms	Previous Episodes	Childhood maltreatment	Severity of last episode	Duration of last episode	Family History of Depression	Age of onset	Neuroticism	Demographics – Age, Sex, Marital Status, Socio-economic status, Educational History	Other
Judd et al., 1998<br>**CDS**	+++	++								
Mueller et al., 1999<br>**CDS**		++		=	+				Female sex +, never married +, Age =	Primary vs secondary depression =
Solomon et al., 2000<br>**CDS**		++								
Hardeveld et al., 2013a<br>**NEMESIS**		++ (any vs zero)	++	+	+	+	+	+	Younger age +	Life events +
Wang et al., 2014<br>**NESARC**	+++	== (2 vs 1 or 3+ vs 1)	++		+	=			Gender, age, marital status and race all as part of full model +	Concurrent physical health problems +, psychosocial difficulties; comorbid psychiatric disorders ++; Physical, racial or sexual abuse in adulthood +
Dijkstra-Kersten et al., 2017<br>**NESDA**										Multiple physical symptoms + (but when using a binary cut-off of >=11 symptoms =).
Gerrits et al., 2014<br>**NESDA**	++							+		Specific chronic diseases or number of chronic diseases ==, chronic pain grade +, Severe or disabling i) neck pain +, ii) chest pain +, iii) abdominal pain +, higher number of pain locations +
Hardeveld et al., 2015<br>**NESDA**			++						Age +	Use of ADM at baseline +, GR or MR Polymorphisms =, GR or MR gene-stress interactions =
Hardeveld et al., 2014<br>**NESDA**	++	++ (any vs zero)	++	+		=			=	History of Anxiety Disorders in previous 6 months ++, more frequent use of ADM +, higher AUCi in CAR +, negative life events =, cortisol levels =
Hardeveld et al. 2013b<br>**NESDA**		++ (any vs zero)				+	~	=	Age =	Primary vs Secondary Care Setting =

Spinhoven et al., 2016<br>**NESDA**	++	== (2 vs 1)<br>++ (any vs zero)			++	Age =, Gender =, Education =	Experiential avoidance +, rumination +, worry (PSWQ) +. Comorbid GAD +, Social Anxiety +, Panic Disorder =, Agoraphobia +
Gopinath et al., 2007<br>**GHC**			+	==	++		Lower self-efficacy +, lower medication adherence ++; Use of ADM at baseline =

Supplementary Figure 5. Forest plots from sensitivity analyses for meta-analyses of individual prognostic risk factors for recurrence of depression.

1. Two compared to one previous episode removing Wang et al., (2104).

Study or Subgroup	log[Odds Ratio]	SE	Weight	Odds Ratio IV, Random, 95% CI
Mueller 1999	0.4855	0.4232	27.8%	1.62 [0.71, 3.72]
Spinhoven 2016	-0.11653	0.136	72.2%	0.89 [0.68, 1.16]
Wang 2014	0.2231	0.1849	0.0%	1.25 [0.87, 1.80]
**Total (95% CI)**			**100.0%**	**1.05 [0.62, 1.79]**

Heterogeneity: Tau² = 0.08; Chi² = 1.83, df = 1 (P = 0.18); I² = 45%
Test for overall effect: Z = 0.19 (P = 0.85)

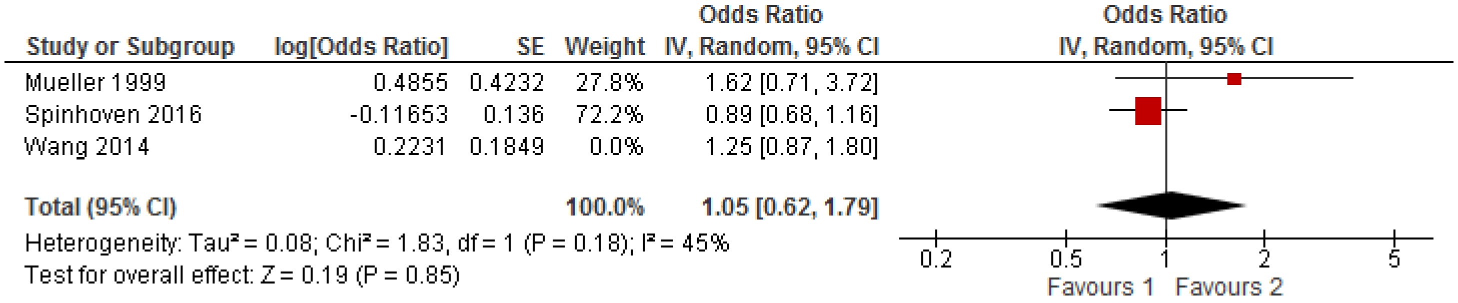

2. Residual symptoms removing data from Wang et al., (2014).

Study or Subgroup	log[Odds Ratio]	SE	Weight	Odds Ratio IV, Random, 95% CI
Judd 1998	1.3029	0.16897	45.1%	3.68 [2.64, 5.12]
Spinhoven 2016	0.8566	0.0946	54.9%	2.36 [1.96, 2.83]
Wang 2014	0	0		Not estimable
**Total (95% CI)**			**100.0%**	**2.88 [1.86, 4.45]**

Heterogeneity: Tau² = 0.08; Chi² = 5.31, df = 1 (P = 0.02); I² = 81%
Test for overall effect: Z = 4.76 (P < 0.00001)

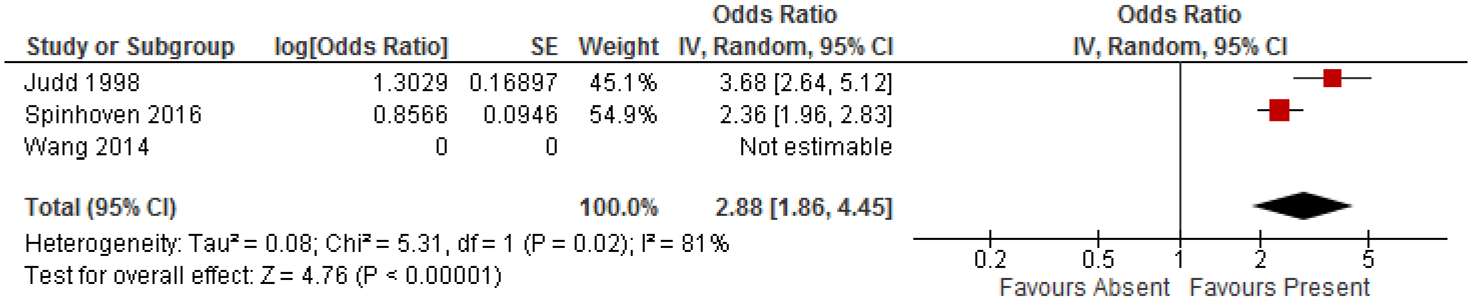

3. Childhood maltreatment removing data from Wang et al., (2014).

Study or Subgroup	log[Odds Ratio]	SE	Weight	Odds Ratio IV, Random, 95% CI
Gopinath 2007	0.3365	0.1524	38.2%	1.40 [1.04, 1.89]
Hardeveld 2014	0.4055	0.18044	27.2%	1.50 [1.05, 2.14]
Hardeveld 2015	0.3336	0.16002	34.6%	1.40 [1.02, 1.91]
Wang 2014	0	0		Not estimable
**Total (95% CI)**			**100.0%**	**1.43 [1.19, 1.71]**

Heterogeneity: Tau² = 0.00; Chi² = 0.11, df = 2 (P = 0.95); I² = 0%
Test for overall effect: Z = 3.76 (P = 0.0002)

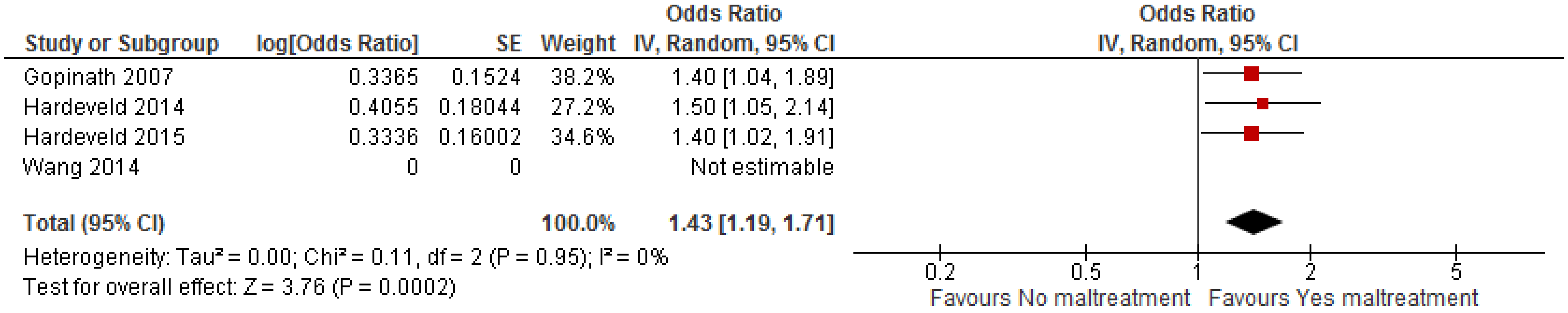

4. Childhood Maltreatment using often left alone before 10 years old from Wang study.

Study or Subgroup	log[Odds Ratio]	SE	Weight	Odds Ratio IV, Random, 95% CI
Gopinath 2007	0.3365	0.1524	33.0%	1.40 [1.04, 1.89]
Hardeveld 2014	0.4055	0.18044	23.5%	1.50 [1.05, 2.14]
Hardeveld 2015	0.3336	0.16002	29.9%	1.40 [1.02, 1.91]
Wang 2014	0.4947	0.2373	13.6%	1.64 [1.03, 2.61]
**Total (95% CI)**			**100.0%**	**1.45 [1.22, 1.72]**

Heterogeneity: Tau² = 0.00; Chi² = 0.41, df = 3 (P = 0.94); I² = 0%
Test for overall effect: Z = 4.27 (P < 0.0001)

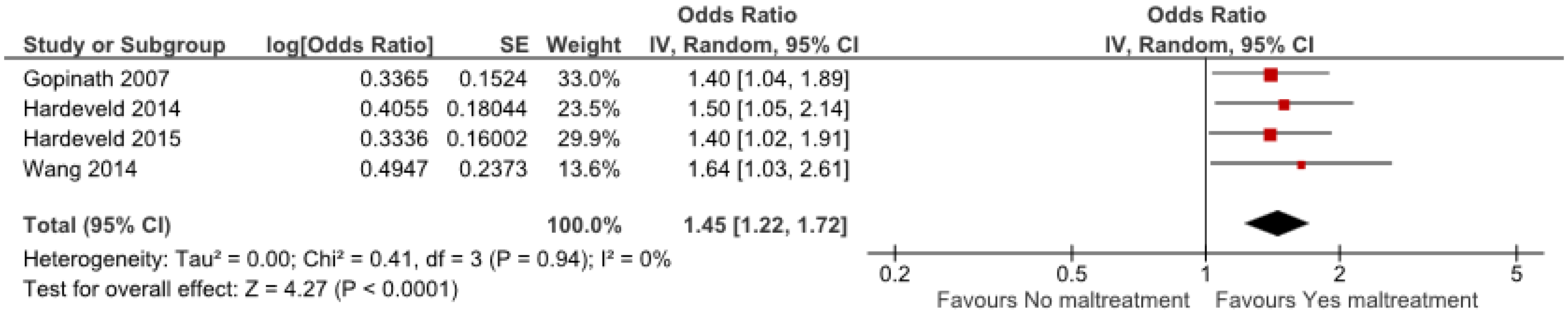

5. Hazard of recurrence for any compared to no previous episodes.

Study or Subgroup	log[Hazard Ratio]	SE	Weight	Hazard Ratio IV, Random, 95% CI
Hardeveld 2013a	0.5188	0.1934	51.0%	1.68 [1.15, 2.45]
Hardeveld 2013b	0.4637	0.1973	49.0%	1.59 [1.08, 2.34]
**Total (95% CI)**			**100.0%**	**1.64 [1.25, 2.14]**

Heterogeneity: Tau² = 0.00; Chi² = 0.04, df = 1 (P = 0.84); I² = 0%
Test for overall effect: Z = 3.56 (P = 0.0004)

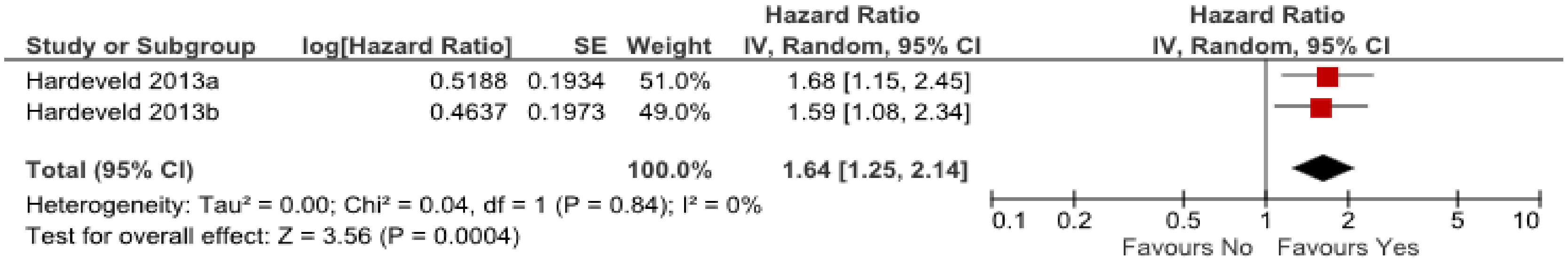

6. Odds of recurrence recent or past comorbid anxiety disorders using Social Anxiety Disorder (Sphinhoven)

Study or Subgroup	log[Odds Ratio]	SE	Weight	Odds Ratio IV, Random, 95% CI
Spinhoven 2016	0.8416	0.2091	37.7%	2.32 [1.54, 3.50]
Wang 2014	0.5188	0.1348	62.3%	1.68 [1.29, 2.19]
**Total (95% CI)**			**100.0%**	**1.90 [1.40, 2.58]**

Heterogeneity: Tau² = 0.02; Chi² = 1.68, df = 1 (P = 0.19); I² = 41%
Test for overall effect: Z = 4.09 (P < 0.0001)

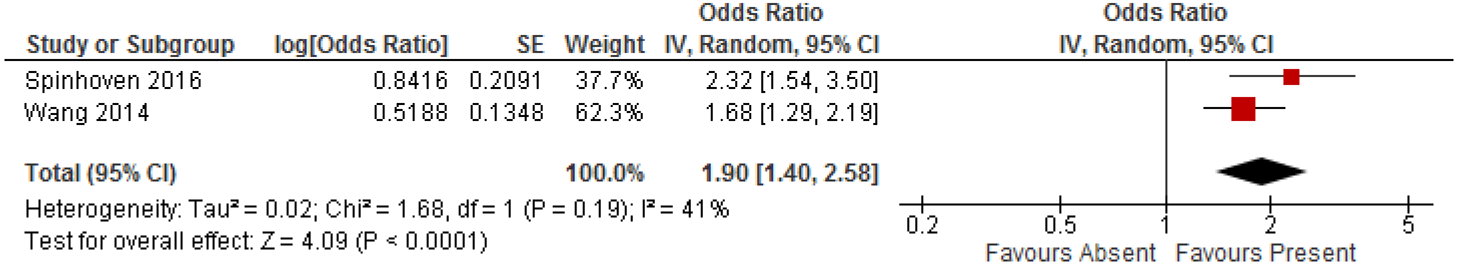

7. Odds of recurrence recent or past comorbid anxiety disorders using Agoraphobia (Sphinhoven)

Study or Subgroup	log[Odds Ratio]	SE	Weight	Odds Ratio IV, Random, 95% CI
Spinhoven 2016	0.7701	0.2873	18.0%	2.16 [1.23, 3.79]
Wang 2014	0.5188	0.1348	82.0%	1.68 [1.29, 2.19]
**Total (95% CI)**			**100.0%**	**1.76 [1.38, 2.23]**

Heterogeneity: Tau² = 0.00; Chi² = 0.63, df = 1 (P = 0.43); I² = 0%
Test for overall effect: Z = 4.62 (P < 0.00001)

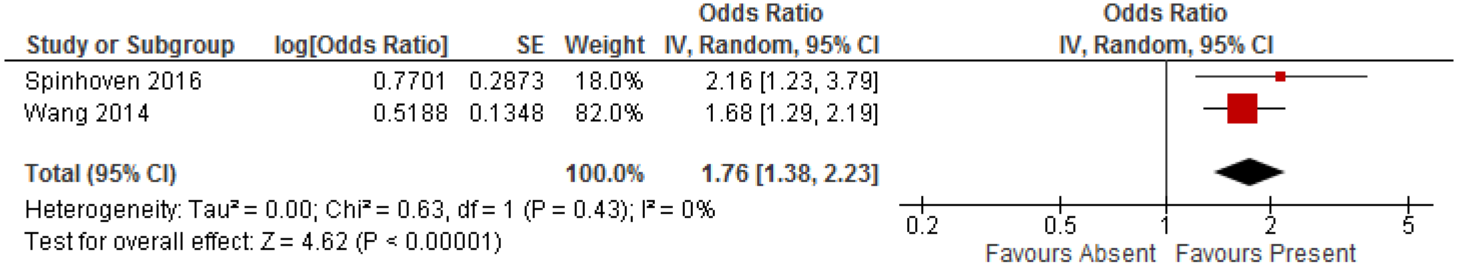

8. Odds of recurrence recent or past comorbid anxiety disorders using Panic Disorder (Sphinhoven)

Study or Subgroup	log[Odds Ratio]	SE	Weight	Odds Ratio IV, Random, 95% CI
Spinhoven 2016	0.3784	0.2412	23.8%	1.46 [0.91, 2.34]
Wang 2014	0.5188	0.1348	76.2%	1.68 [1.29, 2.19]
**Total (95% CI)**			**100.0%**	**1.62 [1.29, 2.05]**

Heterogeneity: Tau² = 0.00; Chi² = 0.26, df = 1 (P = 0.61); I² = 0%
Test for overall effect: Z = 4.12 (P < 0.0001)

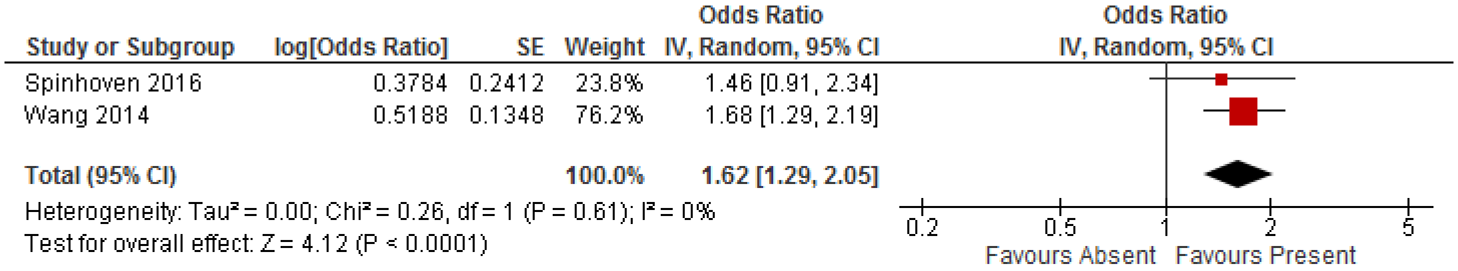

Supplementary Figures 6a-6e. Demonstration of probability of recurrence with each increasing episode in the CDS study.

Supplementary Figure 6a. Cumulative percentage relapsing by the number of prospectively measured episodes (source: Solomon et al., 2000).

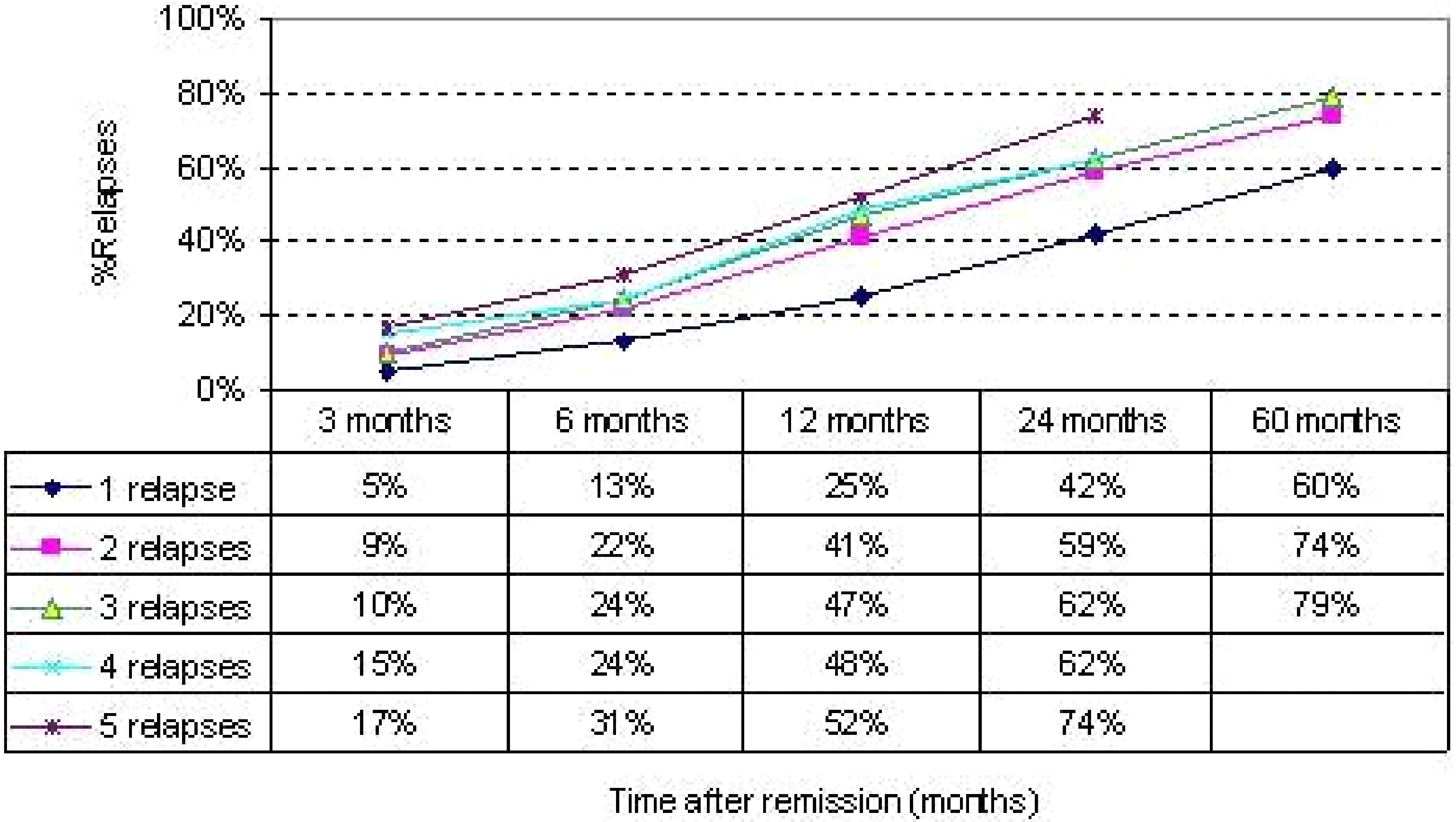

	3 months	6 months	12 months	24 months	60 months
1 relapse	5%	13%	25%	42%	60%
2 relapses	9%	22%	41%	59%	74%
3 relapses	10%	24%	47%	62%	79%
4 relapses	15%	24%	48%	62%	
5 relapses	17%	31%	52%	74%	

Time after remission (months)

Note: Recur 1 depicts the survival curve for the first recurrence any patient experience whereas Recur 2 depicts the survival curve for the subset of patients who had a second recurrence and so forth. In essence, fewer and fewer patients have more and more recurrences but when they do they tend to happen faster than the earlier recurrences did. Fifth episodes happen faster to fewer people than 4th episodes whereas 4th episodes happen faster than 3rd episodes and so forth again. As in Figure 3a (which is just the inverse of this way of representing this data) the biggest visible difference comes between the 1st and 2nd recurrences with little visible difference among the rest.

Supplementary Figure 6b. Time to Recurrence as a Function of Successive Recurrences (Source: Solomon et al., 2000)

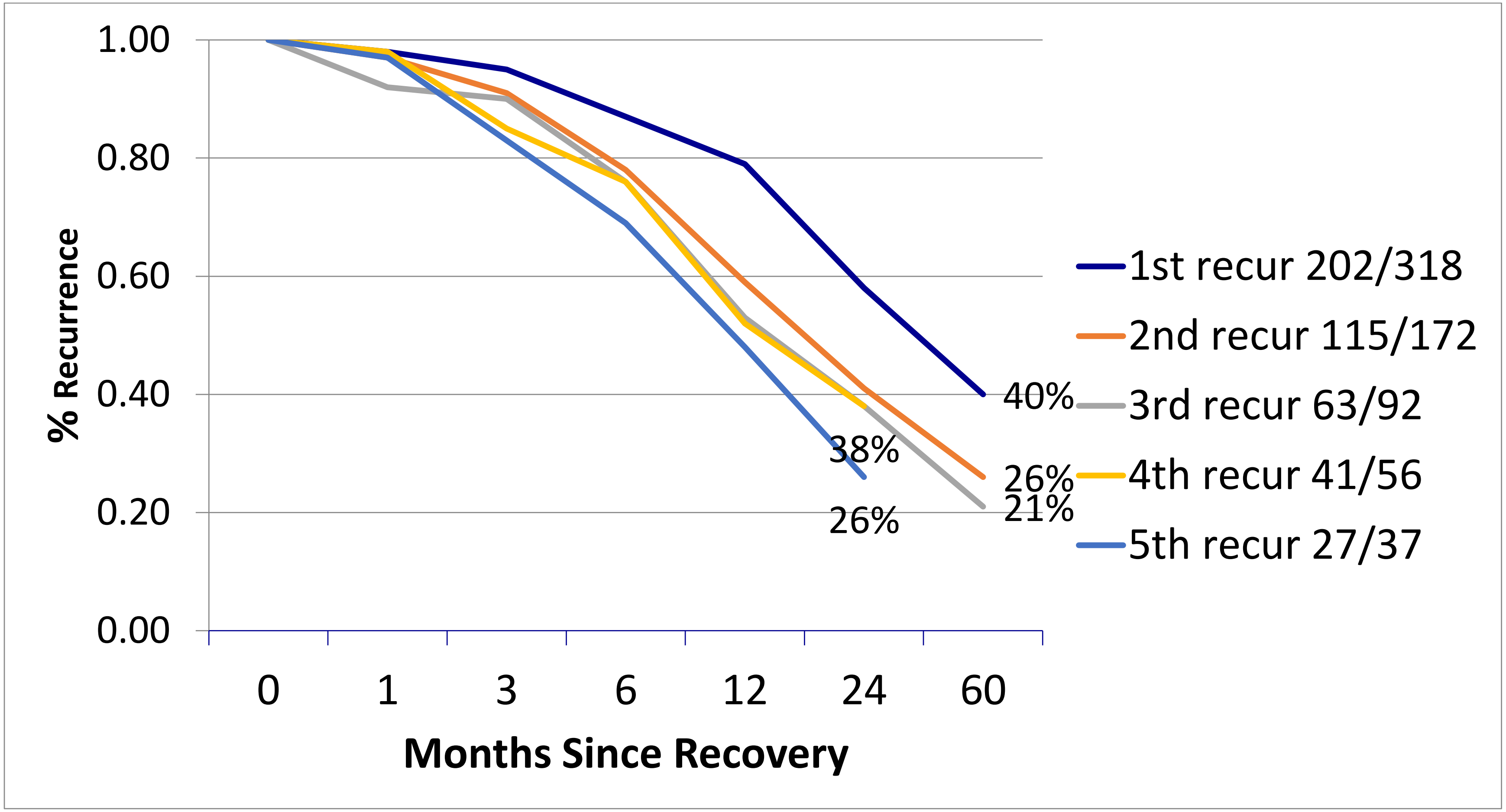

Supplementary Figure 6c. Odds ratio and 95% confidence intervals for subsequent recurrence by each prospectively observed episode (Source: Solomon et al., 2000)

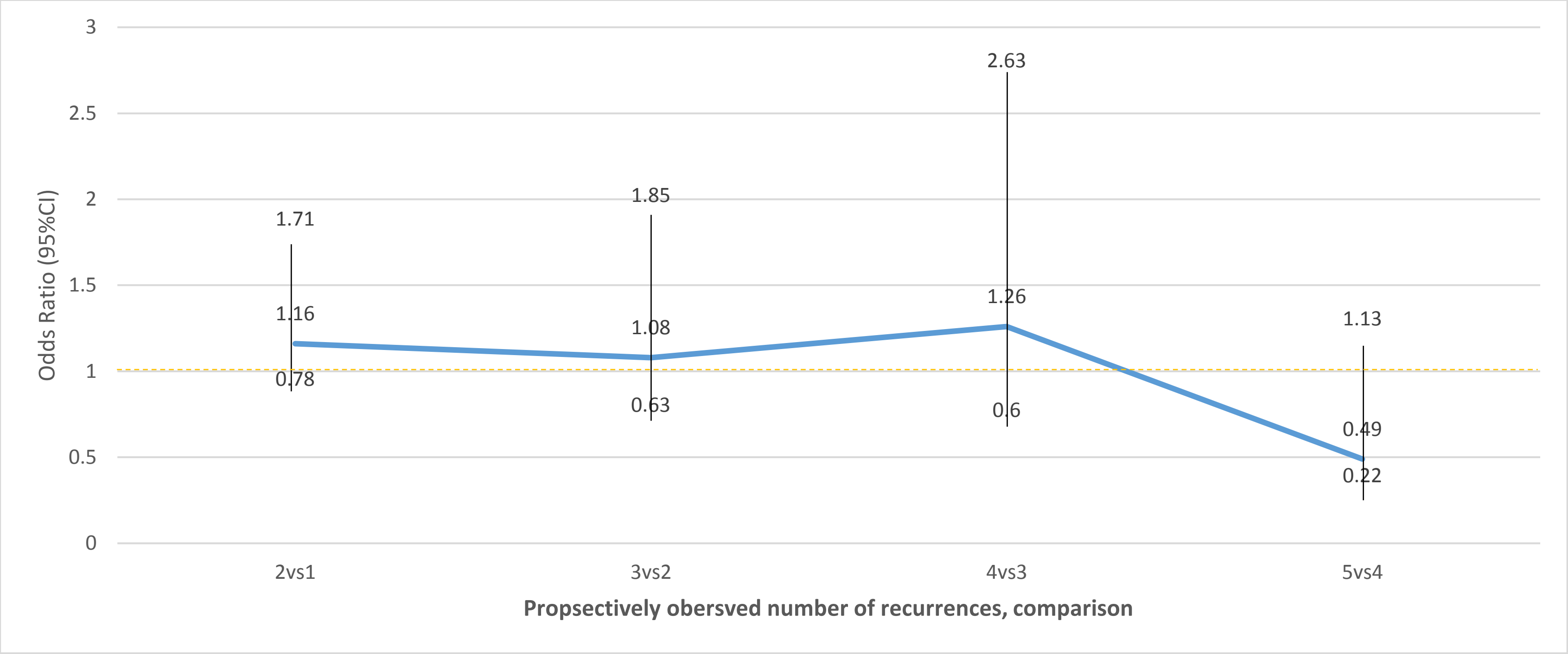

Supplementary Figure 6d. Percentage of recurrences by number of pre-baseline prior episodes in CDS (Source: Mueller et al., 1999).

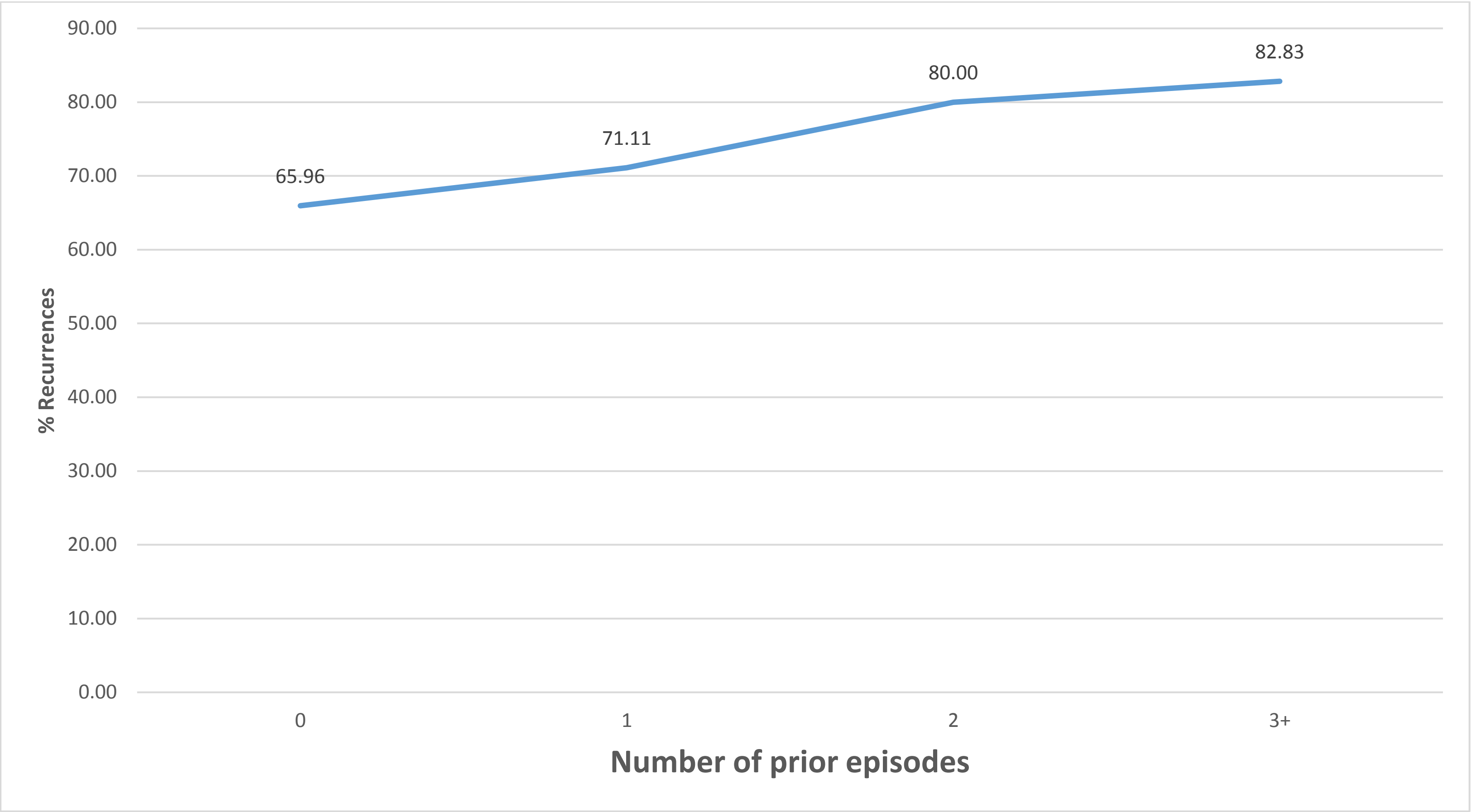

Note: Supplementary Figure 6d shows that higher proportions of participants suffered recurrences over 10 years of follow-up in the CDS with each prior episode.

Supplementary Figure 6e. Odds Ratios and (95% Confidence Intervals) of Recurrence by Episode in CDS (Source: Mueller et al., 1999).

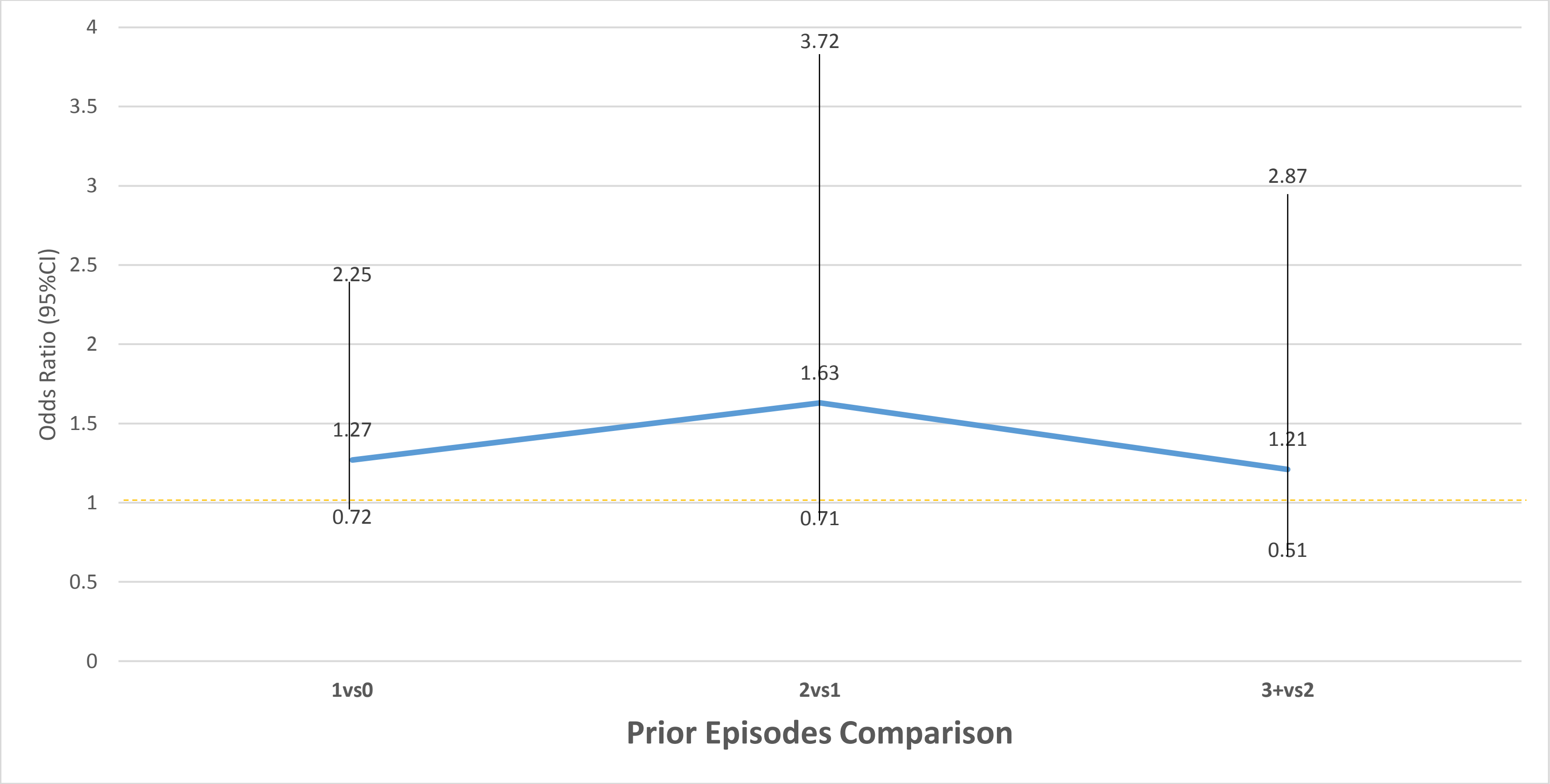

Note: Figure 3e shows that although more participants with more prior episodes have recurrences compared to those with fewer episodes, the odds of having a recurrence do not continue to increase by much, or incrementally compared to one less episode.

Supplementary Discussion of Prognostic Effect of Prior Episodes.

Three or more compared to less than three previous episodes was found to be a risk factor in GHC supporting findings from the CDS cohort, in GHC nearly all cohort members had minimal residual symptoms, and controlling for residual symptoms in a multivariate model removed the effect of previous episodes, which appears to fit with the finding of Judd et al. (1998) in CDS participants that suggested multiple previous episodes is a particularly strong risk factor among asymptomatic patients but not so strong among those with residual symptoms. There were a number of factors that may have effected these comparisons including the lack of consistency in how previous episodes were measured and reported, the heterogeneous samples being compared across the cohorts, the lack of measurement or control of treatment in the studies reviewed herein, and the bias introduced by drop-out during follow-up in the cohorts. However, adding weight to a non-monotonic relationship between increasing numbers of prior episodes and recurrence the odds ratio for two or more compared to less than two previous episodes [OR(95%CI) = 2.13(1.29-3.51)] was greater than that for three or more compared to less than three prior episodes [OR(95%CI) = 2.06(1.15-3.68)] in CDS (Mueller et al., 1999). Not knowing data on the specific number of

lifetime episodes for the other cohorts is a problem for assessing the nature of the effect of previous episodes on the risk of recurrence. In CDS the reported rate of recurrence was approximately twice as large as for all the other studies reviewed here (approximately 65% in CDS, 31% in GHC and NESDA, 26% in NESARC and 16% in NEMESIS) which might suggest something different about their cohort members relative to the other cohorts, affecting the generalisability of their findings. However, this could equally be due to the fact that CDS reported up to 15 years of follow-up compared to a maximum of six years in NESDA, three years in NEMESIS and NESARC, and only one year in GHC, so it is not surprising that the rate of recurrence across CDS was much larger than for the other studies and that in similar samples the rate in NESDA was approximately twice that in NEMESIS. In consideration of the generalisability across the cohorts it is noteworthy that the populations studied were considerably different: the CDS cohort had much higher proportions of participants with any previous episodes (approximately 67% in Mueller et al., 1999) compared to the NESDA cohort studies (approximately 27% in Hardeveld et al., 2014), and it is likely that in cohorts with high numbers of people with no previous episodes the risk of recurrence across the whole group is much lower over follow up compared to cohorts with high numbers of participants with multiple previous episodes, as a sizeable proportion of those with a first lifetime episode of depression will not experience any future episodes (Eaton et al., 2008; Monroe & Harkness, 2005) so the differences in the findings related to increasing risk with increasing episodes might in part be related to the difference between the risk of recurrence observed in clinical compared to general population samples. Indeed, in CDS there were far higher proportions of inpatients (74% in Solomon et al., 2000) and more severely impaired populations chosen on the basis of their risk for recurrence in GHC, and all of those in GHC and NESARC were seeking or in-treatment, this is compared to NEMESIS and NESDA which drew on community samples of depressed people not seeking or receiving treatment, and a higher proportion from primary care than specialist care centres. There were also potential selection biases impacting upon the reported effects as all studies excluded participants at baseline if they had psychotic conditions but some of the CDS studies kept participants in their samples if their episodes were reclassified later as Bipolar or Schizoaffective and in one of those studies 22% of the recurrences measured were not to MDD but were to Bipolar I (6%) or II disorder (13%), or to Schizoaffective disorder (3%). Further, in the two CDS studies that reported seemingly linear increases in odds of recurrence with increasing episodes they had far fewer participants with four or more and five or more prior episodes than for other comparisons, making calculations of the estimates of the effect less well powered and making summary estimates less accurate. So, whether the effect is an artefact of some features unique to the CDS study or whether it would hold true in other cohorts cannot be determined here. This leads to a question then of whether the finding of this review suggests no linear increase in the probability of recurrence with increasing episodes of MDD, whether there are points at which the number of previous episodes reach a “critical mass” and then lead to a considerable jump in risk (perhaps at three or more episodes), whether or not there is a ceiling to this effect, or whether the reported effect of linearly increasing risk of recurrence with each successive episode is context bound, depending on the population or setting.

Supplementary Figure 7. Flow diagram of study selection in Study 3.

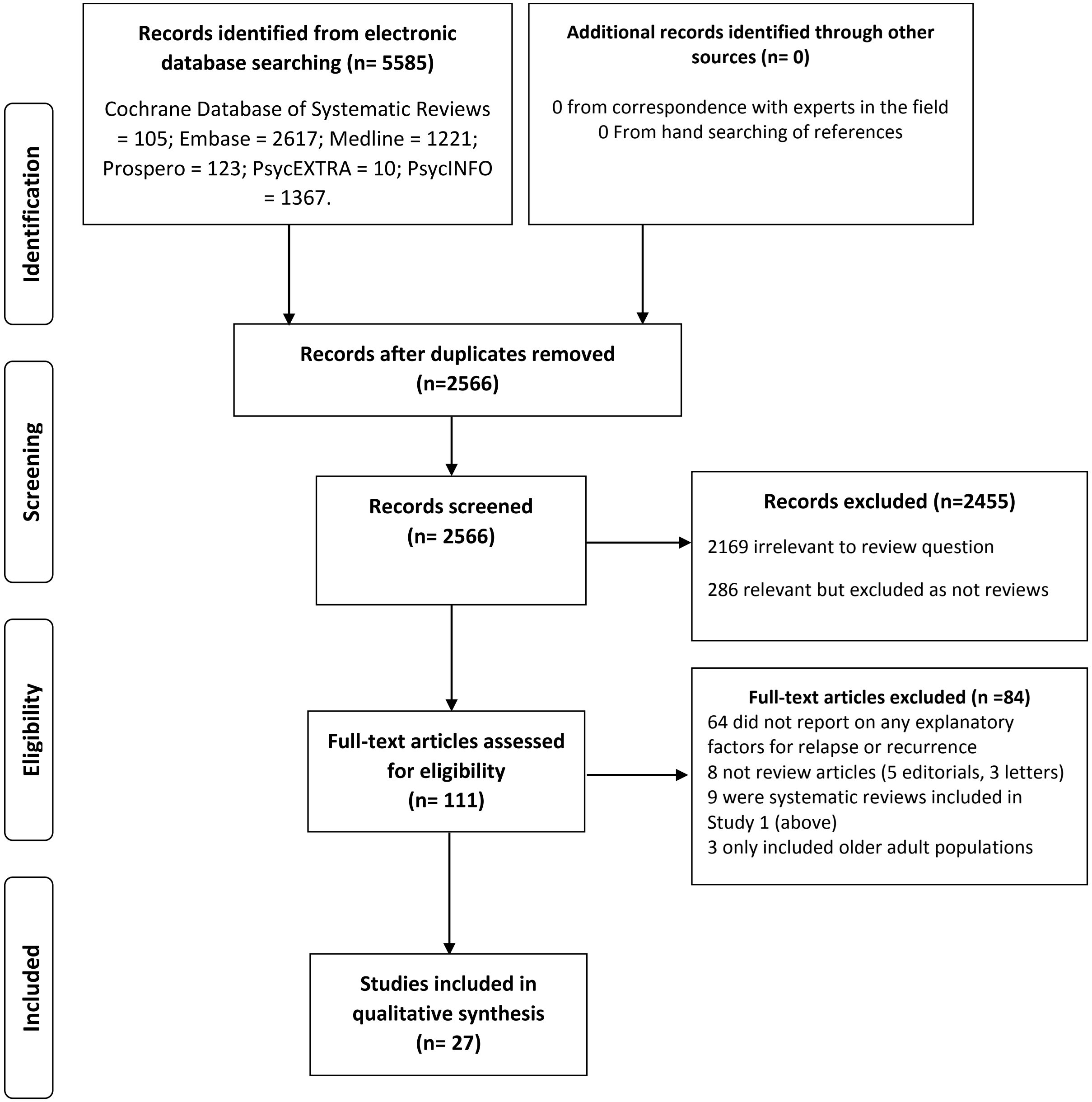

Supplementary Table 7. Data extracted from included non-systematic reviews.

Reviewed Studies	Study design of included studies	Results of relevance to recurrence
Beckerman & Corbett, 2010	Clinical trials	Both thought processing, and poor attentional control might be related to recurrence.
Belsher & Costello, 1988	Cohort studies	Patients are less likely to have a recurrence as the period of recovery increases. Factors increasing risk of recurrence are: recent environmental stress, absence of social support from family members, and a history of depressive episodes, persistent neuroendocrine dysregulation. No significant associations between gender, marital status, or socioeconomic status and recurrence.
Bockting et al., 2015	Clinical trials	Patients with unstable remission, more previous episodes, potentially childhood trauma, or early age of onset are at greatest risk of recurrence, all were prescriptive with all associated with lower risk of recurrence when treated with CBT or MBCT relative to TAU. The role of cognitive reactivity as a risk factor for recurrence is unclear. However, there is substantial evidence linking dysfunctional attitudes with recurrence.
Burcusa & Iacono, 2007	Cohorts, cross-sectional studies and clinical trials	Gender, SES, and marital status are not risk factors for recurrence. Age at onset, number of prior episodes, severity of the first or index episode, comorbid psychopathology especially other affective disorders, and family history of psychopathology, particularly depression or other affective disorders, all associated with increased risk of recurrence. Negative cognitions, high neuroticism, poor social support, and stressful life events may be risk factors for recurrence. Suggested genetic vulnerability to recurrent depression.
Costa e Silva, 2004	Neuroimaging studies, clinical trials, and animal studies	Alteration of metabolism and atrophy of specific neural populations, in brain structures involved in the control of mood and emotions; the hippocampus, the amygdala, and prefrontal cortex, resulting in a decrease in neuroplasticity, may be in the aetiology of depression. The decreased risk of recurrence with maintenance anti-depressant medication may be related to alterations in neuroplasticity.
de Carvalho Tofoli et al., 2011	Neuroimaging studies, experimental studies, quasi-experimental studies, cohort studies, and animal studies	Dysregulation of the HPA axis is partially attributable to an imbalance between GRs (glucocorticoid receptors) and MRs (mineralocorticoid receptors). Evidence has consistently demonstrated that GR function is impaired in major depression, resulting in reduced GR-mediated negative feedback on the HPA axis.
Dedovic & Ngiam, 2015	Cohort studies, cross-sectional studies and experimental studies	Cortisol Awakening Response (CAR) can be used to predict onset of recurrent episodes up to 2.5 years following cortisol measurement. CAR predicts recurrence more strongly than first onset MDD. The magnitude of CAR is similar in first onset compared to recurrent depressive episodes but it is particularly useful in predicting a new onset/recurrence for someone with a history of MDD. Higher CAR is also associated with a quicker time to recurrence after controlling for stressful life events prior to baseline CAR measurement.
Farb et al., 2015	Neuroimaging studies, experimental studies, and some reviews	"Coupling" between dysphoric attention and elaboration increases risk of recurrence as minor stressors can activate dysphoric elaborations. Dysphoric elaboration occurs as a result of continual dysphoric attention, leading to stress sensitisation and a mechanism to recurrence. The authors propose that the two-factor model is related to increased activity in the amygdala, anterior insula, and anterior cingulate cortex, and attenuated activity in the dorsolateral prefrontal cortex.
Hammen, 2003	Cohort studies	Negative interpersonal events brought about by stress generation may increase risk of recurrence in women.
Hick & Chan, 2010	Clinical trials	Cognitive reactivity to sad mood is associated with depressive relapse following successful treatment. Rumination predicts the severity, duration, and recurrence of depressive symptoms. Several studies reported that rumination prolongs and intensifies depression by enhancing the effects of depressed mood on negative thinking.
Hollon & DeRubeis, 2009	Clinical trials	Explanatory style represents an example of the kind of depth cognitions likely to be involved in relapse episodes. Explanatory style also predicted relapse after controlling for treatment type, and is suggested as a mediator of the effect of treatment on risk of recurrence.
Keller, 1996	Cohort studies	From CDS cohort study the most important predictor of recurrence is number of previous episodes. No other factors showed an association with risk of recurrence without first controlling for the number of previous episodes. Probability of recurrence within 6 months rose to 95% for those with 3 or more previous depressive episodes. Other risk factors were: double depression, onset after the age of 60, long duration of individual episodes, family history of affective disorder, comorbid anxiety disorder or substance abuse, and poor symptom control during continuation therapy. This latter factor is particularly important, becoming wholly symptom-free had a profound effect on risk of recurrence.
Kerr et al., 2013	Neuroimaging studies and clinical trials	The effect of Mindfulness practice on reducing the risk of depressive recurrence may function as it enhances attentional control, possibly by acting upon the prefrontal cortical areas known to regulate the thalamocortical circuits.
Kessler, 1997	Cohort studies and case-control studies	Young age at first onset associated with recurrence. Childhood adversity is associated with increased risk of onset before 20 years old. Few of the childhood adversity factors continue to be associated with risk of recurrence. There is no difference in risk of recurrence by gender.
Lau, Segal & Williams, 2004	Experimental and quasi-experimental studies	Increased accessibility and activation of negative thinking patterns induced by sad mood were associated with increased risk of recurrence.

Liu, 2013	Cohort studies, retrospective case-control studies, experimental studies, quasi-experimental studies, cross-sectional studies	Genetic factors are suggested to moderate the relationship between behavioural risk factors and recurrence of depression. Stress generation is tentatively proposed to provide the mechanism for this, but the evidence for this was weak.
Lopresti et al., 2014	Cohort studies, case-control studies, and cross-sectional studies	Raised levels of C-reactive protein (CRP) are associated with increased risk of recurrence. In men but not women a high-sensitivity CRP above 3 mg/L was associated with a four-fold increase in risk of relapse. A meta-analysis of longitudinal studies showed raised CRP levels and to a lesser extent raised Interleukin-6 levels were associated with greater risk of recurrence. Elevated levels of Malondialdehyde have been identified in patients with recurrent depressive episodes and are higher than in those that only had a single MDE. Patients with recurrent depression had bigger reductions in Superoxide Dismutase over time compared to first episode patients.
Metcalf & Dimidjian, 2014	Clinical trials	Post-treatment levels of self-reported mindfulness predicted relapse. Increased cognitive reactivity did not predict relapse for those treated with MBCT but did for those treated with ADMs. Changes in levels of mindfulness did not moderate the relationship between cognitive reactivity and relapse, but self-compassion did.
Modell & Lauer, 2007	Neuroimaging studies, genetic sequencing studies, and animal studies	Dysregulated REM sleep was suggested to be associated with an increased risk of recurrence, suggested mediation by the noradrenergic, serotonergic, and cholinergic systems, and considerable genetic control. Proposed mechanism of CREB gene increasing REM sleep, REM sleep is normally inhibited during brain maturation, a genetic predisposition to the lack of this inhibition may increase risk of depression.
Monroe & Harkness, 2005	Cohort studies, retrospective case-control studies, experimental studies, cross-sectional studies	The stress sensitization and stress autonomy models provide very different accounts of the basic finding that life stress is more important for a first lifetime episode of depression than for a later recurrence. The stress sensitization model appears to provide the more parsimonious account of the existing data. Nonetheless, the stress autonomy model provides an important conceptual alternative. The authors suggest both models play important roles in helping understand recurrence.
Palagini et al, 2013	Neuroimaging studies	REM sleep alterations, particularly shortened REM latencies are proposed as risk factors for recurrence, genetic factors affect risk for these alterations. Genetic factors include cholinergic receptors, circadian rhythm genes and orexinergic mechanisms. Alterations in REM may maintain stress and maladaptive coping with stressful events. ADM effects may be related to suppression of REM sleep.
Robinson & Sahakian, 2008	Experimental and neuroimaging studies	Rumination, negative biases, memory problems and cortisol release all increase with successive episodes but sleep efficacy and social interaction ability diminish. Kindling may explain why first episodes are triggered by stressful life events and lower levels of stress can trigger subsequent episodes.
Scher, Ingram & Segal, 2005	Experimental studies	The one study to directly investigate cognitive reactivity as a predictor of recurrence found that it does increase the risk. This supports the proposal that processes such as attention and interpretive biases play an important role in recurrence.
Scott, 2001	Clinical trials	Patients with residual depressive symptoms were found to have a 50–80% risk recurrence. CT reduced risk of recurrence by changing thinking style not thought content in particular reducing absolutist, dichotomous thinking.
Segal et al., 1996	Experimental studies	Depression-related information processing (mediated by cognitive reactivity) may lead to other depressogenic processes increasing the risk of recurrence.
Sipe & Eisendrath, 2012	Neuroimaging studies, clinical trials, and experimental studies	Rumination, particularly brooding on past failures and anxiety (future-based ruminations) are associated with the course of depression. In Mayberg's model depression is characterized by higher baseline amygdala activity, higher amygdala reactivity to emotional stimuli, and dysfunction between limbic and cortical circuits that regulate affective states.
Slavich & Irwin, 2014	Clinical trials, cohort studies, and cross-sectional studies	Risk for depression increases two-fold for those experiencing social-rejection compared to those experiencing other kinds of interpersonal stress. Some evidence that recurrent MDEs are due to the confluence of social and biological factors such as stress generation excessive reassurance seeking and neurobiological kindling.

Supplementary Figure 8. Flow diagram of studies selected in Study 4.

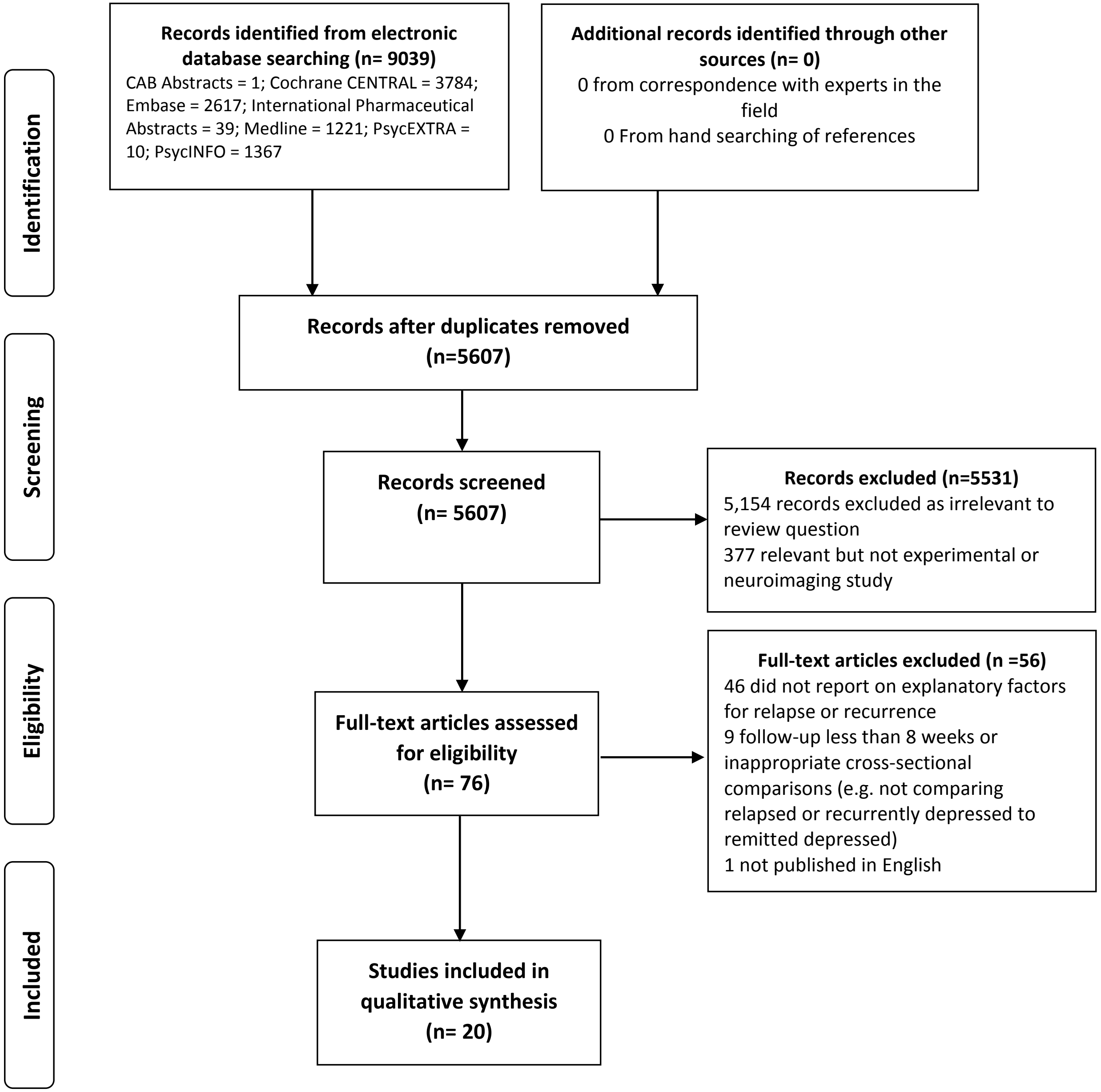

Supplementary Table 8. Data Extracted from Experimental and Neuroimaging Studies.

Reviewed Studies	How Depression Determined at Baseline	Population Characteristics	Intervention details including method of delivery and setting	Comparisons and Ns	Outcomes and How Recorded	Results of relevance to relapse or recurrence
Anderson et al., 2011	SCID and MADRS.	N= 230. Current Depressed mean age 38.6(10.9) mean 6.3(4.4) previous episodes, 53% on ADM; Remitted group mean age 34.2(10.8) 3.0(2.2) previous episodes, 17% on ADM; Controls age 30.3(10.1). 159 female.	Face Emotion Recognition Task. Participants are asked to correctly recognise expressions of anger, disgust, fear, happiness, sadness and surprise with intensities of 30, 50, and 70%, and neutral expression.	Healthy Controls N= 101, Current Depressed N= 30, Remitted Depressed N= 99. **Recurrence only.**	The number of correct items recognised, false positives, and reaction times for each item were recorded.	Remitted group correctly identified more emotions than did controls and those currently depressed. Participants with current depression had impaired accuracy owing to decreased discrimination as to whether an emotion was present (F(2,225) =5.340, p=.005). The effect was mainly driven by the negative emotions anger, fear and sadness (F(5,1125) = 11.900, P50.001).
Arnone et al., 2013	SCID and MADRS.	N= 130, Currently Depressed (CD), Remitted (RD), and Healthy Controls (HC). Mean age 33.8, 72% were females. MADRS scores CD 27.2 (4.3), RD 2.2(2.8), HC 0.6(1.3), Age of Onset CD 22(8.1), RD 25.1(10.8), HC N/A; Past Episodes CD 3.3(3), RD 2.8(2.4). Total Grey Matter Volume CD 0.68(0.07), RD 0.70(0.08), HC 0.70(0.07).	Structural MRI scans at baseline and 8 weeks later, prior to starting Citalopram in the currently depressed group.	Currently Depressed (CD) N =39, Remitted Depressed (RD) N=25, Healthy Controls (HC) N=66. **Uncertain if related only to one of relapse or recurrence, or both.**	Cross-sectional analysis using Voxel-wise analysis from MRI, group plotted against average modulated grey matter per voxel after removing total grey matter volume effects. Longitudinal analysis included whole brain analysis from MRI using average-modulated grey matter.	There was a grey matter reduction in the hippocampi of CD group (of 24-29% vs HC and 17-27% vs RD) with grey matter gain in the same structure following successful ADM treatment. Clusters of grey matter decrease were observed in the parahippocampal gyrus, inferior temporal gyrus, fusiform gyrus, and ventral striatum in unmedicated CD group compared to both the RD and HC groups.
Chen et al., 2014	HRSD	N = 131, First Episode Depressed (FD), Recurrently Depressed (RD), and Healthy Controls (HC). 53.4% Females, mean age = 31.4. HRSD FD 22.6(7.9); RD 23.8(8.9); HC 2.5(1.3); MMSE FD 25.1(1.3); RD 21.5(2.5); HC 29.0(2.2). Age at first onset FD 28.1(2.1); RD 28.3(4.6). Duration of current episode (weeks) FD 28.2(6.2); RD 31.1(8.1). 84% of FD group on ADMs, 100% of RD group.	Emotional Oddball Paradigm - 6 faces, showing neutral emotion with deviations either showing same face with alternative emotion (happy or sad) or a different face showing neutral emotion. 16 blocks of 100 stimuli displayed for 100ms with black screen between; given 1500ms to press a button to say that the face presented was a deviant one.	Formerly Depressed (FD) N=45; Remitted Depressed (RD) N =40, Healthy Controls (HC) N=46. **Recurrence only.**	ERP (Event-related evoked potential - has resolution in the millisecond range and allows assessment of cognitive brain function, used to investigate individuals' information processing of different cognitive schemata) measured with EEG. Reaction time (RT) and accuracy also assessed for each trial.	FD group had slower RT with all faces compared to HCs, RD had slower happy and neutral compared to HC but faster sad face RT compared to HC and FD (F (2, 128)= 14.83, P,0.001). FD and RD were less accurate for happy and neutral faces compared to HC (t's.3.26, P's,0.008), but RD were more accurate than both FD and HC for sad faces (t's.2.32, P's,0.016). N170 amplitude: RD group had lower amplitudes compared to FD and HC. RD had lower amplitude for happy and neutral faces (t's.3.26, P's,0.005), but higher for sad faces compared to FD and HC (t(168) = 3.88, P= 0.004). N170 amplitude was highly correlated with HRSD scores in RD group (F (1,39) =12.02, P,0.001,, deficit in amplitude for sad faces was correlated with the number of previous depressive episodes F (1, 39) = 14.36, P,0.001).

Chen et al., 2015	SCID and HRSD	N = 131, First Episode Depressed (FD), Recurrently Depressed (RD), and Healthy Controls (HC). Mean age 31.4, 53.3% females. HDRS FD 22.6(7.9); RD 23.8(8.9); HC 2.5(1.3); MMSE FD 25.1(1.3); RD 21.5(2.5); HC 29.0(2.2). Age at first onset FD 28.1(2.1); RD 28.3(4.6). Duration of current episode (weeks) FD 28.2(6.2); RD 31.1(8.1). 84% of FD group on ADSM, 100% of RD group.	After preparation for EEG recording participants were presented, through headphones, with 2500 binaural pure tones (1000Hz, 75 dB SPL, 10ms rise/fall) at a 500ms stimulus onset asynchrony; this comprised a pseudo-random sequence of 2300 (92%) 50ms standard tones and 200(8%) 100ms deviant tones (Naismith etal.,2012). Tones were presented while participants watched silent video of a comedy movie (and were asked to report back the story line of the movie at the end of the task).	First Episode Depressed (FD) N=45, Recurrently Depressed (RD) N=40, and Healthy Controls (HC) N=46. **Recurrence only.**	Electro encephalogram (EEG) measurements of 32 scalp locations based on the 10–20 system were recorded using a Brain-Amp MR portable ERP system	RD showed distinct P3a profiles compared to FD, as reflected by the decrease of P3a amplitude and the delay of P3a latencies F (2, 120)=11.38, p<0.001). Furthermore, P3a deficits in the RD group persisted after correcting for the preceding MMN amplitudes F (2, 118)=9.82, p<0.001,partial η2=0.232. Accordingly, these findings suggest that the recurrence of depressive episodes can lead to the impaired pre-attentive information processing. This can then propagate a subsequent bottom-up orienting of attention to the deviant stimulus as the neurophysiological transmission travels from the MMN to the P3a.
Chopra et al., 2008	HRSD and BDI	N= 55 (35 female), mean age 38.8(10.1). 37 patients had 3 or more past depressions.	Listened to ‘‘Russia under the Mongolian Yoke’’, while recalling a sad time in their lives.	Relapsed (28) Vs Non-Relapsed (27). **Mostly recurrence.**	Salivary cortisol samples taken at -25, 0, +25, +45 and +65 minutes relative to mood induction. Change in DAS score to assess relationship between cognitive reactivity and relapse.	Dysfunctional attitudes in response to mood induction predicted relapse (Wald = 4.96, p = .026). Participants with three or more episodes were more likely to relapse if they had low cortisol (Wilcoxon Test = 5.29, d.f. = 1, p = .021). For patients with high cortisol at 25 min rates of relapse were equivalent regardless of past depression history. For patients with low cortisol at 25 min those with no/fewer past episodes of depression had lower rate of relapse.
Dai & Feng, 2011	SCID, HRSD, and BDI	Groups were age and sex matched; mean ages: Normal Controls: 25.71(3.72); Remitted depressed: 27.53(6.36); MDD: 27.59(3.74). 60% of MDD group were outpatients with no comorbid disorders. 6 of MDD and 3 RD taking ADM.	Stroop Colour Word task - 50 positive, neutral and negative words. Ignore meaning respond to colour. Brain EEG activity, HEOG and VEOG tracked.	MDD (N=17), Remitted Depressed (RD) (N=17), Normal Controls (N=17). **Relapse only.**	Word accuracy, false positive rate, reaction time, ERPs and EEG latencies.	MDD participants showed deficient behavioural F(2, 48) = 8.40, p = 0.001) and neurophysiological indices of attentional inhibition for negative words F(2, 48) = 6.20, p = 0.004.. This impaired inhibition of negative information was accompanied by reduced N1 amplitude F(2, 48) = 6.58, p = 0.003 and enhanced N450 component in these trials F(2, 48) = 6.44, p = 0.003) and F(2, 48) = 4.42, p = 0.017.
Franck et al., 2007	MINI interview based on DSM-IV criteria, HRSD and BDI-II-NL.	Mean ages Never Depressed (ND) 45.3(7.3) 8 males, Formerly Depressed (FD)43.8(9.4) 3 males, Currently Depressed (CD) 39.6(12.1) 10 males.	Participants were asked to rate how beautiful they found each letter of the alphabet.	Currently Depressed (N=28), Formerly Depressed (N=34), Never Depressed (N=33). **Mostly recurrence.**	BDI at baseline for all three groups and 6 months follow-up for ND and FD only. RSES at baseline only.	CD patients had lower explicit self-esteem compared to FD t(92)=10.4, p<.001, and ND controls t(92)=10.6, p<.001, and formerly depressed individuals, and ND controls. After controlling for initial symptoms of depression higher levels of implicit self-esteem were associated with higher levels of depressive symptomatology at follow-up t(48)= 2.21, p= .03, b=.25.

Huffziger & Kuehner, 2009	SCID-I and MADRS	Mean ages: Rumination group 51.37(11.82) 50% female, Distraction 45.95(12.96) 52% female, Mindful self-focus 44.85(10.38) 48% female. Groups did not differ on baseline variables all ps> .10.	Sad mood induction with negative autobiographic recall. Randomly assigned to one of three groups (rumination, distraction, or mindful self-focus).	58 remitted patients, 5 with residual symptoms and 13 with current MDD. Compared across induction groups - Rumination (n=24) Distraction (n=27) and Mindfulness (n=25). **Recurrence only.**	Response styles measured with RSQ. Habitual aspects of mindfulness using FMI-14 and mood using PANAS.	Controlling for baseline MADRS, there were differences between induced rumination $F(1,44)= 8.33$, $p= .006$,) and both mindfulness and distraction groups $F(1,47)=6.32$, $p=.015$. Habitual distractive coping predicted less negative $F(1,47)=11.43$, $p <.001$) and more positive mood $F(1,47)=6.32$, $p =.015$) (after Bonferroni corrections the alpha value for significance was .0125). Habitual mindfulness enhanced improvement of negative mood specifically after mindful self-focus $F(1,20)= 3.25$, $p= .086$).
Kronmuller et al., 2008	SCID and HRSD	Of the Currently Depressed Group: 33 female and 24 male patients with mean age at assessment 43.54 (12.82). 27 (47.37%) were married, 22 (38.6%) had a high and 35 (61.4%) a low level of school education; 26 (45.61%) had a first episode of major depression whereas 31 (54.39%) had recurrent depression. Patients with major depression did not differ significantly from healthy controls regarding age, gender, height, weight, handedness, social class, education or alcohol consumption.	Magnetic resonance imaging (MRI) scans of the whole brain were obtained. T1-weighted three-dimensional magnetisation-prepared rapid gradient echo sequences and T2-weighted images were acquired.	Currently Depressed (N=57) and Healthy Controls (N=30). Comparisons were between remitted (N=49) Vs Non-Remitted (N=8) and those experiencing recurrence of depression (N=21) Vs non-recurrence (N=36). **Recurrence only.**	Recurrence was defined according to DSM–IV criteria of major depression, and the LIFE was used	Male patients with a recurrence of depression had significantly smaller ($F(2,24) =4.91$, $p=0.02$), right ($F(2,24) =4.49$, $p=0.02$) and total ($F(2,24) =5.08$, $p=0.01$) hippocampal volumes than healthy male controls. No significant differences were found for women. Even after controlling for current depression, duration of illness, age at onset, number of episodes, severity of illness and medication using ANCOVA, the differences in hippocampal volumes between the two outcome groups remained significant ($F(2,24)=5.08$, $p=.01$).
Lethbridge & Allen, 2008	SCID, BDI, Visual Analogue Scale, DAS	20 males, 32 females, mean age 32.7(11.7). Mean number of previous episodes 1.94(range 1-6). Mean age of onset 24.42(10.25).	Mood induction consisted of autobiographical recall of depressing experience followed by mood-congruent music.	48 of 52 completed follow up. 17 had a recurrence (14 once, 3 twice) over the 12 months. **Recurrence only.**	BDI, SCID, if recurrence occurred then life events questionnaire was also used.	Changes in happy mood following a mood induction were associated with increased risk for recurrence ($\chi^2=8.79$, $p<.01$). Changes in depressed mood following a sad mood induction ($\chi^2=.09$, $p>.05$) and changes in dysfunctional thinking ($\chi^2=.02$, $p>.05$) did not predict recurrence.
Lythe et al., 2015	SCID conducted by a senior psychiatrist	Remitted MDD patients split into: Recurring MDD (RD) and Stable MDD (SD) during 14 month follow-up. Mean number of past episodes RD 3.3(1.8), SD 3.3(3.9). Mean time in remission (months) RD 25.3(21.1), SD 26.6(27.7). Mean length of MDE (months) RD 14.9(21.3), SD 14.3(18.4). Severe depressive episode RD 88%, SD 77%.	fMRI: participants were asked to make emotional judgments about sentences evocative of self-blaming emotions (e.g. “Tom [participant’s name] acts greedily toward Sam [best friend’s name]”) and emotions related to blaming others (ie, other-blame) (eg, “Sam acts greedily toward Tom”).	Comparisons made at baseline between 75 Remitted MDD participants and 39 healthy controls. At follow-up comparisons were between remitted group spilt into RD (N=25) and SD (N=31). **Recurrence only.**	Between-group difference (recurring vs stable MDD) in RSATL connectivity, with an a priori SCSR region of interest for self-blaming vs other-blaming emotions.	The experience of self-blaming relative to other-blaming emotions, connectivity of the RSATL with the SCSR predicted risk of recurring depressive episodes ($F(1,54)=16.23$ $p<.001$). Residual symptoms were associated with neural signatures of recurrence risk, but most of the variance was independent of both residual symptoms and the number of previous episodes. 75% accuracy of predicting recurrence from fMRI measures with no significant predictive value of clinical measures.

Moreno et al., 2000	HAM-D	N=12 all with history of MDE but in remission, and 12 age- and gender-matched controls with no personal or family history of any mental disorder.	Two TRP depletion tests, separated by 1 week. One full-strength and one quarter-strength. Double-blind, crossover design.	MDE over follow-up (N=9 from the 24 participants) compared to no-MDE during follow-up (N=15). **Recurrence only.**	Behavioural ratings obtained at baseline and 5, 7 and 28 hours after depletion. HAM-D was completed weekly for 1 month, and then monthly for 3 months. Retrospective HAM-D and SCID at 6 and 12 months.	An increase of 5 or more in HAM-D score during depletion predicted recurrence. Recurrence was not associated with age, gender, type of past treatment, length of remission, or number of past MDEs.
Morris et al., 2012	SCID-I and BDI-II	N= 68, 32 remitted depressed (RD), and 36 never depressed (ND), mean age =23.39(3.88); 43 were female (63%).	Randomly assigned to high stress or low-stress condition. Cortisol measured after a 10min rest, after a 10min preparation period, after the reading task, and the arithmetic task, after 10mins rest and another 10mins rest.	Remitted Depressed vs Never Depressed. **Mostly recurrence.**	Trier Social Stress Test and salivary cortisol. LIFE administered each week during follow-up. The Perceived Events Scale was used to measure number and severity of stressful life events during follow-up.	Individuals with high-anticipatory stress cortisol and more MDEs were at significantly greater risk of recurrence compared to individuals with low-anticipatory stress cortisol and fewer MDEs (OR(95%CI) = 9.50(1.11-81.14), p=.04). Individuals who showed higher cortisol reactivity to a relatively low-level stressor were at greater risk of experiencing subsequent depressive symptoms .
Nixon et al., 2013	SCID & HAM-D	Controls (n=20) mean age 43(24-63), 30% male, and Patients (n=20) mean age 45(25-63), 35% male.	In fMRI Scanner after baseline images two 10-min trials of a Go/No-Go task with visual negative feedback were administered.	Unmedicated patients, medicated patients, and controls. Also compared recurred (n=7) to non-recurred (n=11). **Recurrence only,**	SCID conducted at 4-montly intervals for 1-year, clinical data gathered from case-notes. Accuracy of responses to task measured.	Hypoactivity in BA 9 of the right dmPFC, believed to play a role in affective regulation was found in patients but not in controls (t(36)=5.33, pFWE-corr=0.001). Some processing of negative experiences at this site may have a protective effect against depression as the dmPFC inhibits ventral, limbic regions allowing for adaptive reappraisal of negative stimuli.
O'Brien-Simpson et al., 2009	SCID and BDI	33 participants assessed in remission from depression, 25 followed-up (19 female, 6 male; age range 20 to 63 years) 2 years later. 9 Ps were on ADM. Duration of remission prior to testing ranged from 73 to 3820 days; Mean= 697 days (SD 903).	Startle probes were bursts of white noise at 100 dB. Startle reflex was measured by surface EMGs. Participants were assessed 1–2 weeks apart. Each time performing an affective picture viewing task, with startle probes. Participants induced to euthymic mood once and depressed mood once.	Relapsed (n=7) Vs Non-relapsed (n=18), and participants that experienced residual depressive symptoms over the follow-up period (n=19) vs those that did not (n=6). **Recurrence only.**	SCID, LIFE used to track symptoms weekly over 2 years and to take monthly measures of social support.	Baseline startle magnitude predicted depressive symptoms and recurrence over the follow-up (F(1, 14)=5.902, p=0.05, $R^2 \approx .30$). Number of past episodes also predicted outcome (F(1, 14)=4.795, p=0.05).
Risch et al., 2010	SCID and BDI	Currently Depressed (N=24): 13 females, mean age 40.2(12.1); Recurrently Depressed (N=28):19 females, mean age 50.1(10.9); Remitted Depressed (N=33) : 20 females, mean age 40.1(13.1); Never Depressed (N=34): 20 females, mean age 44.6(12.7).	Implicit Association Test. Participants were asked to categorise words as fast as possible combined with a self-positive and a self-negative task. Following the IAT completed the BDI and DAS.	First-onset currently depressed patients vs recurrently depressed vs remitted depressed patients vs never depressed. **Recurrence only.**	DAS and IAT	No significant correlation between implicit self-esteem and the number of depressive episodes (ρ= -.29, p=.051, N=33). First onset CD and RCD patients did not significantly differ in implicit self-esteem (t(115)= -2.7, p=.056, Cohen's d=0.7.

Segal et al., 1999	RDC. Remission confirmed by SADS-L interview, HRSD and BDI scores. VAS and DAS also used.	CBT Group (N=25): Mean age 40.5(10.7), 10 males, mean previous episodes 6(10.0); PT Group (N=29), Mean age 39.7(8.0), 12 males, mean previous episodes 3.6(2.1).	Completed VAS then modified Stroop task and then listened to Russia Under the Mongolian Yoke with sad autobiographical recall for 10mins.VAS again after completing Stroop Task.	Remitted depressed CBT Vs PT. Pre induction vs post induction. Recurrence (N=14) Vs non-recurrence (N=16). **Recurrence only.**	DAS. Recurrence determined with SCID and BDI. Only 30 agreed to take part in the follow up, 10 CBT, 20 PT.	The PT group showed a greater increase in cognitive reactivity compared against the CBT group (R^2=.088, F(1,47)=4.80, p<.035). DAS at time of testing predicted recurrence status for 75% of those that did not relapse, and 64.4% of those that did. Changes in DAS after mood induction increases risk of recurrence (χ^2(1,N=30)=4.64, p<.04; final Wald(1)=3.96, p<.05).
Segal et al., 2006	SCID pre-treatment, LIFE and HDRS post-treatment. Completed a DAS and VAS pre-post induction and BDI-II prior to mood-induction.	ADM (N=40), mean age 39.65(11.49), 23 females, 14 first episode, mean previous episodes 1.65(0.48), mean HDRS score 5.23(2.77), mean DAS pre mood-induction 134.73(28.09). CBT group: (N=59) mean age 38.17(10.95), 36 females, 13 first episode, mean previous episodes 1.71(0.46), mean HDRS 5.29(2.80), mean DAS pre mood-induction 128.17(30.04).	ADM condition: treated first-line antidepressant medications for 6 months. CBT condition: 20 individual weekly sessions of CBT. Mood induction using 'Russia under the Mongolian Yoke' played at half speed and recalling a time in their life that made them sad.	99 participants induction and entered an 18-month clinical follow-up. Main comparison was relapsed (n=42) vs non-relapsed (n=57). **Combined relapse and recurrence.**	Patients completed BDI-II and HDRS-17 bimonthly for 18 months post mood-induction. If consistently in depressed range given LIFE. Judged to have relapsed if they were given a diagnosis of MDE at any time during the follow-up. Other outcome measures were DAS and VAS.	After controlling for history of depression cognitive reactivity is a meaningful predictor of relapse/recurrence irrespective of previous treatment modality (Wald χ^2_1=7.48, p=.006, hazard ratio=2.54). Each 16 point increase of the DAS following mood-induction increased the risk of relapse/recurrence by 42%. Patients with marked increases in cognitive reactivity had higher relapse/recurrence rates (69%) than those with minimal increases in cognitive reactivity (30%) or with marked decreases in cognitive reactivity (32%).
Serra-Blasco et al., 2016	HRSD and diagnosis by Psychiatrist	N=49. At baseline: mean age 47.78(7.7), 77.6% female, 46.9% University education, 55.1% Married. Clinical Characteristics: 19 First MDD episode, 20 with >=3 past episodes but in remission at baseline, 10 with Treatment Resistant Depression at baseline; HRSD mean 14.7(10.2), mean age of onset 35(11.7), meant duration of depression 139.2(151.2) months, mean number of past MDEs 3.6(4.9), 98% on ADM, 14.3% on Antipsychotics, 61.2% on Anxiolytics.	Structural MRI at baseline with 5 year follow-up with clinical interview conducted by a psychologist attached to hospital psychiatry department where MRI was originally conducted	At baseline: First Episode Depressed N=19, >=3 Previous Depressive Episodes in Remission N=20, Treatment Resistant Depression N=10. **Recurrence only.**	At 5 year follow-up - clinical outcomes were assessed by clinical interview with Life-Chart Manual for Recurrent Affective Illness (for Clinicians Retrospective Version: LCM-C/R) and results were cross-checked against hospital database and with treating psychiatrist. Clinical outcomes were split into Recovered with no recurrences N=10; Partial Remitted with no recurrence N=7, Remitted then recurrence N=14; and Chronic Depression over follow-up N=18.	Regression analyses of clinical outcomes: Parameter estimates included baseline duration of illness (OR(95%CI)=0.007(0.0001–0.14), p= .044), HDRS (OR(95%CI)= 0.14(0.03–0.25), p= .013), and number of previous MDD episodes (OR(95%CI)= 0.762(−0.117-1.641), p= .089), together with right inferior frontal gyrus (OR(95%CI)= 0.002(0.000057–0.003), p= .042), anterior cingulate (OR(95%CI)=0.003(−0.005-−0.001), p= .005), and right middle frontal gyrus (OR(95%CI)= −0.001(−0.002-0.003), p= .066). Although not independent risk factors when taken together longer illness duration, more severe depressive symptoms at baseline and the greater number of previous depressive episodes were predictive of worse outcomes including recurrence and chronic course at five year follow-up (combined). Adding structural MRI data, particularly Gray Matter Volumes of the right anterior cingulate and right inferior frontal gyrus increased the predictive capacity of the models with approximately 20% more variance explained compared to models of just demographic and clinical data alone.

Watkins & Baracaia, 2002	SCID and BDI	Currently Depressed N=32: mean age 42.3(12.8), 24 females. 81.2% on ADM, current episode length mean 8.7(9.2) months, age at first onset 21.5(8.3), mean previous episodes 6.2(3.4). Recovered Depressed N=26: Mean age 41.8(9.9), 18 females. 15.4% currently on ADM, mean age of first onset 27.6(12.9) and mean previous episodes 3.1(2.6). Never Depressed N=26: mean age 36.1(12.2), 16 females, none currently on ADM.	Randomly allocated to the conditions (no questions, n=27; state-oriented, n=29; process focused, n=28). Participants were presented with scenarios and asked to think of best solution to each one. They were presented with extra material based on their allocated condition.	Within-groups comparisons were made based on condition. Between groups: Currently Depressed (N=32), Recovered Depressed (N=26) and Never Depressed (N=26). **Recurrence only.**	BDI, Mood measure rating mood 0 (I do not feel at all despondent) to 100 (I feel extremely despondent). RRS measuring how often one responds to a sad mood with rumination. The Means-Ends Problem-Solving Test (MEPS) measuring ability to conceptualise step-by-step means of achieving a goal.	Within the no question condition: The currently depressed group produced significantly less effective solutions than the recovered depressed ($p<.01$) and never depressed groups ($p<.05$). In the state-oriented condition: Both the currently depressed ($p<.005$ for means, $p=.015$ for solutions) and recovered depressed ($p<.05$ for means, and $p<.05$ for solutions) groups produced significantly fewer relevant means and less effective solutions than the never depressed group. Within the process-focussed condition: there was no effect of group. The currently depressed group were more despondent than the recovered depressed and never depressed groups.

Abbreviations: ADM – antidepressant medication; ANOVA – analysis of variance; BA – Brodmann's area; BAI – Beck Anxiety Inventory; BDI/BDI-II/BDI-II-NL – Beck Depression Inventory; CAS – clinical anxiety scale; CBT - cognitive behaviour therapy; CD – currently depressed; CH – correct hit; CI – confidence interval; CSI –chronic somatic illness; DAS – Dysfunctional attitude scale; db – decibel; df – degrees of freedom; dmPFC – dorsal medial pre-frontal cortex; DP – double button press; DSM-IV/DSM-III – Diagnostic and Statistical Manual of Mental Disorders 4th/3rd Edition; EC – error commission; EEG – electroencephalography; EMG – electromyography; ERP – event related potentials; FD – formerly depressed; FMI-14 Freiburg Mindfulness Inventory; HAM-D – Hamilton depression rating scale; HEOG – horizontal electrooculography; HRSD - Hamilton rating scale for depression; IAT – implicit association test; ICD-10 – International Classification of Diseases 10th Edition; LIFE – Longitudinal interval follow-up evaluation; MADRS – Montgomery-Asberg Depression Rating Scale; MBCT –Mindfulness based cognitive therapy; MDD – major depressive disorder; MDE – major depressive episode; MEPS – Means ends problem solving test; MMN – Mismatch Negativity (an ERP component); ms – millisecond; NC – never disordered controls; ND – never depressed; NLPT - RCT – randomised controlled trial; P3a (or P3000) – an ERP component; PANAS – Positive and Negative Affect Schedule; PSR – psychiatric status rating; PT – pharmacotherapy; rACC – right anterior cingulate cortex; RCD – recurrently depressed; RDC – research diagnostic criteria; RMD – remitted depressed; RRS – Ruminative response scale; RSATL – Right superior anterior temporal lobe; RSES- Rosenberg Self-Esteem Scale; RSQ - Response Styles Questionnaire; SADS-L – schedule for affective disorders and schizophrenia lifetime version; SCID – Structured Clinical Interview for DSM-IV; SCSR – Subgenual cingulate cortex and the adjacent septal region; SD – standard deviation; TRP – tryptophan; VAS – visual analogue scale; VEOG – vertical electrooculography; VNF - visual negative feedback.

Supplementary Table 9. Quality Rating of Experimental and Neuroimaging Studies.

**Study Citation**	Question/objective sufficiently described?	Study design evident and appropriate?	Method of group selection described and appropriate?	Subject (and comparison group) characteristics sufficiently described?	Random allocation procedure described?	Blinding of investigators reported?	Blinding of subjects reported?	Outcome and exposure measure(s) well defined and robust?	Sample size appropriate?	Analytic methods justified and appropriate?	Estimate of variance reported for the main results?	Controlled for confounding?	Results reported in sufficient detail?	Conclusions supported by the results?	Total Score	Maximum Possible score: 28-(number of N/A Items x2)	Total Score / Maximum Possible Score	*Proposed Quality Category*
Anderson et al., 2011	1	2	2	2	N/A	N/A	N/A	2	1	1	0	0	2	1	14	22	0.64	Moderate
Arnone et al., 2013	2	2	2	2	N/A	N/A	N/A	2	1	1	0	1	1	2	16	22	0.73	Moderate
Chen et al., 2014	2	2	1	2	N/A	N/A	N/A	2	1	2	0	1	2	2	17	22	0.77	High
Chen et al., 2015	2	2	1	2	N/A	N/A	N/A	2	1	1	0	1	2	2	16	22	0.73	Moderate
Chopra et al., 2008	1	2	1	1	1	N/A	N/A	2	1	2	0	2	2	2	17	24	0.71	Moderate
Dai & Feng, 2011	2	2	1	2	N/A	2	N/A	2	1	2	0	1	2	2	19	24	0.79	High
Franck et al., 2007	2	2	2	2	N/A	N/A	N/A	2	1	2	0	0	2	2	17	22	0.77	High
Huffziger & Kuehner, 2009	2	2	1	2	2	0	0	2	1	2	0	0	2	2	18	28	0.64	Moderate
Kronmuller et al., 2008	1	2	1	2	N/A	N/A	N/A	2	1	1	0	0	2	1	13	22	0.59	Moderate
Lethbridge & Allen, 2008	2	2	1	2	N/A	N/A	N/A	1	2	1	2	2	2	2	17	22	0.77	High
Lythe et al., 2015	2	2	2	2	N/A	N/A	N/A	2	1	2	2	1	2	2	20	22	0.91	High
Moreno et al., 2000	0	1	0	1	1	0	0	1	1	1	0	0	1	2	9	28	0.32	Low
Morris et al., 2012	2	2	1	2	1	0	0	2	1	2	2	2	2	2	21	28	0.75	Moderate
Nixon et al., 2013	2	2	2	2	N/A	N/A	N/A	2	1	2	2	1	2	2	20	22	0.91	High
O'Brien-Simpson et al., 2009	2	2	1	2	N/A	N/A	N/A	2	1	2	0	2	2	2	18	22	0.82	High
Risch et al., 2010	2	2	1	2	N/A	N/A	N/A	2	1	2	0	0	2	2	16	22	0.73	Moderate
Segal et al., 1999	2	2	1	2	N/A	N/A	N/A	2	1	2	0	0	2	2	16	22	0.73	Moderate
Segal et al., 2006	2	2	2	2	1	0	0	2	2	2	1	2	2	2	22	28	0.79	High
Serra-Blasco et al., 2016	2	2	2	2	N/A	N/A	N/A	2	1	2	2	2	2	2	21	22	0.95	High
Watkins & Baracaia, 2002	2	2	1	2	1	1	0	2	1	2	0	1	2	2	19	28	0.68	Moderate

Additional References in Supplementary Materials Only

Dimidjian, S., Hollon, S. D., Dobson, K. S., Schmaling, K. B., Kohlenberg, R. J., Addis, M. E., ... & Atkins, D. C. (2006). Cognitive Behavioural Therapy for Mood Disorders. *Journal of Consulting and Clinical Psychology, 74,* 658-670.

Elkin, I., Shea, M. T., Watkins, J. T., Imber, S. D., Sotsky, S. M., Collins, J. F., ... & Fiester, S. J. (1989). National Institute of Mental Health treatment of depression collaborative research program: General effectiveness of treatments. *Archives of General Psychiatry, 46*(11), 971-982.

Fournier, J. C., DeRubeis, R. J., Shelton, R. C., Gallop, R., Amsterdam, J. D., & Hollon, S. D. (2008). Antidepressant medications v. cognitive therapy in people with depression with or without personality disorder. *British Journal of Psychiatry, 192*(2), 124-129.

Ma, S. H., & Teasdale, J. D. (2004). Mindfulness-based cognitive therapy for depression: replication and exploration of differential relapse prevention effects. *Journal of Consulting and Clinical Psychology, 72*(1), 31-40.

Teasdale, J. D., Segal, Z. V., Williams, J. M. G., Ridgeway, V. A., Soulsby, J. M., & Lau, M. A. (2000). Prevention of relapse/recurrence in major depression by mindfulness-based cognitive therapy. *Journal of Consulting and Clinical Psychology, 68*(4), 615-623.

Additional References in Review Tables Only

Anderson, I. M., Shippen, C., Juhasz, G., Chase, D., Thomas, E., Downey, D., et al. (2011). State-dependent alteration in face emotion recognition in depression. *The British Journal of Psychiatry*, *198*(4), 302–308.

Belsher, G., & Costello, C. G. (1988). Relapse after recovery from unipolar depression: A critical review. *Psychological Bulletin, 104*(1), 84-96.

Bockting, C., Hollon, S. D., Jarrett, R. B., Kuyken, W., & Dobson, K. (2015). A lifetime approach to major depressive disorder: The contributions of psychological interventions in preventing relapse and recurrence. *Clinical Psychology Review, 41*, 16-26.

de Carvalho Tofoli, S. M., Baes, C. V. W., Martins, C. M. S., & Juruena, M. (2011). Early life stress, HPA axis and depression. *Psychology & Neuroscience, 4*(2), 229-234.

Chopra, K. K., Segal, Z. V, Buis, T., Kennedy, S. H., & Levitan, R. D. (2008). Investigating associations between cortisol and cognitive reactivity to sad mood provocation and the prediction of relapse in remitted major depression. *Asian Journal of Psychiatry*, 1(2), 33-36.

Costa e Silva, J. A. (2004). From restoration of neuroplasticity to the treatment of depression: Clinical experience. *European Neuropsychopharmacology, 14*(S5), S511-S521.

Dai, Q., & Feng, Z. (2011). Deficient interference inhibition for negative stimuli in depression: An event-related potential study. *Clinical Neurophysiology, 122*(11), 52-61.

Dedovic, K., & Ngiam, J. (2015). The cortisol awakening response and major depression: examining the evidence. *Neuropsychiatric Disease and Treatment, 11*, 1181-1189.

Franck, E., De Raedt, R., & De Houwer, J. (2007). Implicit but not explicit self-esteem predicts future depressive symptomology. *Behaviour Research and Therapy, 45*(10), 2448-2455.

Hammen, C. (2003). Interpersonal stress and depression in women. *Journal of Affective Disorders, 74*(1), 49-57.

Hick, S. F., & Chan, L. C. (2010). Mindfulness-Based Cognitive Therapy for Depression: Effectiveness and limitations. *Social Work in Mental Health, 8*(3), 225-237.

Hollon, S. D., & DeRubeis, R. J. (2009). Mediating the effects of cognitive therapy for depression. *Cognitive Behaviour Therapy, 38*(S1), 43-47.

Huffziger, S., & Kuehner, C. (2009). Rumination, distraction, and mindful self-focus in depressed patients. *Behaviour Research and Therapy, 47*(3), 224-230.

Keller, M. B. (1996). Depression: Considerations for treatment of a recurrent and chronic disorder. *Journal of Psychopharmacology, 10*(S1), 41-44.

Kerr, C. E., Sacchet, M. D., Lazar, S. W., Moore, C. I., & Jones, S. R. (2013). Mindfulness starts with the body: Somatosensory attention and top-down modulation of cortical alpha rhythms in mindfulness meditation. *Frontiers in Human Neuroscience, 7*(12), 1-15.

Kessler, R. C. (1997). The effects of stressful life events on depression. *Annual Review of Psychology, 48*, 191-214.

Lau, M. A., Segal, Z. V., & Williams, J. M. G. (2004). Teasdale's differential activation hypothesis: Implications for mechanisms of depressive relapse and suicidal behaviour. *Behaviour Research and Therapy, 42*(9), 1001-1017.

Lethbridge, R., & Allen, N. B. (2008). Mood induced cognitive and emotional reactivity, life stress, and the prediction of depressive relapse. *Behaviour Research and Therapy, 46*(10), 1142-1150.

Liu, R. T. (2013). Stress generation: Future directions and clinical implications. *Clinical Psychology Review, 33*(3), 406-416.

Lopresti, A. L., Maker, G. L., Hood, S. D., & Drummond, P. D. (2014). A review of peripheral biomarkers in major depression: the potential of inflammatory and oxidative stress biomarkers. *Progress in Neuro-Psychopharmacology and Biological Psychiatry*, *48*, 102-111.

Metcalf, C. A., & Dimidjian, S. (2014). Extensions and Mechanisms of Mindfulness-based Cognitive Therapy: A Review of the Evidence. *Australian Psychologist*, *49*(5), 271-279.

Modell, S., & Lauer, C. J. (2007). Rapid eye movement (REM) sleep: An endophenotype for depression. *Current Psychiatry Reports, 9*(6), 480-485.

Moreno, F. A., Heninger, G. R., McGahuey, C. A., & Delgado, P. L. (2000). Tryptophan depletion and risk of depression relapse: A prospective study of tryptophan depletion as a potential predictor of depressive episodes. *Biological Psychiatry, 48*(4), 327-329.

Morris, M. C., Rao, U., & Garber, J. (2012). Cortisol responses to psychosocial stress predict depression trajectories: Social-evaluative threat and prior depressive episodes as moderators. *Journal of Affective Disorders, 143*(1-3), 223-230.

O'Brien-Simpson, L., Di Parsia, P., Simmons, J. G., Allen, N. B. (2009). Recurrence of major depressive disorder is predicted by inhibited startle magnitude while recovered. *Journal of Affective Disorders, 112*(1-3), 243-249.

Palagini, L., Baglioni, C., Ciapparelli, A., Gemignani, A., & Riemann, D. (2013). REM sleep dysregulation in depression: State of the art. *Sleep Medicine Reviews, 17*(5), 377-390.

Robinson, O. J., & Sahakian, B. J. (2008). Recurrence in major depressive disorder: a neurocognitive perspective. *Psychological Medicine, 38*(3), 315-318.

Scher, C. D., Ingram, R. E., & Segal, Z. V. (2005). Cognitive reactivity and vulnerability: Empirical evaluation of construct activation and cognitive diatheses in unipolar depression. *Clinical Psychology Review, 25*(4), 487-510.

Scott, J. (2001). Cognitive therapy for depression. *British Medical Bulletin, 57*(1), 101-113.

Segal, Z. V., Gemar, M., & Williams, S. (1999). Differential cognitive response to a mood challenge following successful cognitive therapy or pharmacotherapy for unipolar depression. *Journal of Abnormal Psychiatry, 108*(1), 3-10.

Segal, Z., Williams, J. M., Teasdale, J. D., & Gemar, M. (1996). A cognitive science perspective on kindling and episode sensitization in recurrent affective disorder. *Psychological Medicine, 26*(2), 371-380.

Sipe, W. E. B., & Eisendrath, S. J. (2012). Mindfulness-based cognitive therapy: Theory and practice. *The Canadian Journal of Psychiatry, 57*(2), *63-69.*

Slavich, G. M., & Irwin, M. R. (2014). From stress to inflammation and major depressive disorder: A social signal transduction theory of depression. *Psychological bulletin*, *140*(3), 774.
